# Shortwave infrared-absorbing squaraine dyes for all-organic optical upconversion devices

**DOI:** 10.1080/14686996.2021.1891842

**Published:** 2021-04-13

**Authors:** Karen Strassel, Wei-Hsu Hu, Sonja Osbild, Daniele Padula, Daniel Rentsch, Sergii Yakunin, Yevhen Shynkarenko, Maksym Kovalenko, Frank Nüesch, Roland Hany, Michael Bauer

**Affiliations:** a Laboratory for Functional Polymers, Empa, Swiss Federal Laboratories for Materials Science and Technology, Dübendorf, Switzerland; b Ecole Polytechnique Fédérale de Lausanne, EPFL, Institute of Chemical Sciences and Engineering, Lausanne, Switzerland; c Ecole Polytechnique Fédérale de Lausanne, EPFL, Institute of Materials Science and Engineering, Lausanne, Switzerland; d Dipartimento di Biotecnologie, Chimica e Farmacia, Università di Siena, Siena, Italy; e Department of Chemistry and Applied Biosciences, Laboratory of Inorganic Chemistry, Zürich, Switzerland; f Laboratory for Thin Films and Photovoltaics, Empa, Swiss Federal Laboratories for Materials Science and Technology, Dübendorf, Switzerland

**Keywords:** Squaraine dye, shortwave infrared, organic upconverter, organic photodetector, 40 Optical, magnetic and electronic device materials, 201 Electronics / Semiconductor / TCOs, 204 Optics / Optical applications, 301 Chemical syntheses / processing, 306 Thin film / Coatings, 501 Chemical analyses

## Abstract

Shortwave infrared (SWIR) optical sensing and imaging are essential to an increasing number of next-generation applications in communications, process control or medical imaging. An all-organic SWIR upconversion device (OUC) consists of an organic SWIR sensitive photodetector (PD) and an organic light-emitting diode (OLED), connected in series. OUCs directly convert SWIR to visible photons, which potentially provides a low-cost alternative to the current inorganic compound-based SWIR imaging technology. For OUC applications, only few organic materials have been reported with peak absorption past 1000 nm and simultaneous small absorption in the visible. Here, we synthesized a series of thermally stable high-extinction coefficient donor-substituted benz[*cd*]indole-capped SWIR squaraine dyes. First, we coupled the phenyl-, carbazole-, and thienyl-substituted benz[*cd*]indoles with squaric acid (to obtain the SQ dye family). We then combined these donors with the dicyanomethylene-substituted squaraine acceptor unit, to obtain the dicyanomethylene-functionalized squaraine DCSQ family. In the solid state, the absorbance of all dyes extended considerably beyond 1100 nm. For the carbazole- and thienyl-substituted DCSQ dyes, even the peak absorptions in solution were in the SWIR, at 1008 nm and 1014 nm. We fabricated DCSQ PDs with an external photon-to-current efficiency over 30%. We then combined the PD with a fluorescent OLED and fabricated long-term stable OUCs with peak sensitivity at 1020 nm, extending to beyond 1200 nm. Our OUCs are characterized by a very low dark luminance (<10^−2^ cd m^−2^ at below 6 V) in the absence of SWIR light, and a low turn-on voltage of 2 V when SWIR light is present.

## Graphical abstract

**Figure:**
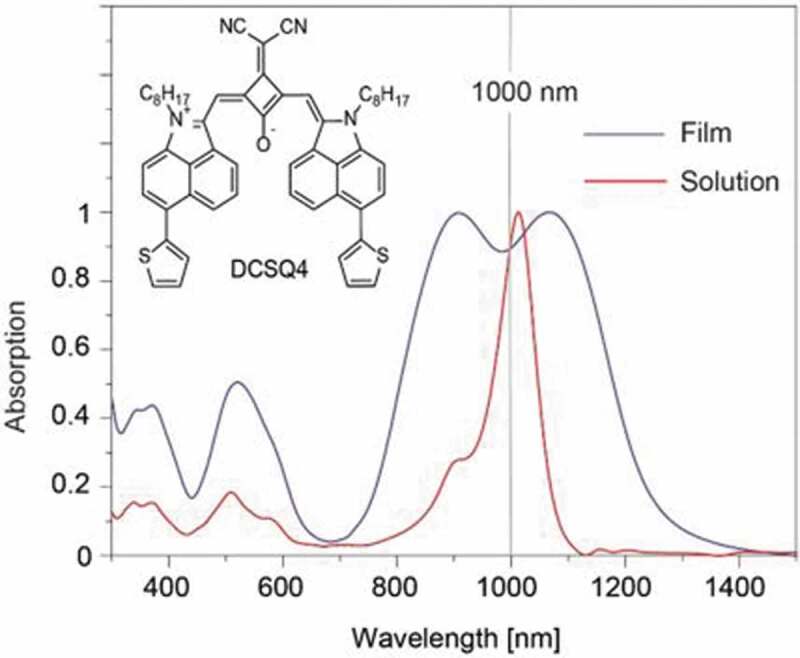

## 1. Introduction

SWIR photodetection and imaging offer new application fields in passive night vision, airborne remote sensing or machine vision solutions, including silicon wafer inspection, product quality control and sorting [1,2]. In bio-imaging applications, the so-called second biological window between 1000 nm and 1700 nm allows for deep penetration of light with low auto-fluorescence and high spatial resolution, because absorption and scattering from (de-)oxygenated blood, skin and tissue is low compared to the first biological window between 700 nm and 950 nm. The great benefits of SWIR light for deep-tissue bioimaging could be revealed exploring fluorescent carbon nanotubes, rare-earth materials, quantum dots and organic materials emitting above 1000 nm [3–8].

The SWIR band matches the spectral sensitivity range of the semiconductor compound indium gallium arsenide (InGaAs). Most SWIR cameras have an InGaAs sensor and the highest performance ones typically detect light between around 900 nm and 1700 nm [9]. InGaAs sensor arrays are still cost prohibitive for most consumer and low-end applications, although the growing market and improvements in the sensor fabrication have resulted in a decrease of the technology costs [10].

A SWIR-to-visible upconversion device, also named upconversion PD [11], upconversion OLED [12] or SWIR visualization device [13], is made by integrating an SWIR PD with a visible light-emitting unit. Such devices potentially offer an alternative route to true low-cost, pixel-free SWIR imaging. The basic idea of any upconverter is that photocurrent generated in the SWIR PD layer drives the serial connected visible light-emitting unit. Upconversion devices convert low-energy SWIR photons directly into a visible image, avoiding intermediate electronics and an external display for image visualization. Note that the functionality of an upconversion device is different from the several known photon upconversion processes. Photon upconversion describes a process that converts two or more sequentially absorbed low-energy photons into a photon of higher energy.

The status and progress until 2018 for optical upconverters that are entirely made with organic and hybrid materials [14], including perovskites and quantum dots, is summarized in reference [15]. Since then, several solution-processed upconverters based on quantum dots were reported [11,16,17]. A colloidal lead sulfide quantum dot layer harvested the near-infrared (NIR)/SWIR light, and a cadmium selenide quantum dot layer was used for visible light emission. In one example, the device detected SWIR photons out to 1600 nm and the NIR photon-to-visible photon conversion efficiency (940 nm to 525 nm) was 6.5% [11]. A broad-band absorbing polymer-based PD was combined with a phosphorescent OLED and the device sensitivity extended to 1100 nm [12]. In a similar manner, an upconverter was demonstrated by monolithic integration of a low bandgap polymer:SWIR dye blend PD with sensitivity out to around 1400 nm and a perovskite light-emitting (at 516 nm) diode [13]. Cyanine dye-based PDs with NIR-selective absorption between 600 nm and 1000 nm were integrated with a fluorescent OLED. Devices converted light at 830 nm to green light, and the luminance turn-on was at a low voltage of 2 V [18].

For an all-organic SWIR upconverter (OUC) it is advantageous to use an organic PD material with selective absorption in the SWIR region. This is because visible light absorption of a broad-band absorber material results in a non-selective response of the device, and visible emitted light from the OLED can be reabsorbed by the PD unit. While low-bandgap organic materials with broad-band absorption extending out to around 1700 nm are known [19–22], relatively few organic materials with selective SWIR absorption have been reported [23–25]. As an extension to a recent review on NIR absorbing organic dyes [26], a list of representative dyes with peak absorption in the SWIR region, i.e. not merely a tail in the absorption spectrum extending beyond 1000 nm, is compiled in the Supporting Information, Table S1. Most of the dyes belong to the families of cyanines [27], rylenes [28] or are charge-transfer chromophores [29]. For optoelectronic device applications, these SWIR dyes possess some potential drawbacks, such as a decreased photo- and thermal stability as well as incompatibility with neutral electron acceptors in blend films (cyanines), limited solubility (rylenes) or low extinction coefficients (charge-transfer dyes).

Here, we report the synthesis and OUC device integration of SWIR squaraine dyes. Squaraines are known for their straightforward and scalable synthesis, narrow and intense absorption and emission properties in the NIR [30,31], as well as good thermal- and photostability [30,32,33]. The squaraine family is a large material library suitable for a variety of applications in chemical sensing, optoelectronic devices, photodynamic therapy and bioimaging [30,31,34,35].

Squaraines contain an electron-deficient central four-membered ring and two electron-donating groups in a donor-acceptor-donor configuration with a resonance stabilized π-conjugated zwitterionic structure. Appropriate choice of the donor and acceptor moieties allows tuning the optical properties of the dyes, and the combination of strong donors with strong acceptors as well as a high degree of conjugation leads to a bathochromic shift of the absorption maximum [36,37].

Recently, we investigated the synthesis and properties of the symmetrical benz[*cd*]indolium capped squaraine dye, in this work referred to as **SQ1**. The dye exhibits strong absorption in the NIR and we demonstrated visibly transparent and solution-processed OUCs with peak sensitivity at 980 nm [38,39]. Here, we expand the family of SQ dyes and increased the donor strength of the benz[*cd*]indolium unit by coupling with phenyl-, carbazole-, and thiophene moieties at the 6-position of the substituent. In a second series of squaraines (the DCSQ family), we coupled theses donors with the dicyanomethylene-substituted squaraine acceptor unit. We found that the carbazole- and thiophene-substituted DCSQ dyes in solution have peak absorptions above 1000 nm, and therefore represent the first SWIR-absorbing squaraine dyes, to the best of our knowledge. We then fabricated DCSQ-fullerene PDs with a maximum external photon-to-current conversion efficiency (EQE) at 1025 nm. By combining this PD with a fluorescent Alq_3_-based OLED, OUCs were obtained that convert SWIR photons directly to visible green photons, with good performance in terms of a low turn-on voltage, a low dark luminance and a highly linear optical response.

## 2. Results and discussion

### 2.1. Synthesis

**Scheme 1.**
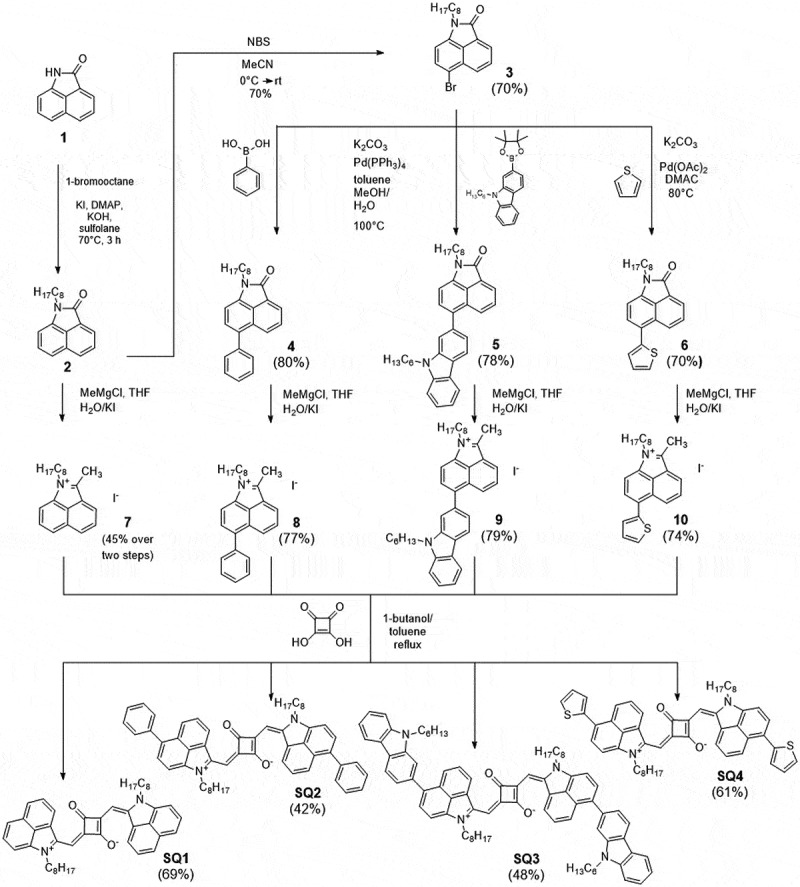
Synthesis of squaraine dyes **SQ1** – **SQ4.**

To study the influence of the donor substitution on the properties of the squaraine dyes, four different donor building blocks based on the benz[*cd*]indole heterocycle were synthesized as shown in Scheme 1. Lactam **1** was first *N*-alkylated using octylbromide to yield compound **2** that was used as an intermediate for the brominated lactam **3** and the unsubstituted iminium iodide **7**. The bromine-functionality allowed the introduction of different (hetero-)aromatic substituents. The phenyl- and carbazole-substituted benz[*cd*]indoles **4** and **5** were synthesized via a Suzuki type cross-coupling reaction. The thiophene-substituent (**6**) was introduced in a direct heteroarylation (DHA) coupling using excess thiophene, potassium carbonate as base and palladium(II) acetate as catalyst. The DHA coupling was chosen due to its high atom economy, cheap reactants, and avoidance of toxic organometallic precursors. Methylation of compounds **2, 4, 5**, and **6** with methyl magnesium chloride, followed by a condensation and an iodine ion exchange reaction lead to the formation of iminium iodides **7**–**10** in 45% – 79% isolated yield.

The squaraine dyes **SQ1 – SQ4** were synthesized according to an adapted procedure from literature [40]. Iminium salts **7–10** were condensed with squaric acid by refluxing in a mixture of *n*-butanol and toluene. Recrystallization from ethanol resulted in the corresponding dyes in yields of 42% – 69%.

For **SQ1** – **SQ4**, six different stereoisomers are conceivable (Figure S1). In Scheme 1, the dyes are drawn as the most stable ‘trans-anti-out’ isomer. Here, the heterocycles are attached on opposite sides of the polymethine chain (‘trans’), the nitrogen atoms of the benz[*cd*]indolium cycle are facing in opposite directions (‘anti’), both away from the squaric acid core (‘out’). A reversible *trans-cis* exchange process could be observed in solution. From NMR experiment we concluded that in these cases a minor SQ isomer (cis-syn-out) was present, in agreement with a population analysis using DFT calculations (SI, isomers of squaraine dyes). The occurrence of squaraine isomers has been reported on several occasions [41–43].

**Scheme 2.**
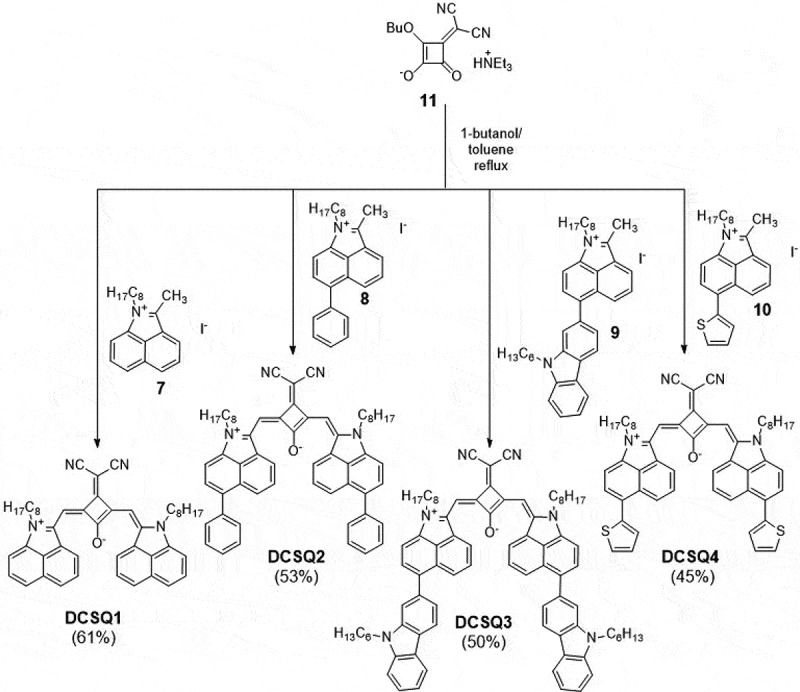
Synthesis of dicyanomethylene-substituted squaraine dyes **DCSQ1** – **DCSQ4.**

Dicyanomethylene-substituted squaraine dyes **DCSQ1 – DCSQ4** were synthesized by condensation of dicyano squarate **11** and iminium iodides **7–10** in a mixture of *n*-butanol and toluene that was heated to 130°C – 140°C for 2–4 hours (Scheme 2) [37]. Dyes were isolated after purification by column chromatography and precipitation from DCM/*n*-heptane in 45% – 61% yield. The steric demand of the dicyanomethylene group forces the DCSQ dyes into the cis-syn-out conformation and no other isomers were detectable from NMR spectra. The synthesis of the *N*-ethyl-substituted **DCSQ1** derivative was reported recently [44]. In that case, the dye was synthesized via a stepwise condensation of the iminium salt with the diethyl squarate, followed by introduction of the dicyanomethylene group to the semisquaraine, and finally the condensation with the iminium salt. Experimental details for the synthetic procedure of all our dyes are compiled in the SI.

### 2.2. Molecular properties

**Figure 1.**
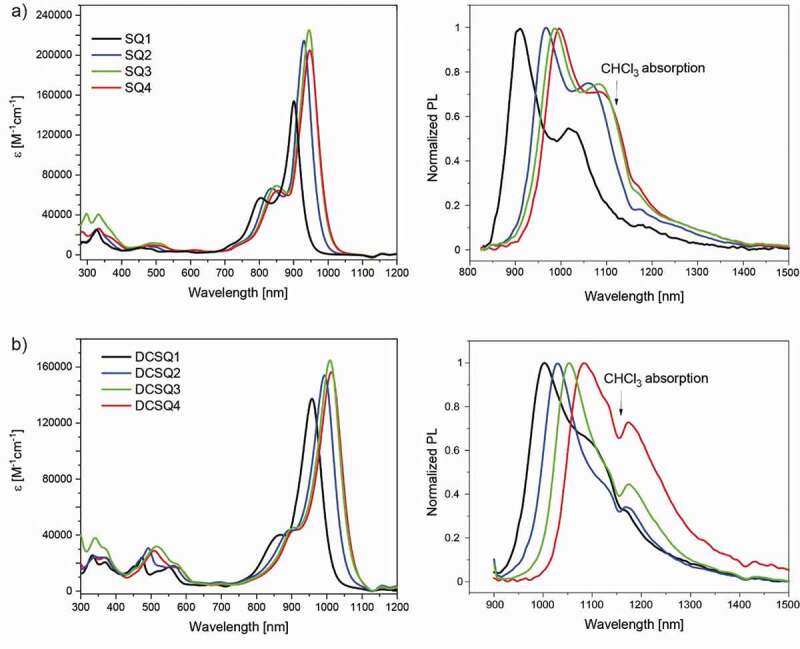
(a) Absorption (left) and fluorescence (right) spectra of squaraines, and (b) dicyanomethylene-substituted squaraines. Absorption spectra were measured in toluene, fluorescence spectra in chloroform

Absorption spectra of **SQ1** – **SQ4** in toluene solution are shown in Figure 1. The narrow absorption peaks with vibronic shoulders are characteristic for squaraines [45]. The absorption maxima increased from 900 nm for **SQ1** to 948 nm for **SQ4** (Table 1). This indicates that the aromatic substituents enlarge the π-conjugated system, which is confirmed by quantum chemical calculations of the electron density distributions in the frontier molecular orbitals (Figure S4). Typical for squaraine dyes are the very high (> 150000 M^−1^cm^−1^) molar absorption coefficients of the main S_0_ → S_1_ (HOMO → LUMO) optical transition. This band shows a negative solvatochromism and, for example for **SQ4**, the wavelength of the absorption maximum is at 920 nm in ethanol, compared to 935 nm in chloroform or 928 nm in acetonitrile (Figure S5). The hypsochromic shift with increasing solvent polarity indicates a relatively more polar ground state and is well-known for squaraine dyes [46,47]. The absorption spectra show an additional weak absorption at around 500 nm, as well as a band between 300 nm and 400 nm that is attributed to the absorption of the (substituted) benz[*cd*]indole moieties [48].

**Table 1.** Absorption and fluorescence data for squaraines and dicyanomethylene-substituted squaraines

	λ_max,sol_ ^[a]^	ε ^[a]^	S1 (calc.) ^[b,c]^	S2 (calc.)^[b,d]^	λ_max,film_ ^[e]^	λ_em_ ^[f]^	Δ_Stokes_
	[nm]	[M^−1^cm^−1^]	[nm], (f)	[nm], (f)	[nm]	[nm]	[cm^−1^]
SQ1	900 (880)	152 000	766 (1.67)	421 (0)	966	909	363
SQ2	930 (917)	214 800	793 (1.96)	422 (0)	1007	967	564
SQ3	944 (932)	225 500	801 (2.19)	426 (0)	1020	986	588
SQ4	948 (935)	204 800	807 (2.01)	430 (2x10^−4^)	1024	996	655
DCSQ1	958 (935)	137 000	796 (1.17)	430 (0.44)	995	1002	715
DCSQ2	994 (971)	154 100	823 (1.34)	440 (0.59)	1053	1029	581
DCSQ3	1008 (987)	164 800	832 (1.45)	444 (0.80)	1063	1053	635
DCSQ4	1014 (991)	156 400	840 (1.38)	448 (0.69)	1067	1084	866

[a] measured in toluene. In bracket absorption maxima in chloroform. [b] prominent calculated excited states, oscillator strength (f) in bracket. DFT methods: Geometry optimization at BLYP35/def2-TZVPP in toluene (PCM); Excitation wavelength: M06-2X/def2-TZVPP in toluene [54]. [c] HOMO → LUMO. [d] HOMO → LUMO+1. The calculated oscillator strength of S2-S4 for SQ1 – SQ4 is close to zero. [e] spin coated from chloroform solution onto a glass substrate. [f] measured in chloroform.

The dicyanomethylene acceptor group induced a substantial redshift of the absorption maxima, and the corresponding dyes absorb between 958 nm (**DCSQ1**) and 1014 nm (**DCSQ4**), see Table 1. To our knowledge, **DCSQ3** and **DCSQ4** are the first squaraine dyes with an absorption maximum beyond 1000 nm. The bathochromic shift of the dicyanomethylene-substituted squaraines comes along with a decrease of the experimental molar absorption coefficient at λ_max_, also confirmed by the calculated oscillator strengths for the SQ and DCSQ dyes (Table 1). The additional hypsochromic absorption band in the 450 nm – 600 nm wavelength range for the DCSQ dyes (molecular symmetry C*_2v_*) can be assigned to the HOMO → LUMO+1 transition; this transition is symmetry forbidden for the SQ dyes (*C_2h_* symmetry) and therefore very weak (Table 1, Figure 1a) [37,49].

Figure 1 also shows the fluorescence spectra of the dyes. The Stokes’ shifts are larger for the DCSQ dyes with a discernible trend that the shifts increase with the π-conjugation and donor strength of the aromatic substituents for both dye families. An increase of the Stokes’ shift indicates a more pronounced change of the molecular structure in the excited state. Experimental constraints have prevented to determine the fluorescence quantum yields, which are <0.05% for **DCSQ1** and **DCSQ4**. A low fluorescence quantum yield in our case is likely due to the exponential increase of non-radiative losses for molecules with smaller energy gap [50]. It has been shown that squaraines can undergo *trans-cis* photoisomerization via a twisted intramolecular charge transfer state, a reaction that provides a nonemissive decay channel of the excited state. However, this process is inhibited if rotations are hindered and dyes are conformationally locked, as it applies to our dyes [51,52].

**Figure 2.**
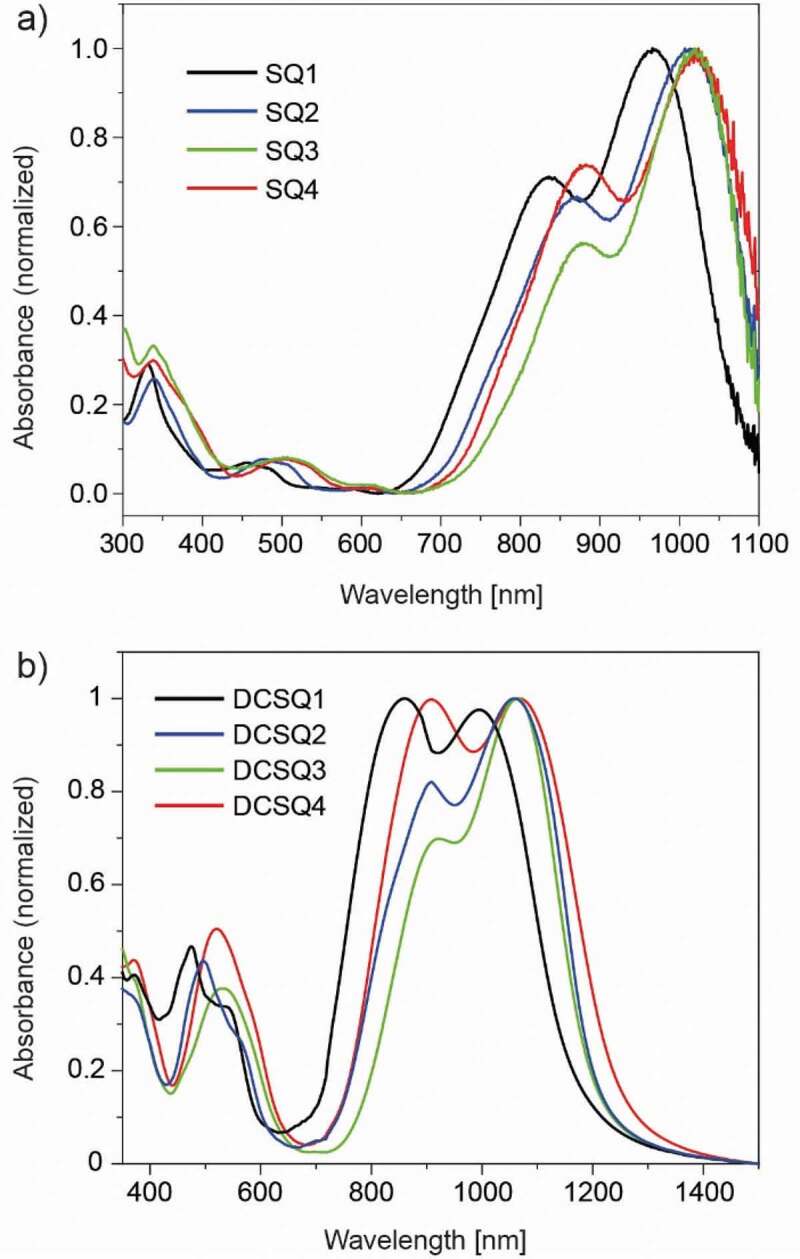
Absorbance spectra of (a) SQ and (b) DCSQ dye films coated from chloroform on glass

The absorbance spectra of spin coated films (Figure 2) were considerably broadened and the maxima were red-shifted (by 60–90 nm) compared to the solution spectra. This can be explained with strong intermolecular interactions and increased molecular ordering in the solid state. For **DCSQ2** and **DCSQ3**, a broad dimer peak covering the vibrational band appeared at shorter wavelength. The pronounced attenuance feature for **DCSQ1** (at 854 nm) and **DCSQ4** (at 908 nm) is characteristic for H-aggregates, suggesting that these dyes self-organize during film formation [53]. Dye aggregation in the film is disrupted when blended with the fullerene derivative PCBM ([6,6]-phenyl-C_61_-butyric acid methyl ester), see below.

**Figure 3.**
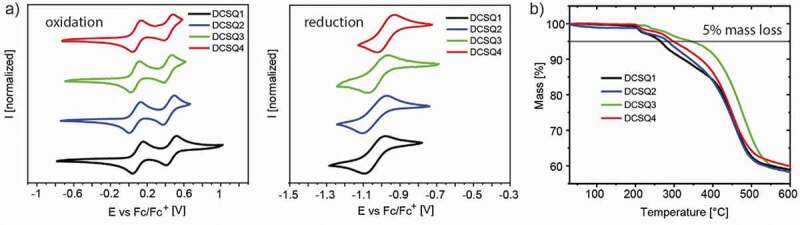
(a) Cyclic voltammograms, and (b) thermal gravimetric analysis of DCSQ dyes

The cyclic voltammograms and thermal gravimetric analysis graphs for the DCSQ dyes are shown in Figure 3, the corresponding data for the SQ dyes are compiled in Figure S6 and S7. In the cyclic voltammograms, all dyes showed two reversible one-electron oxidations and one reversible one-electron reduction. Elongation of the π-system at the benz[*cd*]indol substituents resulted in a narrowing of the electrochemical gap in each series. Assuming that the half-wave oxidation and reduction potentials correspond to the HOMO and LUMO levels and with an energy level of −5.1 eV vs. vacuum for the ferrocene/ferrocenium redox couple, the redox levels vs. vacuum can be calculated (Table 2). Introduction of the dicyanomethylene acceptor group decreased both the HOMO and LUMO levels, while the second half wave oxidation potential was hardly influenced. The optical band gaps from the onset absorption edge (from Figure 1) differ from the electrochemical band gaps by around +0.1 eV for the SQ dyes, and by around +0.7 eV for the DCSQ family [55].

**Table 2.** Electrochemical and thermal gravimetric analysis data of SQ and DCSQ dyes

	E_½,ox1_ ^[a]^	E_½,ox2_	E_½,red1_	E_gap,cv_	E_HOMO_ ^[b]^	E_LUMO_ ^[b]^	T_d_ ^[c]^
	[V]	[V]	[V]	[eV]	[eV]	[eV]	[^o^C]
SQ1	0.04	0.48	−1.14	1.18	−5.14	−3.96	198/257
SQ2	0.01	0.42	−1.12	1.13	−5.11	−3.98	222/294
SQ3	−0.02	0.40	−1.15	1.13	−5.08	−3.95	206/364
SQ4	−0.01	0.44	−1.12	1.11	−5.09	−3.98	200/305
DCSQ1	0.10	0.46	−1.04	1.14	−5.20	−4.06	203/265
DCSQ2	0.07	0.43	−1.04	1.11	−5.17	−4.06	200/284
DCSQ3	0.07	0.42	−1.02	1.09	−5.17	−4.08	229/344
DCSQ4	0.09	0.43	−0.98	1.07	−5.19	−4.12	205/297

[a] Measured in dichloromethane, potential against Fc/Fc^+^. [b] using −5.1 eV for Fc/Fc^+^ against vacuum [57]. [c] onset decomposition temperature/temperature at 5% mass loss.

Thermal gravimetric analysis under nitrogen atmosphere showed that both dye classes are stable up to 200 °C. Onset decomposition temperatures of around 200 °C have been reported for related squaraine dyes with different donor substituents [47,56], pointing to the lability of the central four-membered ring. As a reference point also the often cited temperatures at 5% mass loss are included in Table 2. It is clear that these values scale with the molecular weights of the corresponding dyes and therefore overestimate the thermal stability of the higher molecular weight squaraine dyes. Details of the thermal analysis, including Differential Scanning Calorimetry (DSC) data, are discussed in Figure S8.

### 2.3. SWIR upconversion devices

**Figure 4.**
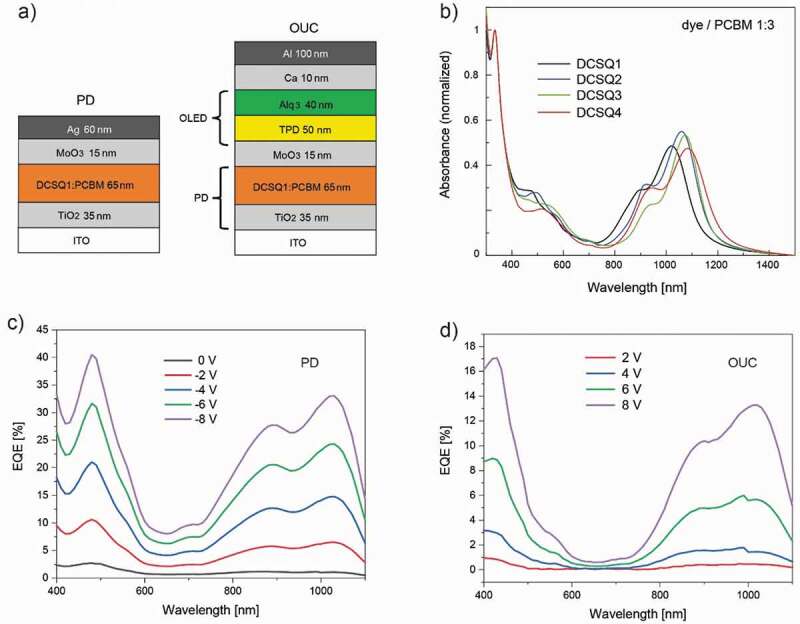
(a) Architecture of the photodetector (PD) and the upconversion device (OUC). (b) Absorbance spectra of DCSQ:PCBM blend films coated on glass. (c) EQE of the PD as function of voltage bias. (d) EQE of the OUC as function of voltage bias

The OUC consisted of a SWIR-sensitive PD and an OLED, stacked in series (Figure 4a). As SWIR absorber for the PD part, we chose **DCSQ1** from the DCSQ dye family and charges were photogenerated using a dye donor/PCBM acceptor heterojunction. From the absorbance spectra shown in Figure 4(b), it can be seen that dye aggregation in the blend films is suppressed for all DCSQ dyes, as opposed to the pure dye films shown in Figure 2(b). OUC devices were completed by combining the PD with a fluorescent tris(8-hydroxyquinolinato)aluminium (Alq_3_)-based OLED.

The functionality of the OUC can be explained as follows: both in the dark (off-state) and in the presence of NIR/SWIR light (on-state), a voltage bias is applied to the device. In the off-state, holes are blocked at the ITO/TiO_2_ interface and electrons are blocked at the *N,N’*-bis(3-methylphenyl)-*N,N’*-diphenylbenzidine (TPD)/Alq_3_ interface. Therefore, in the dark, no current is flowing and no visible light is emitted. With increasing voltage bias, a rising dark current can result in an undesirable dark current-induced luminance. In the on-state, light is absorbed in the PD unit and free charges are photogenerated. Electrons are extracted via the TiO_2_ anode and holes are driven via the hole-transporting MoO_3_ layer in the OLED where they recombine with electrons from the cathode under the emission of green light.

Figure 4(c) shows EQE spectra of the PD for different bias voltages, limited to a cutoff wavelength of 1100 nm by our instrument. The active layer thickness was around 65 nm, optimized in terms of a low dark current and high EQE value (Figure S9). We ascribe the apparent EQE peak at 480 nm to an optical interference effect (weak microcavity) because the absorbing layer is sandwiched between a weakly (glass/ITO) and strongly (Ag) reflecting interface (Figure S9) [58].

In the NIR/SWIR spectral range, the EQE of the PD followed the film absorbance spectrum. The EQE increased linearly with the applied voltage and reached a value of 33% at 1025 nm for −8 V. The reproducibility of device fabrication is demonstrated in Figure S10. Figure 4(d) shows the corresponding EQE spectra for the OUC. Again, the EQE followed the film absorbance spectrum, which confirms that the device is sensitive in the SWIR range, out to a wavelength of around 1200 nm (Figure 4b). For the OUC, the EQE increased superlinear and reached a value of 13.3% for 8 V. When evaluating the EQE dependence on the electric field for the two devices, we found that values matched for high fields, but the EQE of the OUC dropped below the linear trend of the PD for lower fields. We ascribe this to small energetic barriers for carrier transport in the OUC that can be effectively overcome when the electric field is increased.

**Figure 5.**
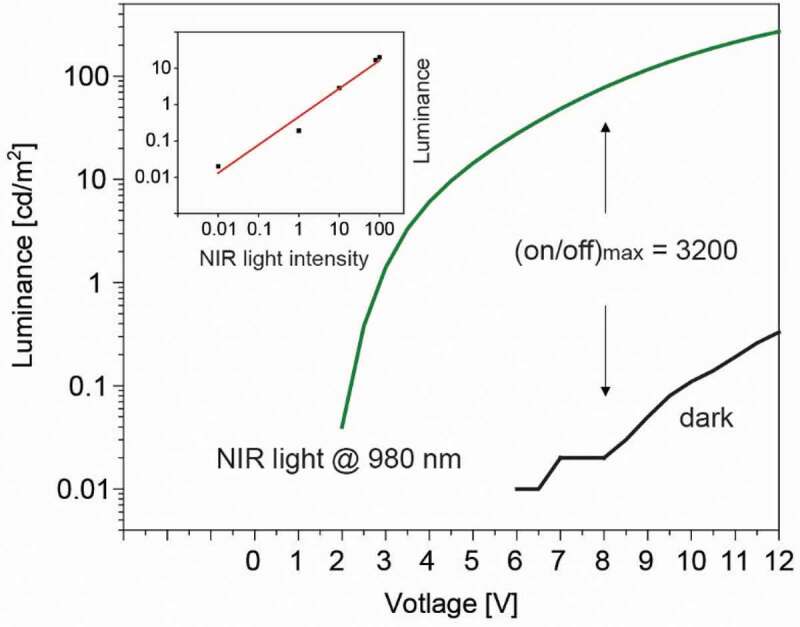
Luminance vs voltage trend of the OUC with and without NIR light. The inset shows the response of the NIR light-induced luminance when varying the NIR light intensity (from 40 to 0.4 mW cm^−2^), evaluated at a voltage of 8 V

The luminance vs voltage trend of the OUC is shown in Figure 5. In the dark, the luminance stayed below the detection limit of our setup (10^−2^ cd m^−2^) up to 6 V, and the dark luminance increased to a small value of 0.3 cd m^−2^ at 12 V. A low dark luminance is an important performance metrics of an OUC. Under ambient light conditions, a dark luminance level of below 10^−2^ cd m^−2^ is hardly detectable by the human eye. Therefore, even a small SWIR light-induced luminance results in a high image contrast that can visually be clearly differentiated against the (black) background.

We quantified the OUC on-state by using a light source at 980 nm. Device turn-on was at a low voltage of 2 V. The luminance increased with increasing voltage bias and reached, for example, 27 cd m^−2^ at 6 V, 76 cd m^−2^ at 8 V, or 260 cd m^−2^ at 12 V. The reproducibility of device fabrication is demonstrated in Figure S10. During light illumination, the luminance is limited by the number of hole charge carriers that are photogenerated in the PD and that are injected into the OLED. In our case, we do not observe a saturation of the luminance at higher voltage, because the EQE of the PD steadily increases when the voltage is increased. The luminance on-off ratio was evaluated in the voltage range where the dark luminance could be measured. Between 6 V and 12 V, the ratio peaked at a value of 3200 at 8 V. Note that the actual value of the on-off ratio depends on the NIR light intensity. If the light intensity is increased, more charges are generated and consequently the luminance increases. The inset of Figure 5 shows the linear response of the luminance when the NIR light intensity was varied over two orders of magnitude. A linear device response is beneficial for a direct imaging OUC. These results indicate that high-contrast images can be obtained by using a simple low-voltage battery to drive such OUCs.

The photon-to-photon conversion efficiency (P2PCE) describes the ratio between the number of visible photons emitted to the number of incident NIR photons. For the data shown in Figure 5, P2PCE increased with bias voltage and reached a value of 0.1% at 8 V and 0.3% at 12 V [38]. This low value is clearly limited by the EQE (~1% [59]) of the OLED. The P2PCE ≈ EQE(PD) x EQE(OLED) can be approximated from the individual EQEs of the PD and OLED. The EQE of the PD part was ~12% at an electric field of 8 V/active layer thickness. Therefore, the value of the approximated P2PCE ≈ 0.12 × 0.01 = 0.12% is expected to be in the range of 0.1%, in agreement with the experimental results.

Initial OUC stability tests are promising. As a qualitative statement, we found that the device performance is stable over a period of several weeks, when stored under inert conditions between subsequent measurements. We also stressed devices under constant NIR light illumination and voltage bias over a period of 1 day and found that the luminance output was constant (Figure S11).

## 3. Conclusions

We synthesized a series of squaraines with the aim to obtain dyes with selective light absorption extending considerably into the SWIR wavelength range. We demonstrated efficient SWIR-sensitive PDs and OUCs. In general, OUCs can be fabricated using low-cost manufacturing processes on large-area flexible substrates and can be operated at room temperature. Therefore, it is anticipated that OUCs can provide an interesting alternative to the existing SWIR imaging technology for novel consumer and low-end applications. In ongoing synthetic work, we are trying to shift the dye absorption further into the SWIR range by increasing the donor strength and using different acceptors on the squaric acid core. A major advantage of narrowband polymethine dyes compared to colloidal quantum dot absorbers is that light absorption in the visible is small, resulting in upconverters with selective SWIR response. For squaraines with extended absorption into the SWIR, it is fair to mention a potential tradeoff between a further bathochromic shift and SWIR selectivity. As we found for the DCSQ family, bulky substitution at the acceptor unit locks the dyes in the cis conformation, resulting in allowed optical transitions in the visible. Therefore, the synthetic challenge lies in the design of next-generation SWIR squaraine dyes with a most stable *trans* conformation.

## Supplementary Material

Supplemental Material
