# Supplementary material for: Shortwave infrared-absorbing squaraine dyes for all-organic optical upconversion devices
Source: Sci Technol Adv Mater. 2021 Apr 13;22(1):194–204. doi: 10.1080/14686996.2021.1891842 (PMC8049466; doi:10.1080/14686996.2021.1891842)
Supplement: Supplemental Material [file TSTA_A_1891842_SM5734.docx]

SUPPORTING INFORMATION

**Shortwave infrared-absorbing squaraine dyes for**

**all-organic optical upconversion devices**

Karen Strassel, Wei-Hsu Hu, Sonja Osbild, Daniele Padula, Daniel Rentsch, Sergii Yakunin, Yevhen Shynkarenko, Maksym Kovalenko, Frank Nüesch, Roland Hany, Michael Bauer

*Correspondence should be addressed to: Dr. Roland Hany

Empa / Laboratory for Functional Polymers

CH-8600 Dübendorf - Switzerland

Phone +41 58 765 4084

E-mail [roland.hany@empa.ch](mailto:roland.hany@empa.ch)

**Table S1.** Representative list of organic dyes with selective absorption maxima beyond 1000 nm.

|  | | | | | | | | |
| --- | --- | --- | --- | --- | --- | --- | --- | --- |
| **Name in Ref.** | **R** | **X** | **Y^-^** | **n** | **m** | **A** | **λ_max_ [nm]**  **(solvent)** | **Ref** |
| IR-1048 | Cl | Cl | BF_4_^-^ | 2 | 4 | H | 1048 (EtOH) | ^1^ |
| S 0734 | Cl | H | BF_4_^-^ | 2 | 4 | H | 1014 (MeOH) | ^2^ |
| S 0813 | Ph | H | BF_4_^-^ | 1 | 4 | H | 1024 (MeOH) | ^3^ |
| S 2544 | Ph | H | ArSO_3_^-^ | 1 | 4 | H | 1024 (MeOH) | ^4^ |
| FD-1080 | Cl | H | Na^+^ | 2 | 4 | SO_3_^-^ | 1064 (PBS) | ^5^ |
| 5a | Cl | H | ClO_4_^-^ | 1 | 8 | H | 1059 (CH_2_Cl_2_) | ^6^ |
| 5b | Cl | H | (C_6_F_5_)_4_B^-^ | 1 | 8 | H | 1060 (CH_2_Cl_2_) | ^6^ |
| 5c | 4-Me-C_6_H_4_O | H | (C_6_F_5_)_4_B^-^ | 1 | 8 | H | 1014 (CH_2_Cl_2_) | ^6^ |
| 5d | 4-Me-C_6_H_4_S | H | (C_6_F_5_)_4_B^-^ | 1 | 8 | H | 1068 (CH_2_Cl_2_) | ^6^ |
| 6 | Cl | H | (C_6_F_5_)_4_B^-^ | 2 | 8 | H | 1033 (CH_2_Cl_2_) | ^6^ |

|  | | | | |
| --- | --- | --- | --- | --- |
| **Name in Ref.** | **Pos.** | **R** | **λ_max_ [nm]**  **(solvent)** | **Ref** |
| Flav7 | 7 | NMe_2_ | 1027 (CH_2_Cl_2_) | ^7,8^ |
| 5-Flav7 | 5 | NMe_2_ | 1004 (CH_2_Cl_2_) | ^8^ |
| 6-Flav7 | 6 | NMe_2_ | 1048 (CH_2_Cl_2_) | ^8^ |

| **Name in Ref.** | **Structure** | | **λ_max_ [nm]**  **(solvent)** | **Ref** |
| --- | --- | --- | --- | --- |
| IR-1061 |  | | 1061 (CH_2_Cl_2_) | ^9^ |
| Various similar modified chalcogenopyrylium-terminated heptamethines can be found in literature | | | | ^10–15^ |
| BTC1070 |  | | 1014 (CH_2_Cl_2_) | ^16^ |
| 5L5 |  | R = Cl | >1100 (MeCN) | ^17^ |
| 5H5 |  | R = H | 1069 (MeCN) | ^17^ |
| PD 2716 |  | | 1085 (MeCN) | ^18^ |
| PD 2892 |  | | 1196 (MeCN) | ^18^ |
| JB17-08 |  | | 1043 (MeCN) | ^19^ |

| **Name in Ref.** | **Structure** | **λ_max_ [nm]**  **(solvent)** | **Remarks** | **Ref** |
| --- | --- | --- | --- | --- |
| 1a |  | Approx. 1100 (DCE) | Low yield | ^20^ |
| 3d |  | 1081  (CH_2_Cl_2_) |  | ^21^ |

|  | | | |
| --- | --- | --- | --- |
| **R'** | **λ_max_ [nm]**  **(solvent)** | **Remarks**  R = 2,6-diisopropylphenyl | **Ref** |
| H | 1018 (CH_2_Cl_2_) | Additional strong peak at approx. 560 nm, low solubility, no NMR/purification | ^22^ |
| 4-(t-octyl)phenoxy | 1037 (CH_2_Cl_2_) | Additional strong peak at 567 nm | ^22^ |

|  | | | | | | | |  |  |
| --- | --- | --- | --- | --- | --- | --- | --- | --- | --- |
| **Name in Ref.** | | **λ_max_ [nm]**  **(solvent)** | | **Remarks** | | **Ref** | | |  |
| 6 | | 1084 (tol) | | CT-dye (low extinction coefficient) | | ^23^ | | |  |
|  | | | | | | | | | |
| **Name in Ref.** |  | | **λ_max_ [nm]**  **(solvent)** | | **Remarks** | | **Ref** | | |
| 8 | X = Se, Y =S | | 1036 (tol) | | CT-dye (low extinction coefficient) | | ^23^ | | |
| 9 | X = Y = Se | | 1177 (tol) | | CT-dye (low extinction coefficient) | | ^23^ | | |

**References Table S1**

1 IR 1048 Sigma Aldrich.

2 S 0743 FEW Chemicals.

3 S 0813 FEW Chemicals.

4 S 2544 FEW Chemicals.

5 B. Li, L. Lu, M. Zhao, Z. Lei and F. Zhang, *Angew. Chemie Int. Ed.*, 2018, **57**, 7483.

6 K. Funabiki, R. Yanagawa, Y. Kubota and T. Inuzuka, *New J. Chem.*, 2019, **43**, 7491.

7 E. D. Cosco, J. R. Caram, O. T. Bruns, D. Franke, R. A. Day, E. P. Farr, M. G. Bawendi and E. M. Sletten, *Angew. Chem. Int. Ed.*, 2017*,* **56***,* 13136*.*

8 M. Pengshung, J. Li, F. Mukadum, S. A. Lopez and E. M. Sletten, *Org. Lett.*, 2020, **22**, 6150.

9 IR-1061 Sigma Aldrich.

10 A. Scarpaci, A. Nantalaksakul, J. M. Hales, J. D. Matichak, S. Barlow, M. Rumi, J. W. Perry and S. R. Marder, *Chem. Mater.*, 2012, **24**, 1606.

11 S. Barlow, J.-L. Brédas, Y. A. Getmanenko, R. L. Gieseking, J. M. Hales, H. Kim, S. R. Marder, J. W. Perry, C. Risko and Y. Zhang, *Mater. Horiz.*, 2014, **1**, 577.

12 I. Davydenko, S. Benis, S. B. Shiring, J. Simon, R. Sharma, T. G. Allen, S.-H. Chi, Q. Zhang, Y. A. Getmanenko, T. C. Parker, J. W. Perry, J.-L. Brédas, D. J. Hagan, E. W. Van Stryland, S. Barlow and S. R. Marder, *J. Mater. Chem. C*, 2018, **6**, 3613.

13 A. D. Kachkovski, M. A. Kudinova, N. A. Derevjanko and A. I. Tolmachev, *Dyes and Pigments*, 1991, **16**, 137.

14 J. B. Jarman and D. A. Dougherty, *Chem. Commun.*, 2019, **55**, 5511.

15 M. A. Kudinova, A. D. Kachkovski, V. V. Kurdyukov and A. I. Tolmachev, *Dyes and Pigments*, 2000, **45**, 1.

16 S. Wang, Y. Fan, D. Li, C. Sun, Z. Lei, L. Lu, T. Wang and F. Zhang, *Nat. Commun.*, 2019, **10**, 1058.

17 B. Ding, Y. Xiao, H. Zhou, X. Zhang, C. Qu, F. Xu, Z. Deng, Z. Cheng and X. Hong, *J. Med. Chem.*, 2019, **62**, 2049.

18 S. Webster, L. A. Padilha, H. Hu, O. V. Przhonska, D. J. Hagan, E. W. Van Stryland, M. V. Bondar, I. G. Davydenko, Y. L. Slominsky and A. D. Kachkovski, *J. Lumin.*, 2008, **128**, 1927.

19 H. Hu, O. V. Przhonska, F. Terenziani, A. Painelli, D. Fishman, T. R. Ensley, M. Reichert, S. Webster, J. L. Bricks, A. D. Kachkovski, D. J. Hagan and E. W. Van Stryland, *Phys. Chem. Chem. Phys.*, 2013, **15**, 7666.

20 M. Tian, S. Tatsuura, M. Furuki, Y. Sato, I. Iwasa and L. S. Pu, *J. Am. Chem. Soc.*, 2003, **125**, 348.

21 T. P. Simard, J. H. Yu, J. M. Zebrowski-Young, N. F. Haley and M. R. Detty, *J. Org. Chem.*, 2000, **65**, 2236.

22 Y. Avlasevich and K. Müllen, *Chem. Commun.*, 2006, **9**, 4440.

23 G. Qian, B. Dai, M. Luo, D. Yu, J. Zhan, Z. Zhang, D. Ma and Z. Y. Wang, *Chem. Mater.*, 2008, **20**, 6208.

**EXPERIMENTAL**

**References for the Experimental Part**

S1 C. M. Cardona, W. Li, A. E. Kaifer, D. Stockdale and G. C. Bazan, *Adv. Mater.*, 2011, **23**, 23671.

S2 M. J. Frisch, G. W. Trucks and H. B. Schlegel, et al. Gaussian16, Revision B.01.**2016**.

S3 K. Strassel, A. Kaiser, S. Jenatsch, A. C. Véron, S. B. Anantharaman, E. Hack, M. Diethelm, F. Nüesch, R. Aderne, C. Legnani, S. Yakunin, M. Cremona and R. Hany, *ACS Appl. Mater. Interfaces*, 2018, **10**, 11063.

**Methods**

Chemicals and solvents were purchased from commercial sources (Sigma Aldrich, Merck, VWR, TCI and abcr) and were used without further purification. Reactions were carried out under argon atmosphere. Column chromatography was done using silica gel (pore size 40-63 μm, Normasil 60® from VWR chemicals).

^1^H and ^13^C NMR data were recorded at 400.2 MHz and 100.6 MHz using a 5 mm CryoProbe™ Prodigy probe equipped with z-gradient on a Bruker Avance III 400 NMR spectrometer (Bruker Biospin AG, Fällanden, Switzerland). 1D ^1^H, ^13^C and ^1^H 1D-NOESY NMR experiments, as well as 2D-correlated ^1^H-^13^C HSQC, ^1^H-^13^C HMBC, and ^1^H-^1^H DQF-COSY experiments were performed at 298 K using the Bruker standard pulse programs and parameter sets applying 90° pulse lengths of 11.4 µs (^1^H) and 10.0 µs (^13^C). Chemical shifts (δ in ppm) are calibrated to residual solvent peaks (CDCl_3_ δ = 7.26 and 77.0 ppm; CD_2_Cl_2_ δ = 5.32 and 53.84 ppm; THF-d_8_ δ = 3.58 and 67.21 ppm). Coupling constants *J* are reported in Hz and for ^1^H NMR data coupling patterns are described as s = singlet, d = doublet, t = triplet, q = quartet, quint = quintet, m = multiplet, br = broad, and for ^13^C NMR data s = quaternary carbon, d = CH, t = CH_2_, and q = CH_3_.

Absorption spectra of solutions and thin films were recorded on a Varian Cary 50 Scan or a Shimadzu UV-VIS 3600 spectrophotometer. A cuvette filled with solvent or the glass substrate was used as the baseline. To obtain the extinction coefficient ε a stock solution was diluted to different concentrations. Plotting absorption vs. concentration and using a linear fitting were used to calculate the extinction coefficient ε. Fluorescence spectra were recorded from chloroform solutions of dyes with a Fluorolog-3 spectrometer (Horiba Jobin-Yvon) equipped with an 808 nm laser diode as excitation source and a liquid nitrogen-cooled InGaAs photodetector. For better signal-to-noise the laser was modulated with an opto-mechanical chopper and the fluorescence signal was amplified and restored with a lock-in amplifier (Standford Research SR510).

Cyclic voltammetry was measured on a computer-controlled μAutolab Type III potentiostat using a three electrode system (GC working electrode, Pt counter electrode and Ag/ AgCl reference electrode) in a 0.1 M solution of *n*Bu_4_NPF_6_ in dichloromethane. The scan rate was 100 mV/s. The potentials were internally referenced to the ferrocene/ferrocenium (Fc/Fc^+^) redox pair. A HOMO energy level of -5.1 eV vs. vacuum was assumed. ^S1^

High-resolution electrospray ionization mass spectra (HR-ESI-MS) were acquired on a Bruker Daltonics maXis ESI-QTOF at ETH Zürich, and at University Zürich on a QExactive MS with a heated ESI source (ThermoFisher Scientific, Germany) or on a timsTOF Pro TIMS-QTOF-MS instrument (Bruker Daltonics, Germany). Differential scanning calorimetry was recorded on a Perkin Elmer DSC 8000 or a DSC 7 with heating and cooling rates of 20 °C/min. Thermogravimetric analysis was performed on a Netzsch TG 209 F1 Iris at a heating rate of 20 °C/min. DSC and TGA were measured under N_2_ atmosphere.

Quantum chemical calculations were carried out with the Gaussian16 software on molecules with alkyl side chains represented by a methyl group.^S2^ Geometry optimizations were carried out at the DFT/BLYP35/def2-TZVPP level using the PCM model for toluene (and CHCl_3_ for **SQ1**) to include solvent effects. Populations of conformers were evaluated based on internal energy differences between different minima. The evaluation of absorption wavelengths and oscillator strengths was carried out at the TDDFT/M06-2X/def2-TZVPP level using the PCM model for toluene. Transitions were assigned based on the CI coefficients obtained in the TDDFT analyses.

Devices were fabricated following procedures described in ref. [S3]. **DCSQ1** and PCBM (1:3 w/w) were dissolved in anhydrous CHCl_3_. The thickness of the PD blend layer was adjusted by varying the concentrations of the solutions (17.5 mg (dye+PCBM) ml^-1^ for 65 nm, 20 mg ml^-1^ for 75 nm, 10 mg ml^-1^ for 45 nm). Solutions were filtered and spin coated at 4000 rpm for 60 s inside the glove box. Further layers (MoO_3_ (evaporation rate 0.1-0.2 Å s^-1^), TPD (0.2 Å s^-1^), Alq_3_ (0.1-0.4 Å s^-1^), Ca (0.1 Å s^-1^), Ag (0.1-0.2 Å s^-1^), Al (0.1-0.4 Å s^-1^)) were deposited via thermal evaporation at a pressure below 5 x 10^-6^ mbar.

Devices were characterized in a N_2_-filled air-tight box covered with a glass window. EQE spectra were measured on a commercial setup (SpeQuest, ReRa solutions BV). The monochromatic light was chopped at 85 Hz during the measurement without additional bias light. Optionally, a voltage bias (maximum -10 V allowed) was applied during spectral response measurements. J-V-L characteristics were measured with a Keithley 2400 and a Konica Minolta luminance meter LS-110 equipped with a close-up lens No. 110. A 980 nm wavelength laser with a maximum intensity of 49.4 mW cm^-2^ reaching the sample from Thorlabs (CPS980) was used as illumination source. The NIR-photon-to-visible-photon conversion efficiency was calculated as described in ref. [S3]. For the linearity of the device response and the stability test we used a 780 nm laser (CPS780S from Thorlabs) with an intensity of 41.1 mW cm^-2^.

**Synthesis**

1-Octyl-(1*H*)-benz[*cd*]indol-2-one (**2**) was synthesized as previously reported.^S3^

6-Bromo-1-octylbenzo[*cd*]indol-2(1*H*)-one (**3**). Compound **2** (5.00 g, 17.8 mmol, 1 eq) was dissolved in acetonitrile (150 ml). *N*-bromosuccinimide (3.16 g, 17.8 mmol, 1 eq) in acetonitrile (100 ml) was added dropwise at 0 °C in the dark and the reaction mixture was stirred at room temperature overnight. The solvent was removed under reduced pressure, the residue dissolved in ethyl acetate, washed with water, aqueous NaHCO_3_-solution and brine and dried over Na_2_SO_4_. The product was obtained after column chromatography (cyclohexane/ethyl acetate, 20:1) as a yellow powder (4.50 g, 12.5 mmol, 70%).

1-Octyl-6-phenylbenzo[*cd*]indol-2(1*H*)-one (**4**). Compound **3** (300 mg, 0.83 mmol, 1 eq) and phenylboronic acid (137 mg, 1.12 mmol, 1.35 eq) were weighed in a pressure resistant vial and toluene (8.3 ml) was added. The solution was degassed with argon for 15 min and heated to 100 °C. Pd(PPh_3_)_4_ (96 mg, 0.08 mmol, 0.1 eq) was added and the mixture heated to 100 °C for 20 min. A degassed solution of potassium carbonate (574 mg, 4.15 mmol, 5 eq) in a mixture of methanol (4.2 ml) and water (4.2 ml) was added and the reaction mixture was stirred at 100 °C for 12 h. The reaction was cooled to room temperature, water was added and the mixture was extracted with toluene. The organic phase was washed with 4% HCl and brine, dried over Na_2_SO_4_ and the solvent was removed under reduced pressure. The product was purified using column chromatography (cyclohexane/ethyl acetate 1:0 🡪 10:1) to give the product as yellow powder (237 mg, 0.66 mmol, 80%).

HR–MS (pos. ESI): m/z for [C25H28NO+]; calculated 358.2165, found 358.2164.

6-(9-Hexyl-9*H*-carbazol-2-yl)-1-octylbenzo[*cd*]indol-2(1*H*)-one (**5**). Compound **3** (380 mg, 1.05 mmol, 1 eq) and 9-Hexyl-2-(4,4,5,5-tetramethyl-1,3,2-dioxaborolan-2-yl)-9*H*-carbazole (475 mg, 1.26 mmol, 1.2 eq) were weighed in a pressure resistant vial and toluene (8.2 ml) was added. The solution was degassed with argon for 15 min and heated to 100 °C. Pd(PPh_3_)_4_ (122 mg, 0.11 mmol, 0.1 eq) was added and the mixture was heated to 100 °C for 20 min. A degassed solution of potassium carbonate (726 mg, 5.25 mmol, 5 eq) in a mixture of methanol (4.1 ml) and water (4.1 ml) was added and the reaction mixture was stirred at 100 °C for 12 h. The reaction was cooled to room temperature, water was added and the mixture extracted with toluene. The organic phase was washed with 4% HCl and brine, dried over Na_2_SO_4_ and the solvent removed under reduced pressure. The product was recrystallized from ethanol to give the product as yellow powder (435 mg, 0.82 mmol, 78%).

HR–MS (pos. ESI): m/z for [C37H43N2O+]; calculated 531.3370, found 531.3362.

1-Octyl-6-(thiophen-2-yl)benzo[*cd*]indol-2(1*H*)-one (**6**). Compound **3**, (500 mg, 1.39 mmol, 1 eq), K_2_CO_3_ (479 mg, 3.47 mmol, 2.5 eq) and Pd(OAc)_2_ (11 mg, 0.05 mmol, 0.035 eq) were weighed into a pressure resistant vial and put under nitrogen atmosphere. Anhydrous dimethylacetamide (8.2 ml) and degassed thiophene (1.67 ml, 1.75 g, 20.8 mmol, 15 eq) were added and the reaction mixture heated to 80 °C for 72 h. After cooling to room temperature, the mixture was diluted with toluene and washed with water, 4% HCl and brine, and was then dried over Na_2_SO_4_. The solvent was removed under reduced pressure and the residue purified by column chromatography using a mixture of cyclohexane and ethyl acetate (20:1 🡪 10:1). 353 mg (0.97 mmol, 70%) as a yellow powder was obtained.

HR–MS (pos. ESI): m/z for [C23H26NOS+]; calculated 364.1730, found 364.1731.

2-Methyl-1-octyl-benz[*cd*]indol-1-ium iodide (**7**) was synthesized as previously reported.^S3^

2-Methyl-1-octyl-6-phenylbenzo[*cd*]indol-1-ium iodide (**8**). Methylmagnesium chloride (1.2 ml, 3 M solution in THF, 3.52 mmol, 4 eq.) was added dropwise to a solution of **4** (310 mg, 0.88 mmol, 1 eq) in anhydrous THF (2 ml) at 55 °C. The reaction mixture was stirred at 55 °C for 3 h, cooled to room temperature and added under argon into an ice-water mixture (7 ml) containing concentrated HCl (0.4 ml). The remaining red solution was then added dropwise under argon to a solution of KI (0.34 g) in water (6 ml) at 0 °C. The red precipitate settled at the wall of the glass and was rinsed several times with water. The product (330 mg, 0.68 mmol, 77%) was used in the next reaction without further purification.

HR–MS (pos. ESI): m/z for [C26H30N+]; calculated 356.2373, found 356.2369.

6-(9-Hexyl-9*H*-carbazol-2-yl)-2-methyl-1-octylbenzo[*cd*]indol-1-ium iodide (**9**). Methylmagnesium chloride (1.4 ml, 3 M solution in THF, 4.28 mmol, 4 eq) was added dropwise to a solution of **5** (567 mg, 1.07 mmol, 1 eq) in anhydrous THF (2 ml) at 55 °C. The reaction mixture was stirred at 55 °C for 1 h, cooled to room temperature and added under argon into an ice-water mixture (6 ml) containing concentrated HCl (0.5 ml). A solution of KI (510 mg) in water (6 ml) was added to the remaining red solution under argon at 0 °C. The red precipitate settled at the wall of the glass and was rinsed several times with water. The product (560 mg, 0.85 mmol, 79%) was used in the next reaction without further purification.

HR–MS (pos. ESI): m/z for [C38H45N2+]; calculated 529.3577, found 529.3567.

2-Methyl-1-octyl-6-(thiophen-2-yl)benzo[*cd*]indol-1-ium iodide (**10**). Methylmagnesium chloride (1.10 ml, 3 M solution in THF, 3.32 mmol, 4 eq) was added dropwise to a solution of **6** (300 mg, 0.83 mmol, 1 eq) in anhydrous THF (2 ml) at 55 °C. The reaction mixture was stirred at 55 °C for 1 h, cooled to room temperature and added under argon into an ice-water mixture (7 ml) containing concentrated HCl (0.4 ml). The remaining red solution was then added dropwise under argon to a solution of KI (0.55 g) in water (6 ml) at 0 °C. The red precipitate settled at the wall of the glass and was rinsed 3 times with water. The product (300 mg, 0.61 mmol, 74%) was used in the next reaction without further purification.

HR–MS (pos. ESI): m/z for [C24H28NS+]; calculated 362.1937, found 362.1932.

**SQ1** was synthesized as previously reported.^S3^

HR–MS (pos. ESI): m/z for [C44H49N2O2+]; calculated 637.3789, found 637.3784.

**SQ2**. Compound **8** (317 mg, 0.66 mmol, 2 eq) and squaric acid (37 mg, 0.33 mmol, 1 eq) were dissolved in a mixture of toluene (3 ml) and *n*-butanol (4 ml). The solution was heated to reflux in a Dean-Stark apparatus for 4 hours. The solvent was removed under reduced pressure and the residue recrystallized from EtOH to yield **SQ2** as a dark purple powder (110 mg, 0.14 mmol, 42%).

HR–MS (pos. ESI): m/z for [C56H57N2O2+]; calculated 789.4415, found 789.4403.

**SQ3**. Compound **9** (300 mg, 0.46 mmol, 2 eq) and squaric acid (26 mg, 0.23 mmol, 1 eq) were dissolved in a mixture of toluene (2 ml) and *n*-butanol (3 ml). The solution was heated to reflux in a Dean-Stark apparatus for 4 hours. The solvent was removed under reduced pressure and the residue recrystallized from EtOH to yield **SQ3** as a dark purple powder (125 mg, 0.11 mmol, 48%).

HR–MS (pos. ESI): m/z for [C80H87N4O2+]; calculated 1135.6824, found 1135.6812.

**SQ4**. Compound **10** (303 mg, 0.62 mmol, 2 eq) and squaric acid (35 mg, 0.31 mmol, 1 eq) were dissolved in a mixture of toluene (2 ml) and *n*-butanol (3 ml). The reaction mixture was heated to reflux in a Dean-Stark apparatus for 5 hours. The solvent was removed under reduced pressure and the residue recrystallized from EtOH to yield **SQ4** as a dark purple powder (150 mg, 0.19 mmol, 61%).

HR–MS (pos. ESI): m/z for [C52H53N2O2S2+]; calculated 801.3543, found 801.3533.

**DCSQ1**. 2-Methyl-1-octylbenzo[*cd*]indol-1-ium iodide (**7**) (2.04 g, 5.01 mmol) was dissolved in 90 ml dry toluene and heated to 140 °C employing a water separator with 4 Å molecular sieve (MS). A solution of triethylammonium 2-butoxy-3-(dicyanomethylene)-4-oxocyclobut-1-en-1-olate (**11**) (800 mg, 2.50 mmol) in 90 ml *n*-butanol was slowly added to the solution. The reaction mixture was heated to 140 °C for 5 hours. The mixture was cooled down and the solvent was removed under reduced pressure. The crude product was dissolved in DCM and filtered through silica gel. The crude product was precipitated from DCM/*n*-heptane to afford the dye as a black solid (1.06 g, 1.54 mmol, 61%).

HR–MS (pos. ESI): m/z for [C47H49N4O+]; calculated 685.3901, found 685.3901.

**DCSQ2**. Triethylammonium 2-butoxy-3-(dicyanomethylene)-4-oxocyclobut-1-en-1-olate (**11**) (85 mg, 0.27 mmol) was dissolved in 50 ml *n*-butanol, 2,2-methyl-1-octyl-6-phenyl-benzo[*cd*]indol-1-ium iodide (**8**) (270 mg, 0.56 mmol) and 50 ml toluene were added and the mixture was heated to 130 °C employing a water separator with 4 Å MS for 2 hours. The mixture was cooled down and the solvent was removed under reduced pressure. The crude mixture was dissolved in DCM and filtered through silica gel. The crude product was precipitated from DCM/*n*-heptane to afford the product as a black solid (118 mg, 0.14 mmol, 53%).

HR–MS (pos. ESI): m/z for [C59H57N4O+]; calculated 837.4527, found 837.4519.

**DCSQ3**. Triethylammonium 2-butoxy-3-(dicyanomethylene)-4-oxocyclobut-1-en-1-olate (**11**) (75 mg, 0.23 mmol) was dissolved in 50 ml *n*-butanol, 6-(9-hexyl-9*H*-carbazol-2-yl)-2-methyl-1-octylbenzo[*cd*]indol-1-ium iodide (**9**) (324 mg, 0.49 mmol) and 50 ml toluene were added and the mixture was heated to 130 °C employing a water separator with 4 Å MS for 2 hours. The mixture was cooled down and the solvent was removed under reduced pressure. The mixture was dissolved in DCM and filtered through silica gel. The crude product was precipitated from DCM/*n*-heptane to afford the product as a black solid (140 mg, 0.12 mmol, 50%).

HR–MS (pos. ESI): m/z for [C83H87N6O+]; calculated 1183.6936, found 1183.6897.

**DCSQ4**. 2-Methyl-1-octyl-6-(thien-2-yl)benzo[*cd*]indol-1-ium iodide (**10**) (274 mg, 0.56 mmol) was dissolved in 90 ml dry toluene 45 ml *n*-butanol. A solution of triethylammonium 2-butoxy-3-(dicyanomethylene)-4-oxocyclobut-1-en-1-olate (**11**) (85 mg, 0.27 mmol) in 45 ml *n*-butanol was added and the mixture was heated to 130 °C employing a water separator with 4 Å MS for 4 hours. The mixture was cooled down and the solvent was removed under reduced pressure. The mixture was dissolved in dichloromethane and filtered through silica gel. The crude product was precipitated from DCM/*n*-heptane to afford the product as a black solid (102 mg, 0.12 mmol, 45%).

HR–MS (pos. ESI): m/z for [C55H53N4OS2+]; calculated 849.3655, found 849.3649.

**Isomers of squaraine dyes**


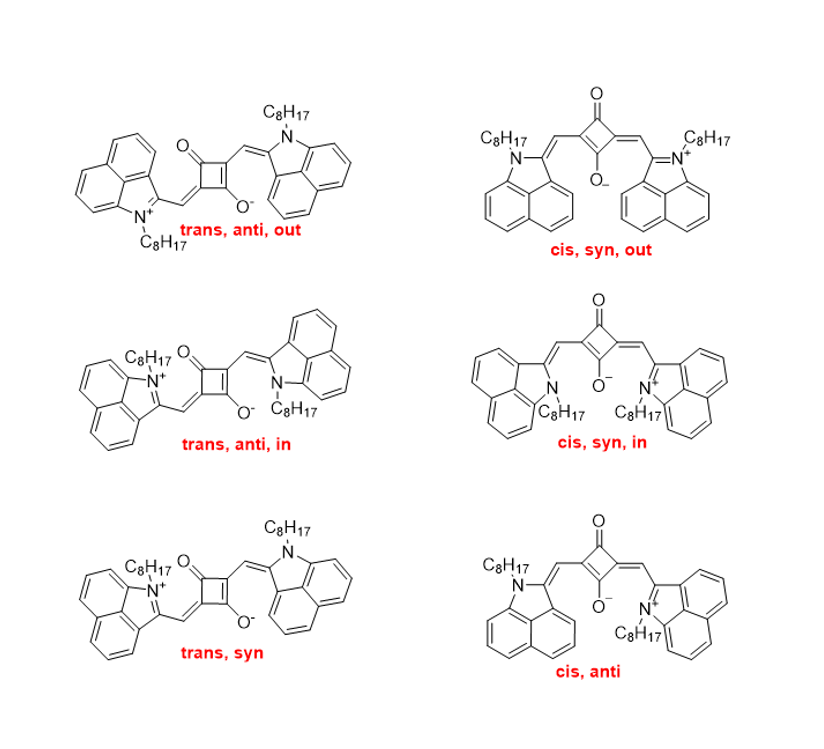


**Fig. S1.** Drawing of the six stereoisomers of **SQ1**.

| Isomer | Boltzmann population (%) |
| --- | --- |
| cis syn out | 12.70 (14.52) |
| cis syn in | 0.01 (0.01) |
| cis anti | 0.38 (0.37) |
| trans anti out | 84.39 (83.14) |
| trans anti in | 0.07 (0.05) |
| trans syn | 2.45 (1.91) |

Population of the different **SQ1** isomers in toluene (chloroform).

| Isomer | Population (%) **SQ4** | Population (%) **DCSQ4** |
| --- | --- | --- |
| cis syn out | 12.60 | 94.57 |
| cis syn in | 0.01 | 0.18 |
| cis anti | 0.45 | 5.20 |
| trans anti out | 84.36 | 0.04 |
| trans anti in | 0.07 | 0 |
| trans syn | 2.52 | 0 |

Population of the different isomers of **SQ4** and **DCSQ4**. For **DCSQ4**, the most stable isomer by far is cis-syn-out and no other isomers were detected with NMR spectroscopy.

Isomers of **SQ1** observed in the ^1^H-NMR-spectrum:

trans-anti-out

^1^H NMR (CDCl_3_, 400.2 MHz): *δ* 9.15 (s (br), 2H, H-9); 7.95 (d, *J* = 8.4, 2H, H-7); 7.85 (dd, *J* = 8,4, 7.7, 2H, H-8); 7.58 (m, 2H, H-3); 7.52 (dd, *J* = 7.7, 2H, H-2); 7.07 (m, 2H, H-1); 6.45 (s (br), 2H, H-20); 4.20 (m (br), 4H, H-12); 1.89 (m, 4H, H-13); 1.46 (m, 4H, H-14); 1.35 (m, 4H, H-15); 1.2-1.3 (m, 12H, H-16, 17, 18); 0.87 (t, *J* = 6.9, 6H, H-19).

cis-syn-out

^1^H NMR (CDCl_3_, 400.2 MHz): *δ* 8.84 (d, *J* = 7.3, 2H, H-9); 8.23 (d, *J* = 8.0, 2H, H-7); 7.97 (m, 2H, H-8); 7.83 (d, *J* = 8.1, 2H, H-3); 7.68 (dd, *J* = 8.1, 7.7, 2H, H-2); 7.56 (m, 2H, H-1); 6.22 (s, 2H, H-20); 4.44 (t, *J* = 7.3, 4H, H-12); 1.89 (m, 4H, H-13); 1.46 (m, 4H, H-14); 1.35 (m, 4H, H-15); 1.2-1.3 (m, 12H, H-16, 17, 18); 0.85 (m, 6H, H-19).


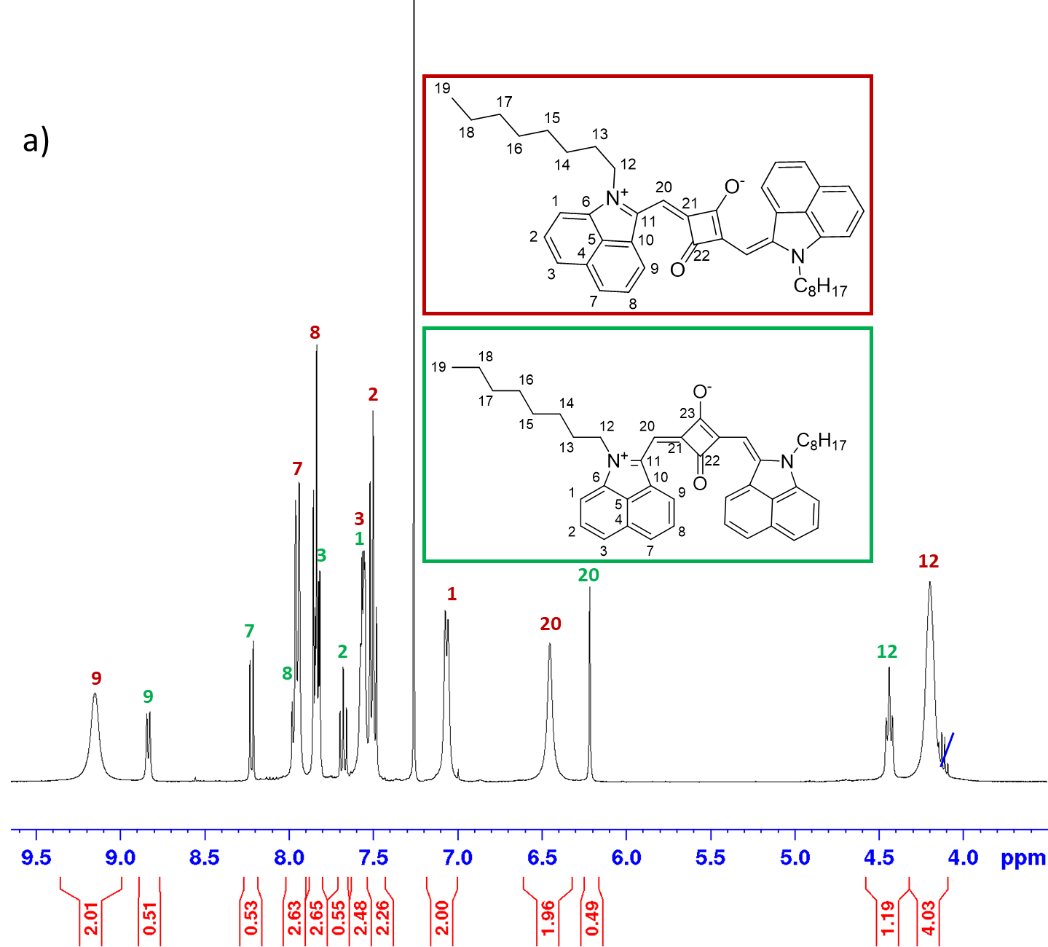


**
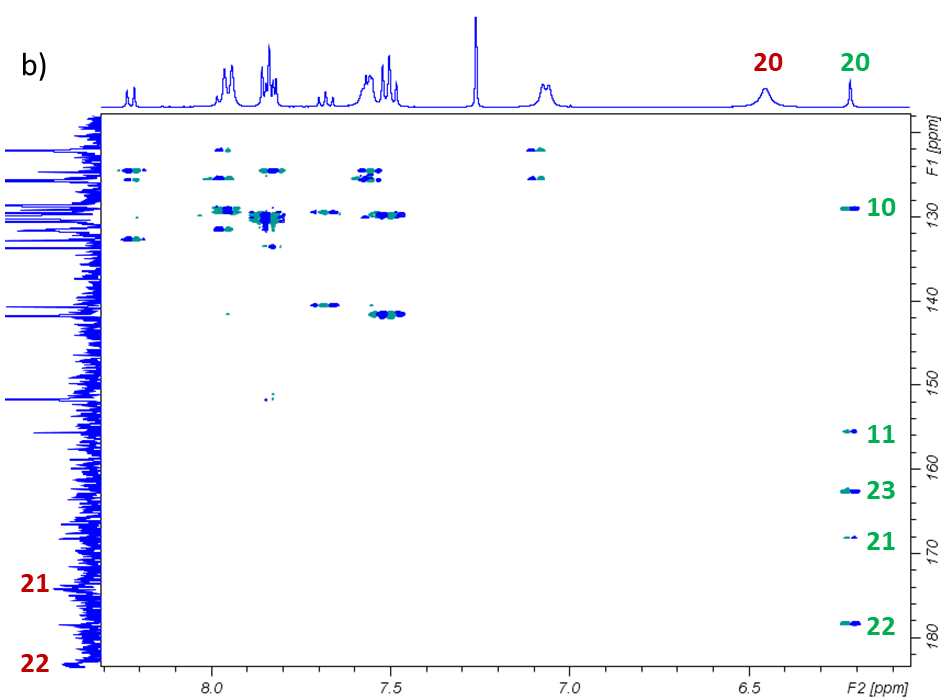
**

**Fig. S2.** Regions of interest of the a) ^1^H-NMR spectrum of a sample of **SQ1** in CDCl_3_ showing the two isomers trans-anti-out and cis-syn-out. b) HMBC spectrum of a sample of **SQ1** in CDCl_3_. For the minor isomer, H-20 (green) correlates the two carbonyl groups C-22 and C-23. This is an indication that the cis isomer is present, because the two carbonyl groups are identical in the trans isomer. For the trans isomer the corresponding cross peaks are not detectable. The ^1^H NMR resonances of the trans isomer are relatively broad, resulting in the relaxation of the magnetization already during the HMBC pulse sequence.

**
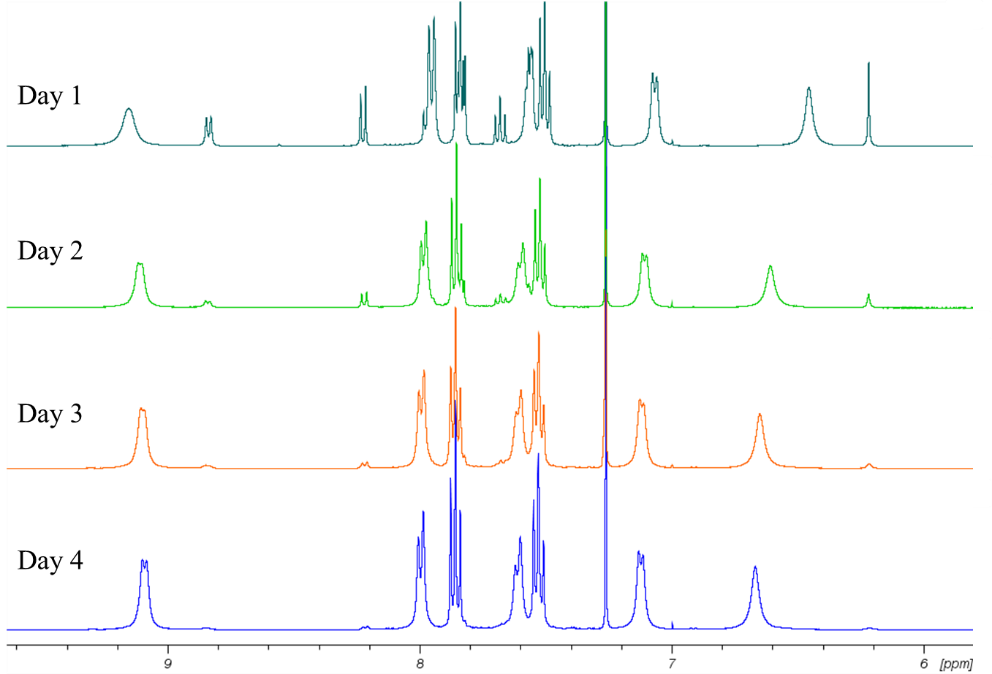
**

**Fig. S3.** ^1^H-NMR spectra of a sample of **SQ1** recorded over 4 days in CDCl_3_ showing the decrease of the resonances of the cis-syn-out isomer over time. The minor cis isomer was not, or only in very small amounts, observed in the raw mixture after the reaction (not shown here), it showed a tendency to form during purification, especially when crystallization was involved. Day 1 shows the NMR spectrum after recrystallization of the product. One can observe that if the sample is kept in solution, the minor isomer decreased over time to a steady state equilibrium of ≈5%; Day (amount of cis isomer): 1 (20%), 2 (10%), 3 (5%), 4 (3%).

**Frontier molecular orbitals**


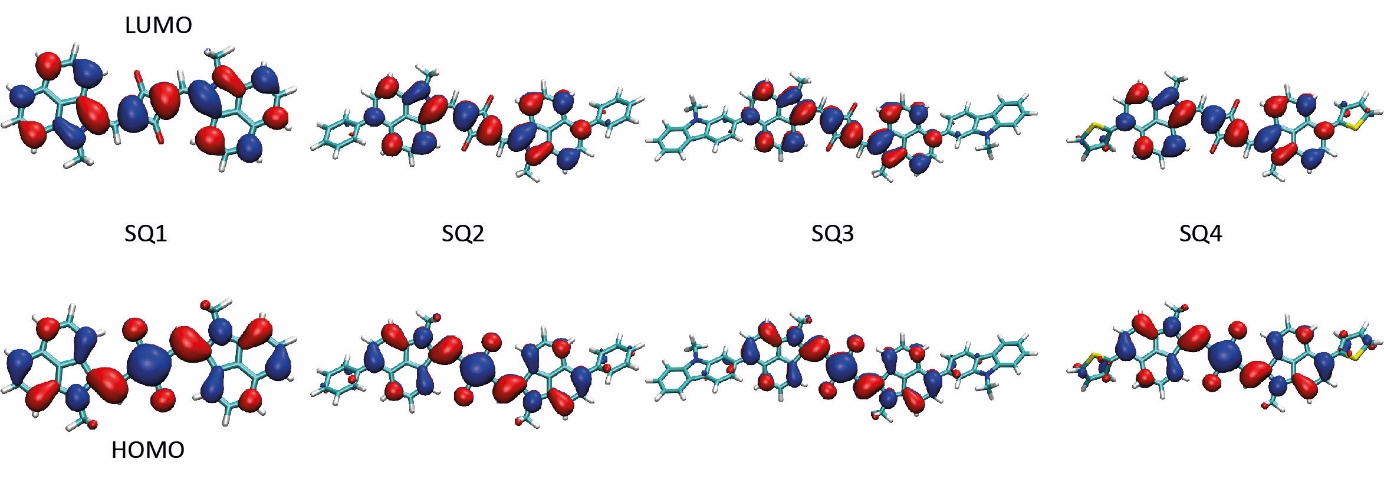


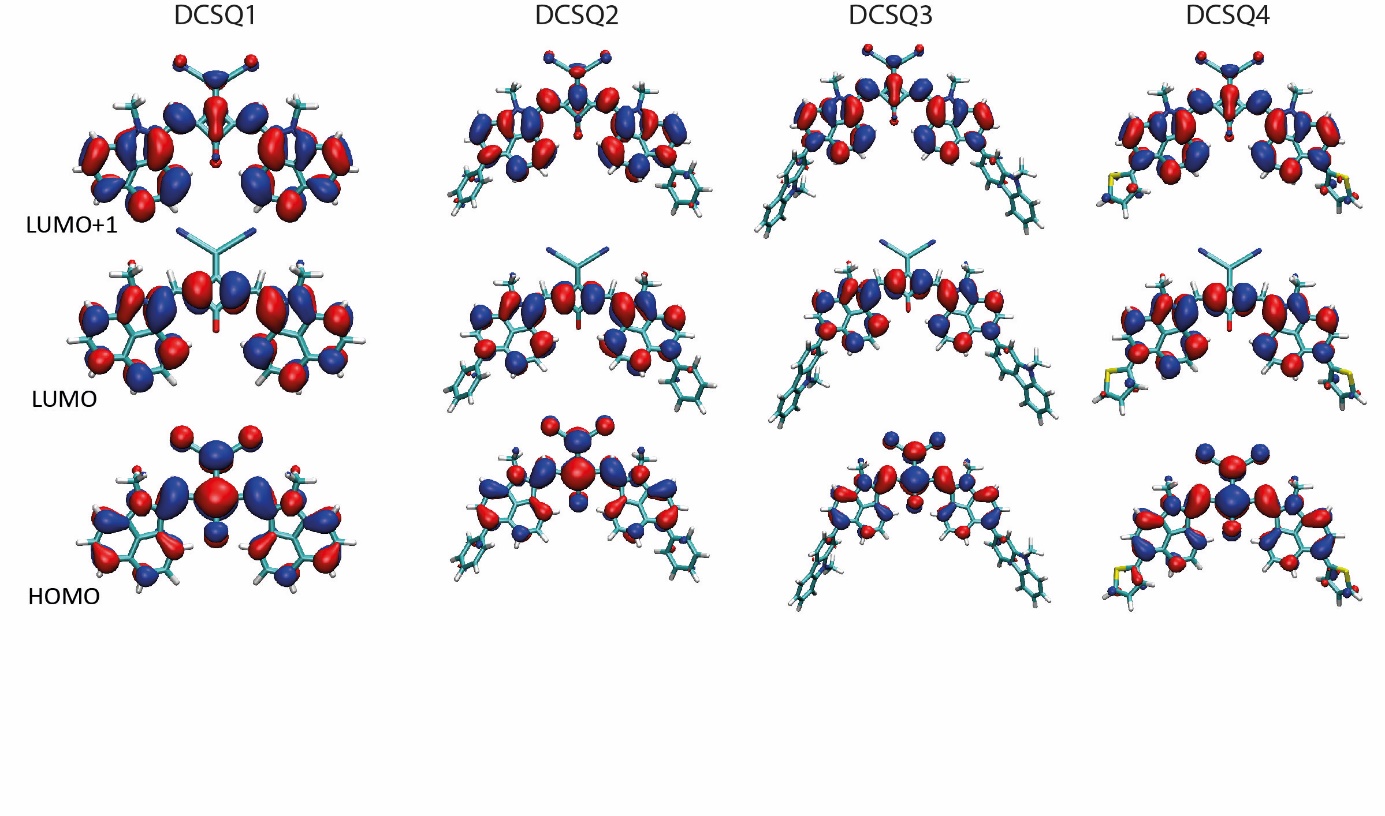
**Fig. S4.** (Top) Calculated frontier molecular orbitals for **SQ1** – **SQ4**. The electron distribution in the HOMO extends to the aromatic substituents. The conjugation with the benz[*cd*]indolein group is moderate for the thiophene substituent (**SQ4**), which also shows the most red-shifted absorption, while it is weak for the other substituents. The electron distribution in the LUMO is concentrated along the polymethine chain for all dyes, and the oxygen acceptor does not show a notable contribution. (Bottom) Calculated frontier molecular orbitals for **DCSQ1** – **DCSQ4**.

**Solvatochromism**

**
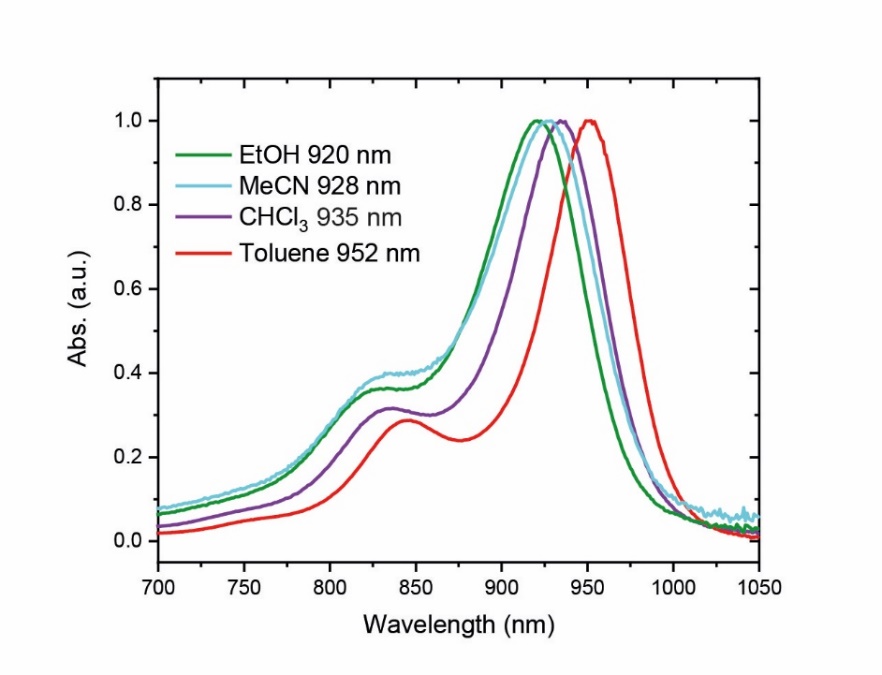
**

**Fig. S5.** Absorption spectra of **SQ4** in different solvents, demonstrating the negative solvatochromism of the dye.

**Cyclic Voltammograms of SQ dyes**

**
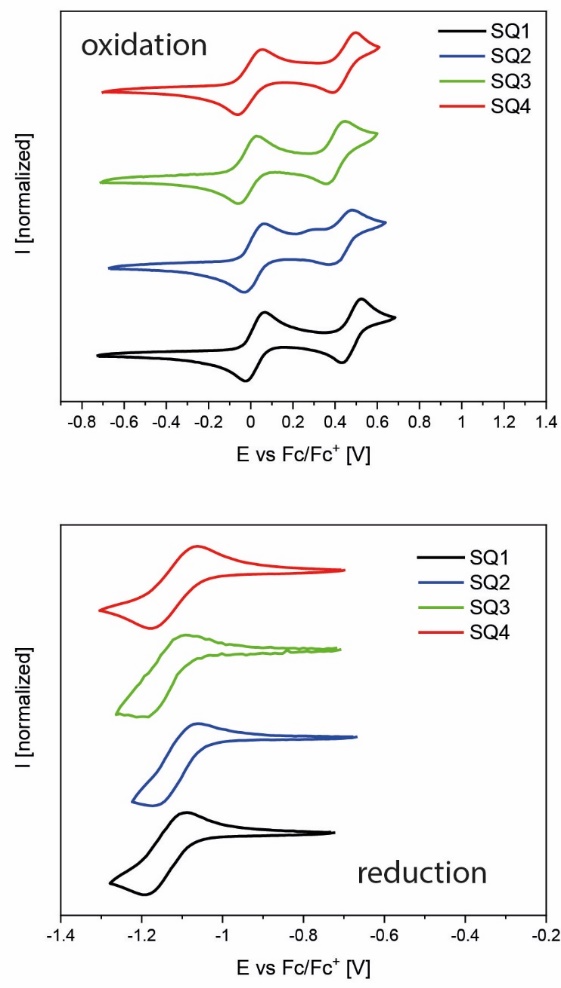
**

**Fig. S6.** Cyclic voltammograms of SQ dyes.

**Thermal properties**


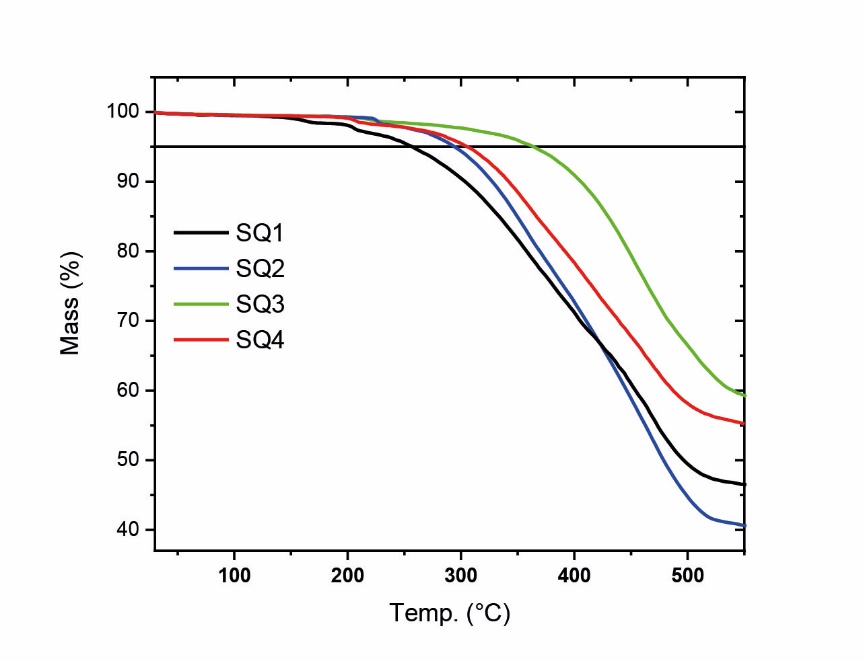


**Fig. S7.** Thermal gravimetric analysis of SQ dyes.


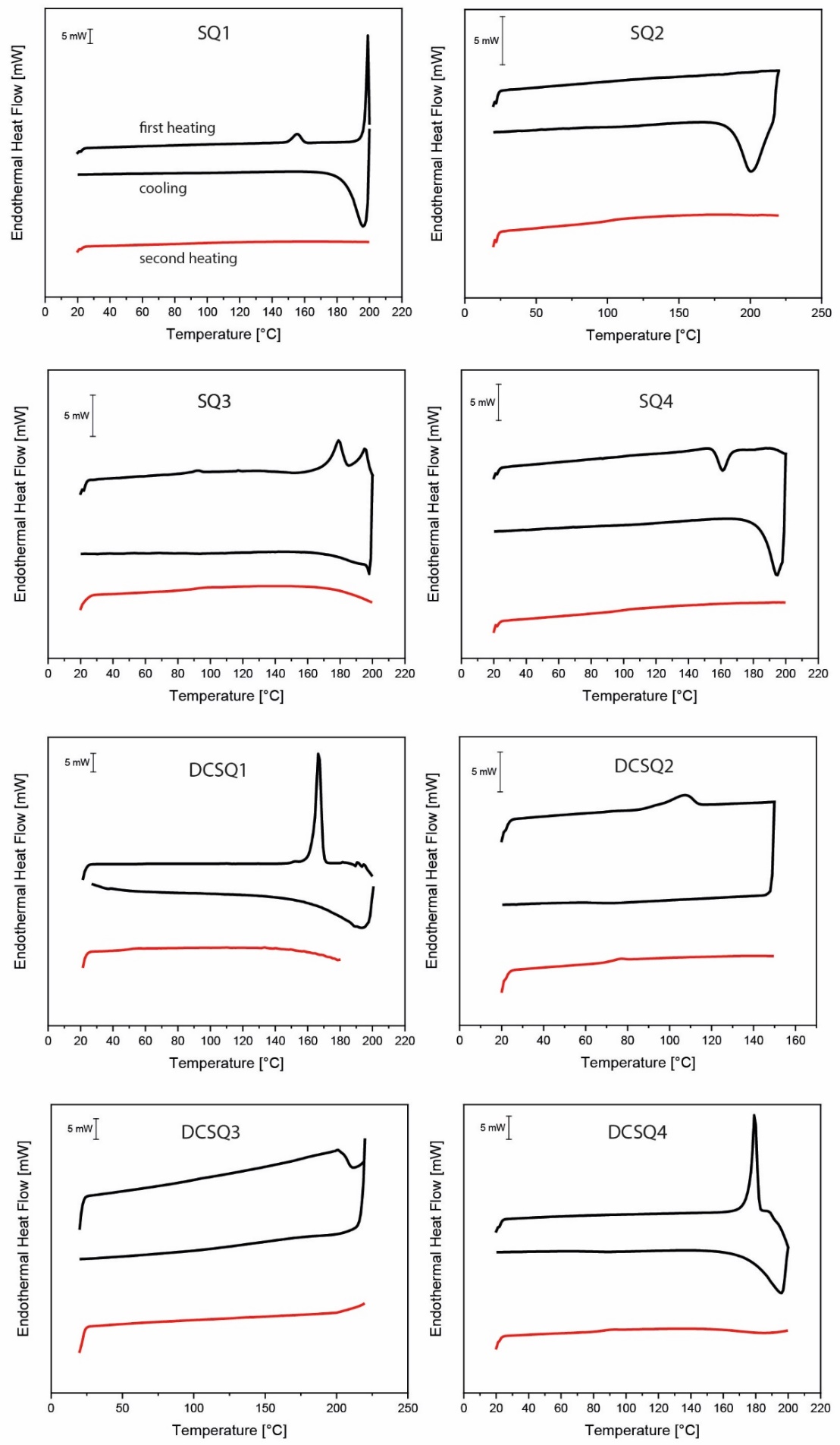


**Fig. S8.** DSC analysis of squaraine dyes. The first heating and cooling scans are shown in black, the second heating scan in red.

The thermal properties of **SQ1-4** and **DCSQ1-4** were investigated using DSC and TGA. Squaraine dye **SQ1** was precipitated into a crystalline state and showed a melting signal in the DSC at 155 °C. The material showed the onset of degradation at 198 °C during the TGA measurement.

In contrast to **SQ1**, the phenyl-substituted **SQ2** was precipitated into an amorphous state and exhibited a glassy to rubbery phase transition at 103 °C. The dye showed the onset of the degradation in the TGA at 222 °C.

Carbazolyl-substituted **SQ3** was also in an amorphous state after precipitation. In the DSC the dye first underwent a glass transition at 92 °C, followed by melting of imperfect crystalline material with a peak at 179 °C. This first melting was overlaid with a crystal perfection, a cold-crystallization, and the melting of the now proper crystalline sample with a peak at 195 °C. In the second DSC heating scan the material underwent another glass transition at 91 °C, demonstrating that the dye did not crystallize again after melting. The dye showed an onset degradation at 206 °C during the TGA measurement.

Thienyl-substituted **SQ4** was also in an amorphous state after precipitation. During the first heating scan in the DSC the material had a cold crystallization with a peak of 161 °C, but no melting signal was visible until 200 °C, at which temperature the dye started to degrade.

**DCSQ1** showed a melting point at an onset of 164 °C and a peak at 167 °C in the DSC. The degradation of the material started at an onset of 203 °C.

Phenyl-substituted **DCSQ2** showed a broad endothermal signal in the DSC with a peak at 107 °C and a glass transition at 72 °C in the second heating scan. The onset degradation in the TGA was at 200 °C.

Carbazolyl-substituted **DCSQ3** did not show any signals during the DSC measurement. The material showed a degradation onset at 229 °C during the TGA measurement.

The thienyl-substituted **DCSQ4** revealed a melting signal in the first DSC heating scan at a peak of 179 °C. The material showed an onset degradation at 205 °C in the TGA.

Combining the TGA results, it seems that the SQ and DCSQ dyes follow two different thermal degradation pathways. The SQ dyes experience two weight loss events, the DCSQ dyes three.

**Upconversion devices**

**
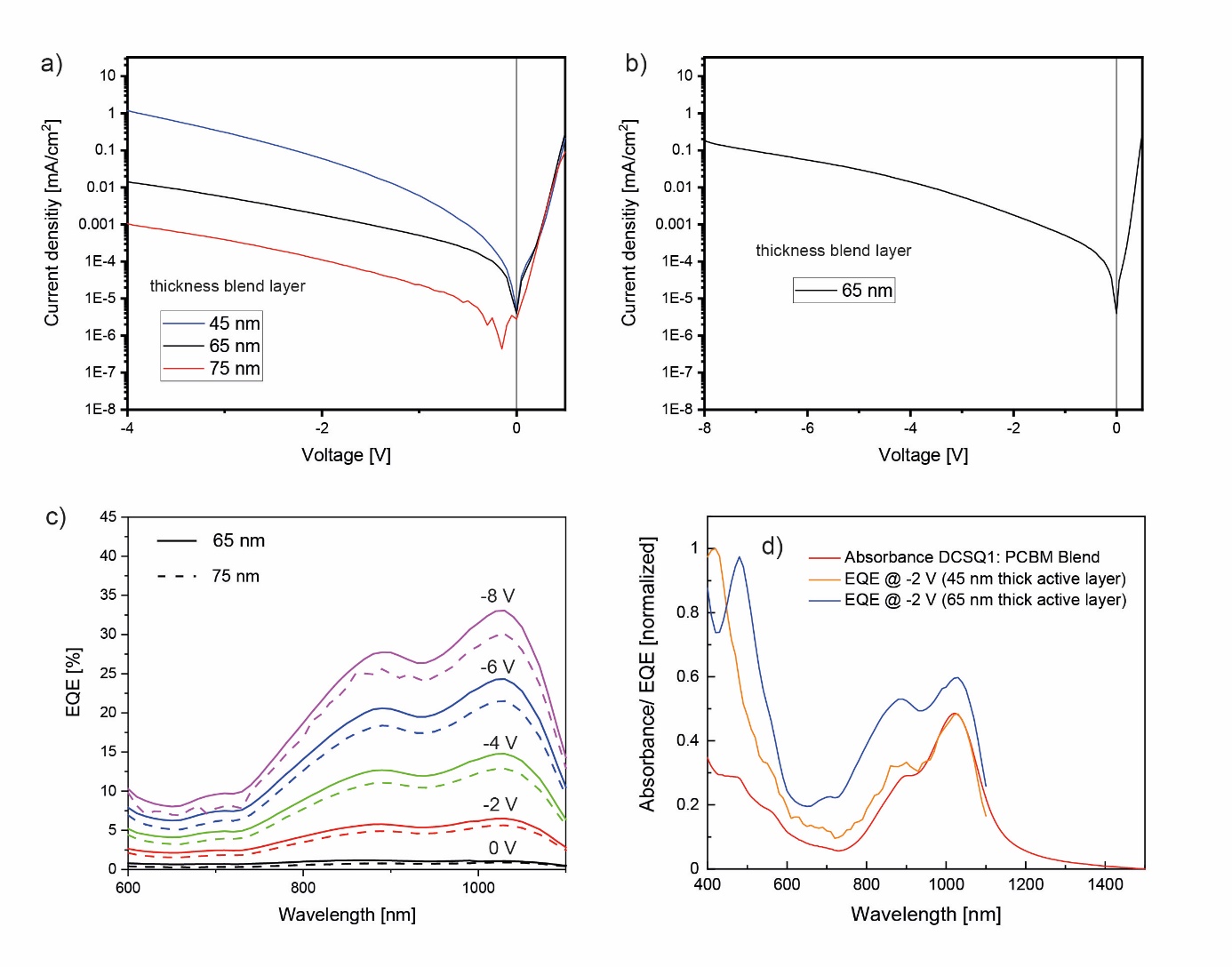
**

**Fig. S9.** (a,b) Dark currents of ITO/TiO_2_/**DCSQ1**:PCBM/MoO_3_/Ag PDs with different active layer thicknesses. (a) For thicknesses below around 45 nm the dark current is large, probably due to incomplete film formation. With increasing layer thickness, the dark current decreases. (b) for a blend layer thickness of 65 nm, the dark current increases to 0.2 mA cm^-2^ at an applied reverse bias of -8 V. (c) EQE spectra of PDs with active layer thicknesses of 65 nm and 75 nm. (d) EQE spectra of PDs with active layer thicknesses of 45 nm and 65 nm. In the NIR/SWIR spectral range, EQE spectra follow the film absorbance spectrum for both thicknesses. Data for the 45 nm thick film appear noisy because of the large dark current that could not be completely suppressed, even when chopping the light during the measurements. The absorbing layer is sandwiched between a weakly (glass/ITO) and strongly (Ag) reflecting interface, resulting in pronounced interference effects (weak microcavity) as function of the film thickness. The apparent EQE peak at 480 nm for the 65 nm thick film shifts to 420 nm when decreasing the film thickness to 45 nm.


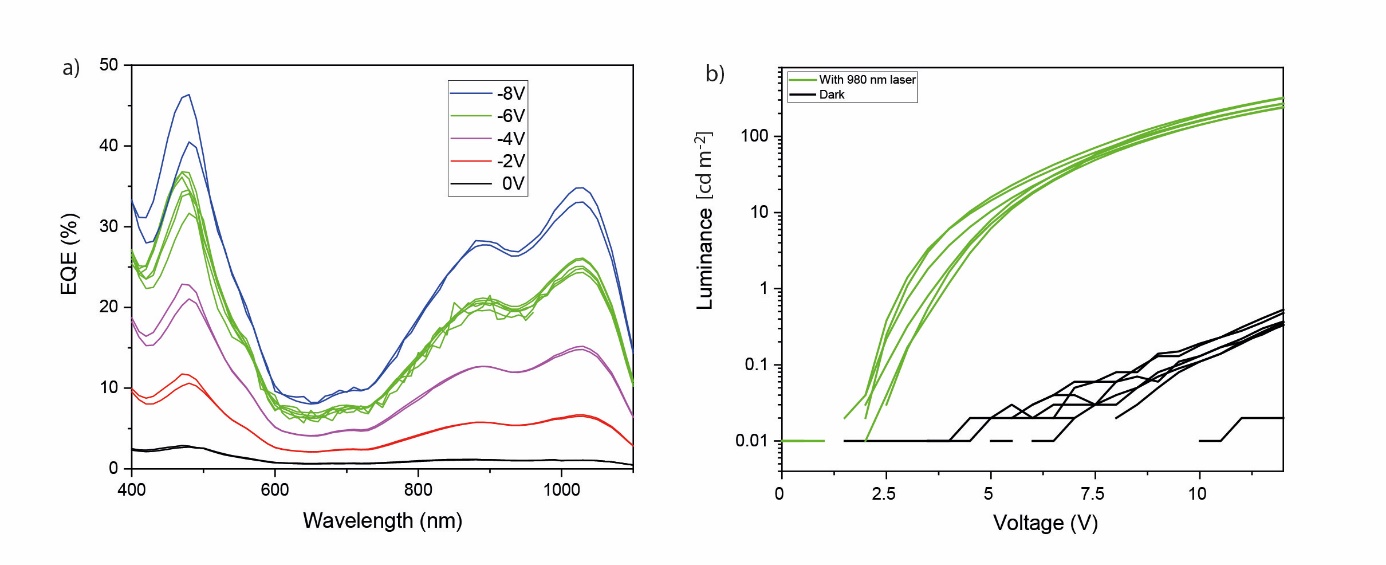


**Fig. S10.** (a) Reproducibility of device fabrication for PDs. (b) Luminance vs voltage trends from several OUC devices.


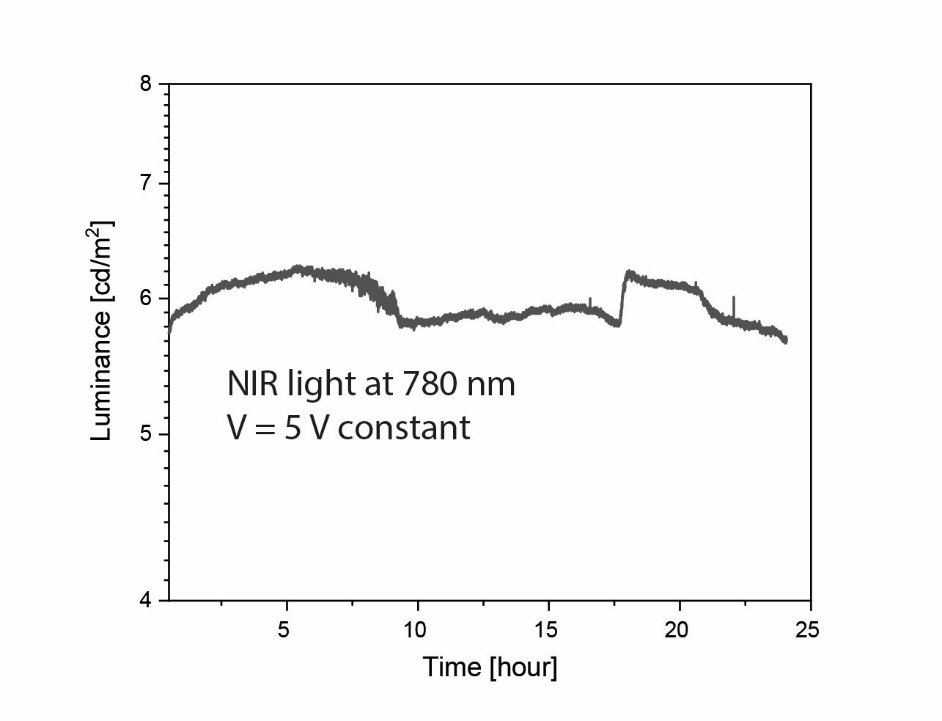


**Fig. S11.** Long-term stability of an OUC.

**MS spectra**


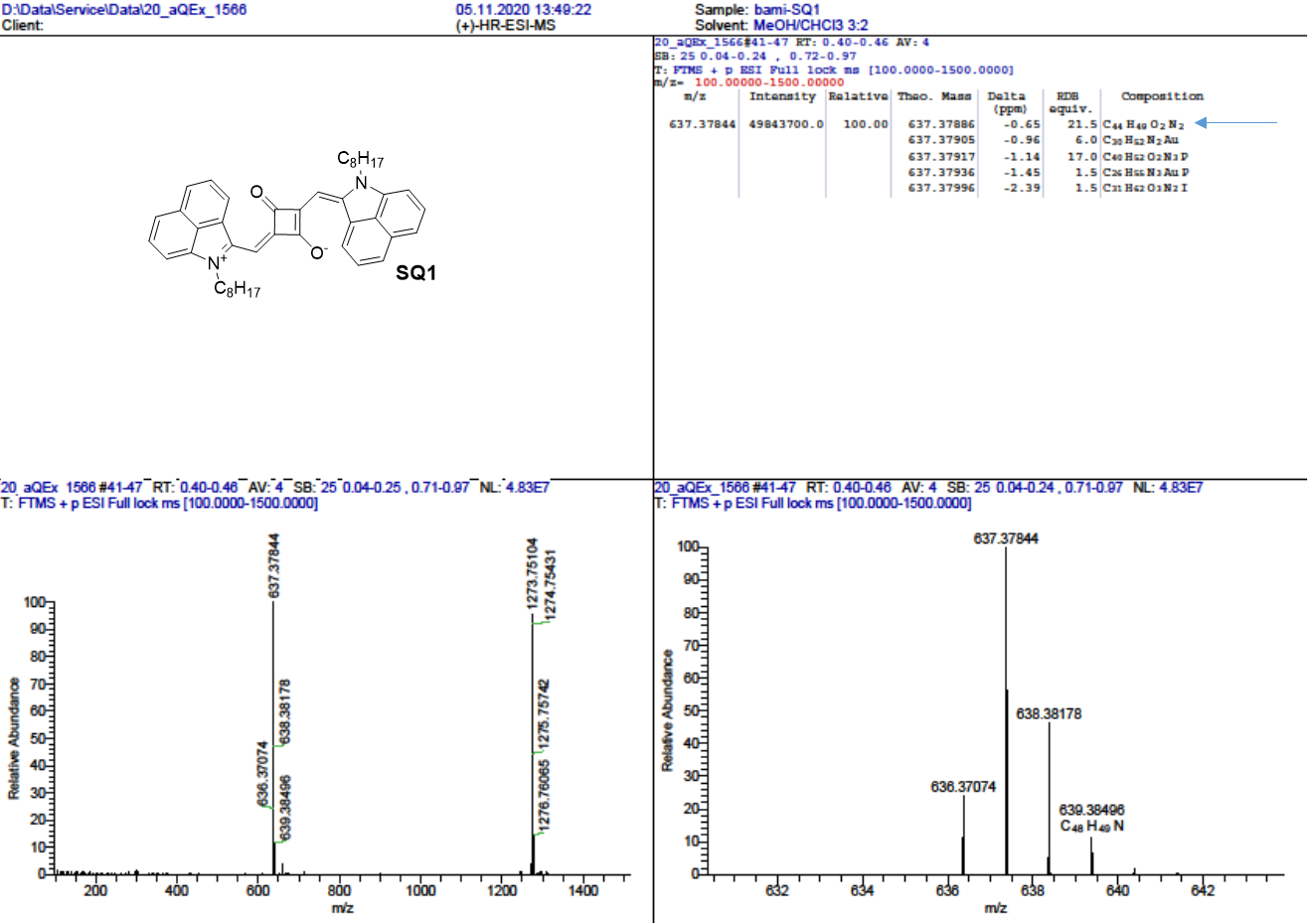


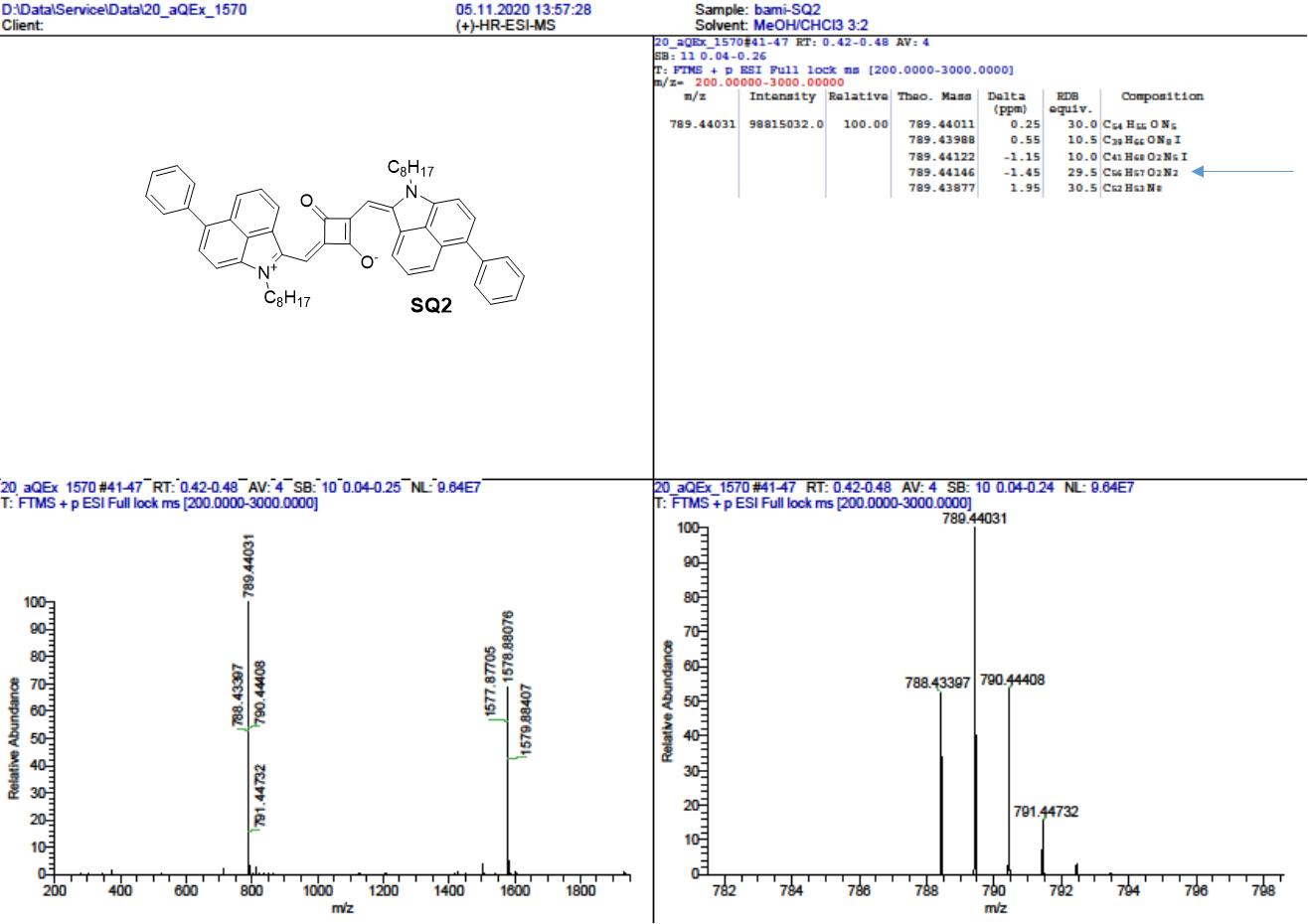


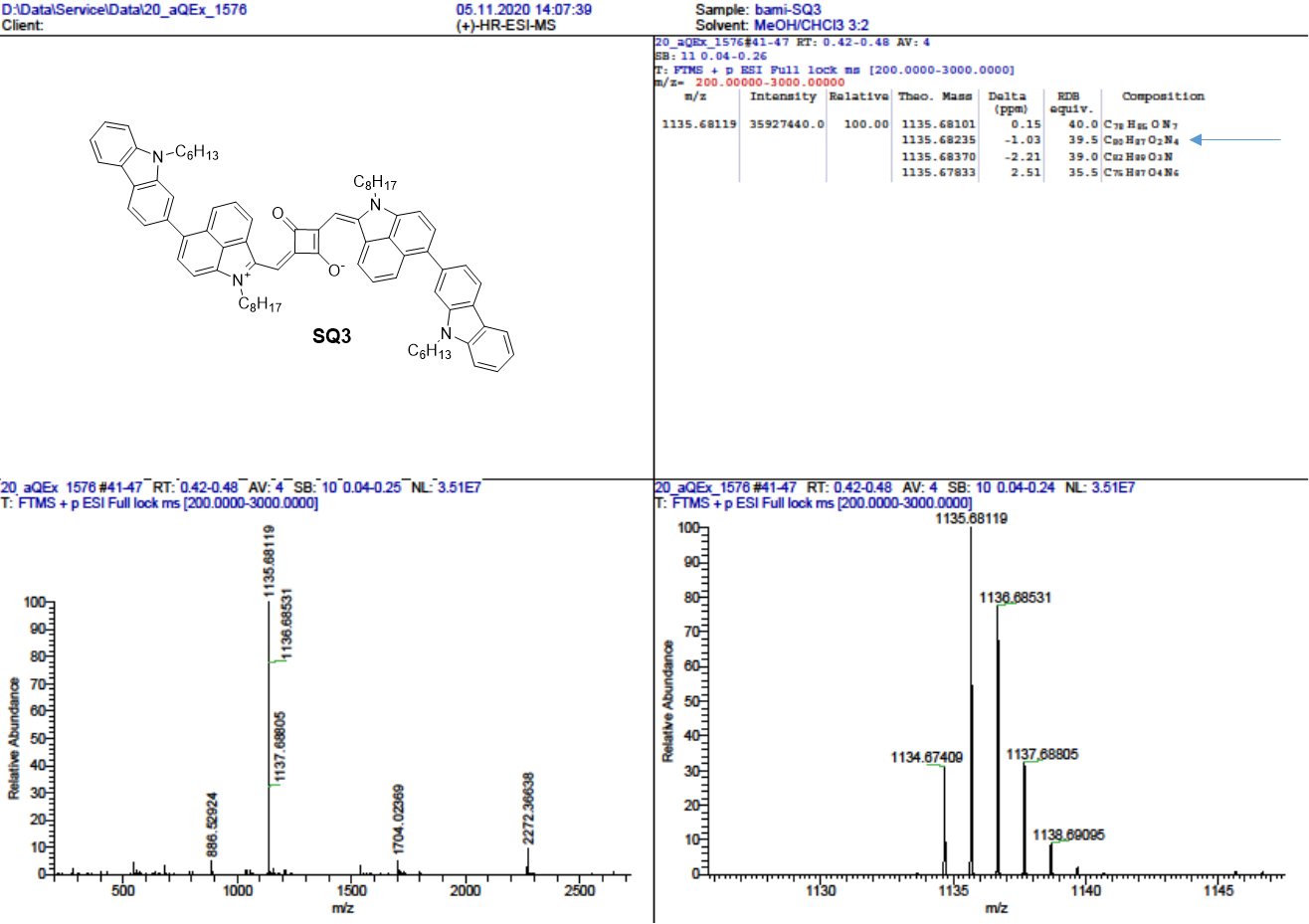


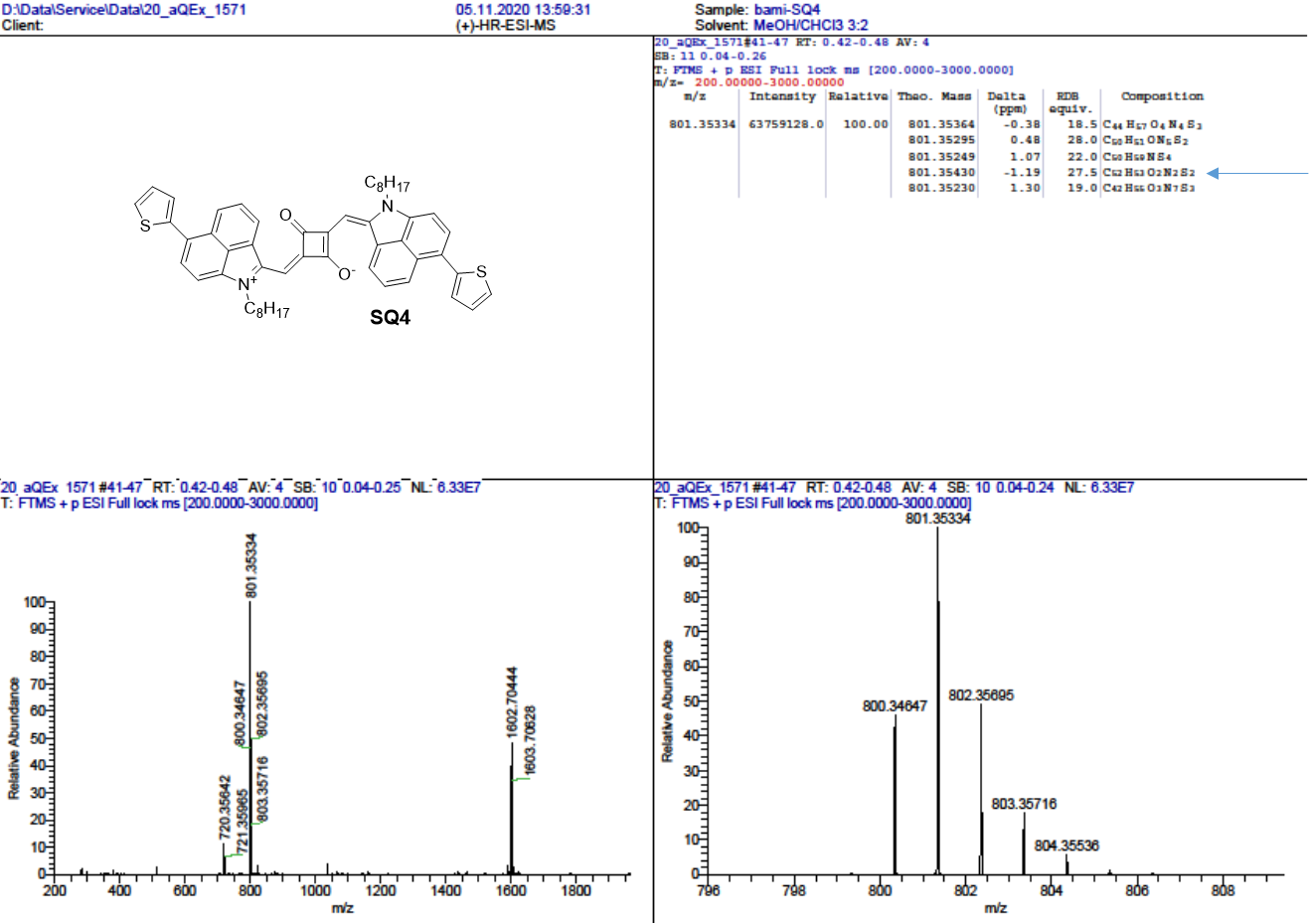


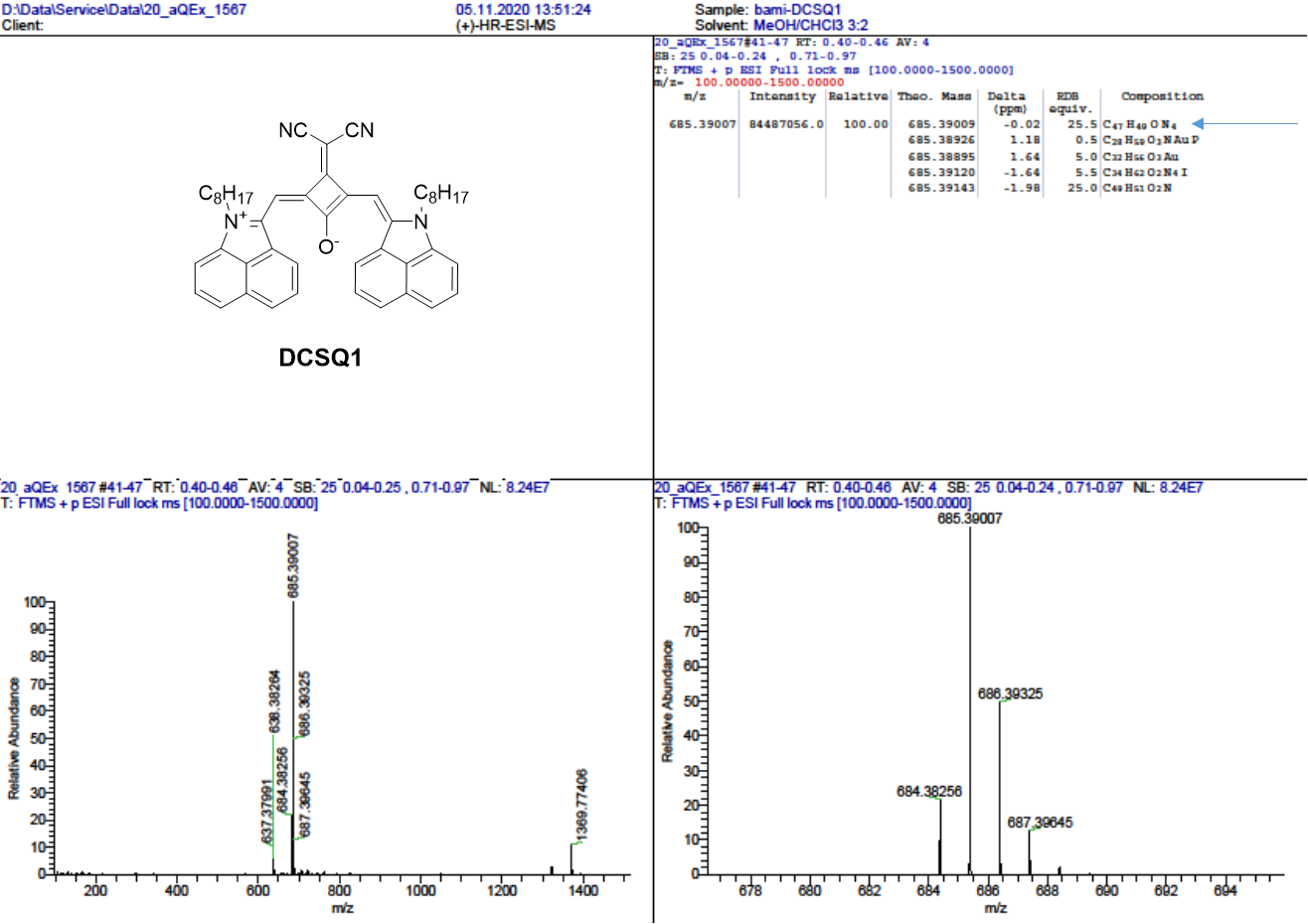


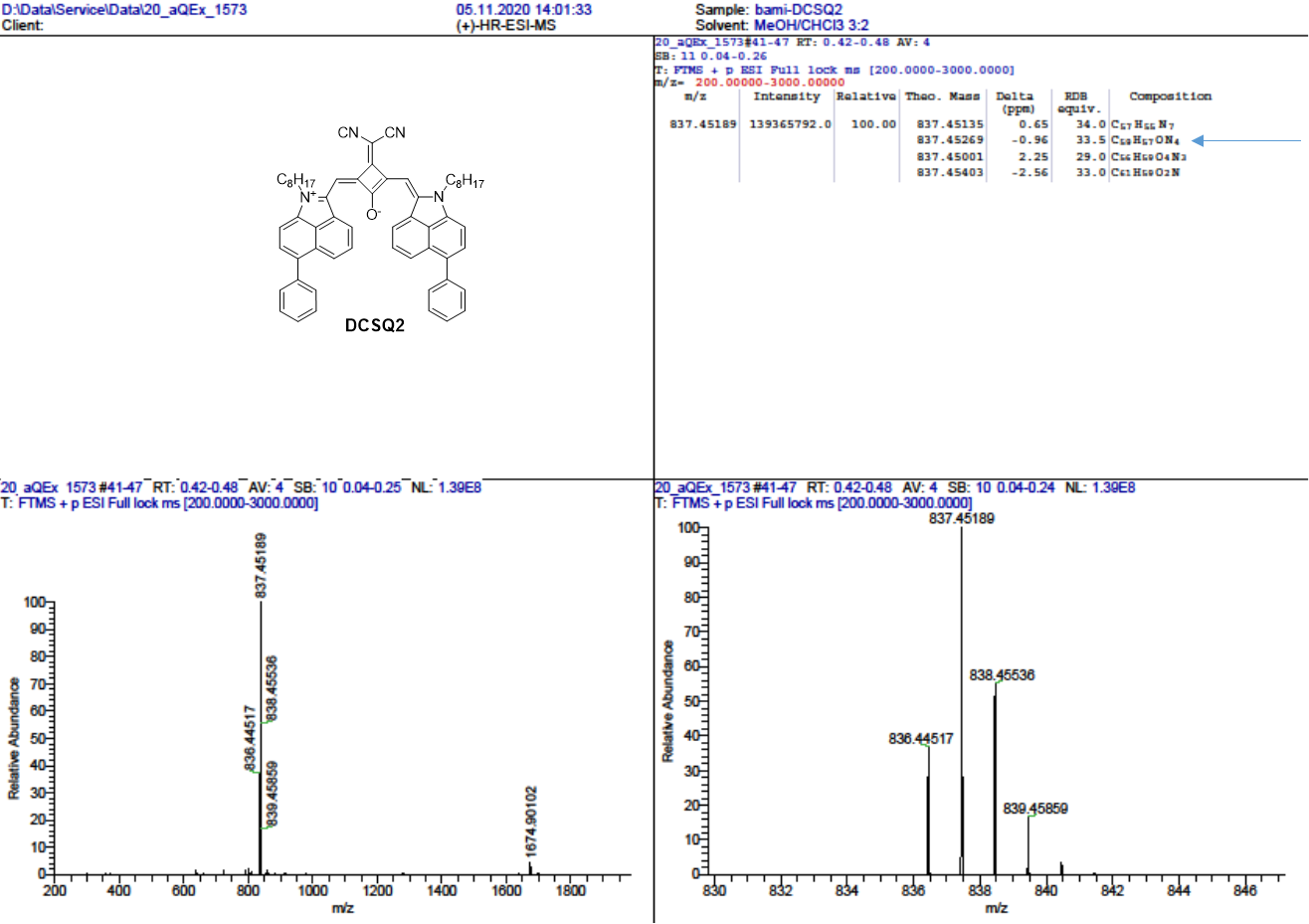


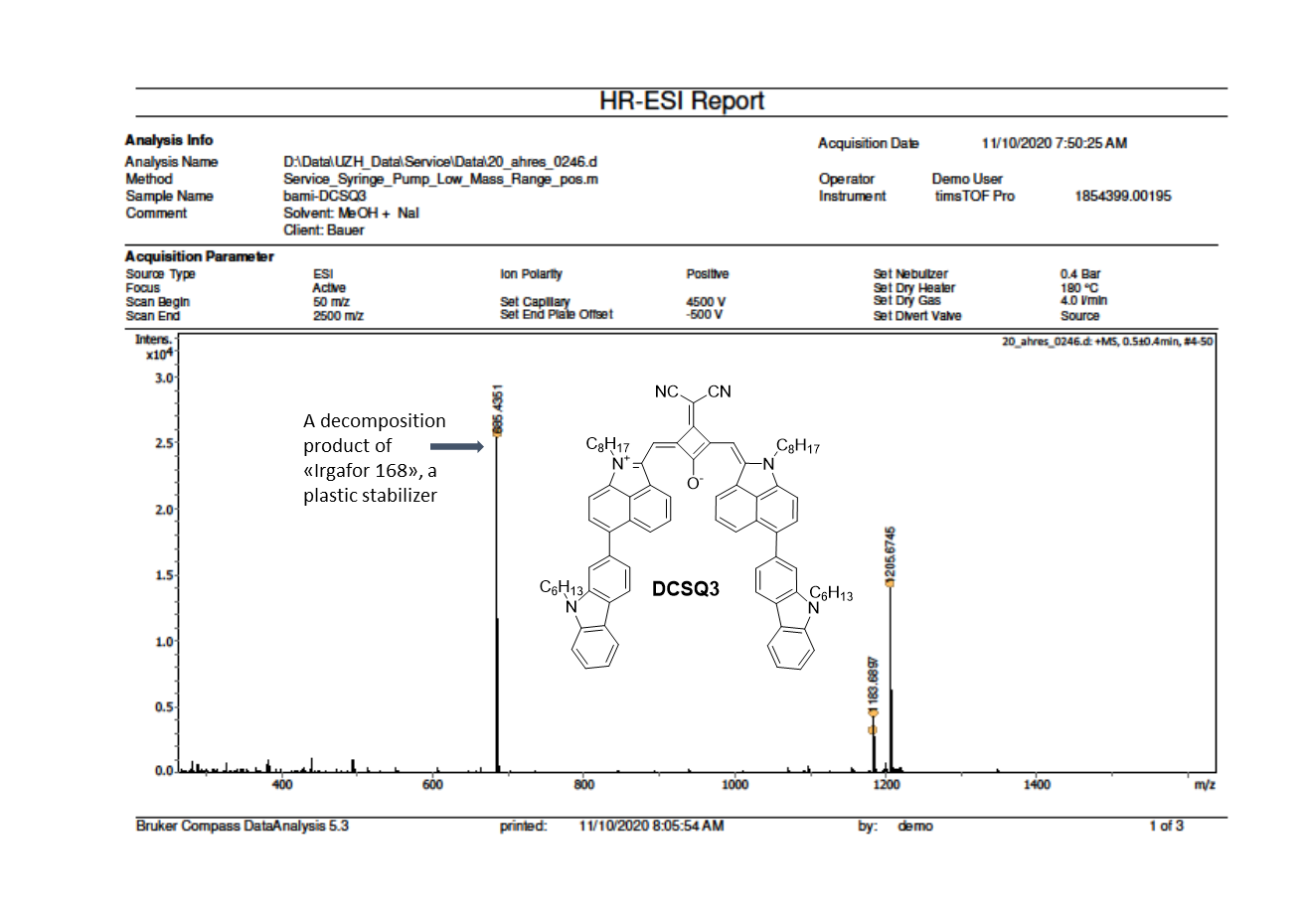


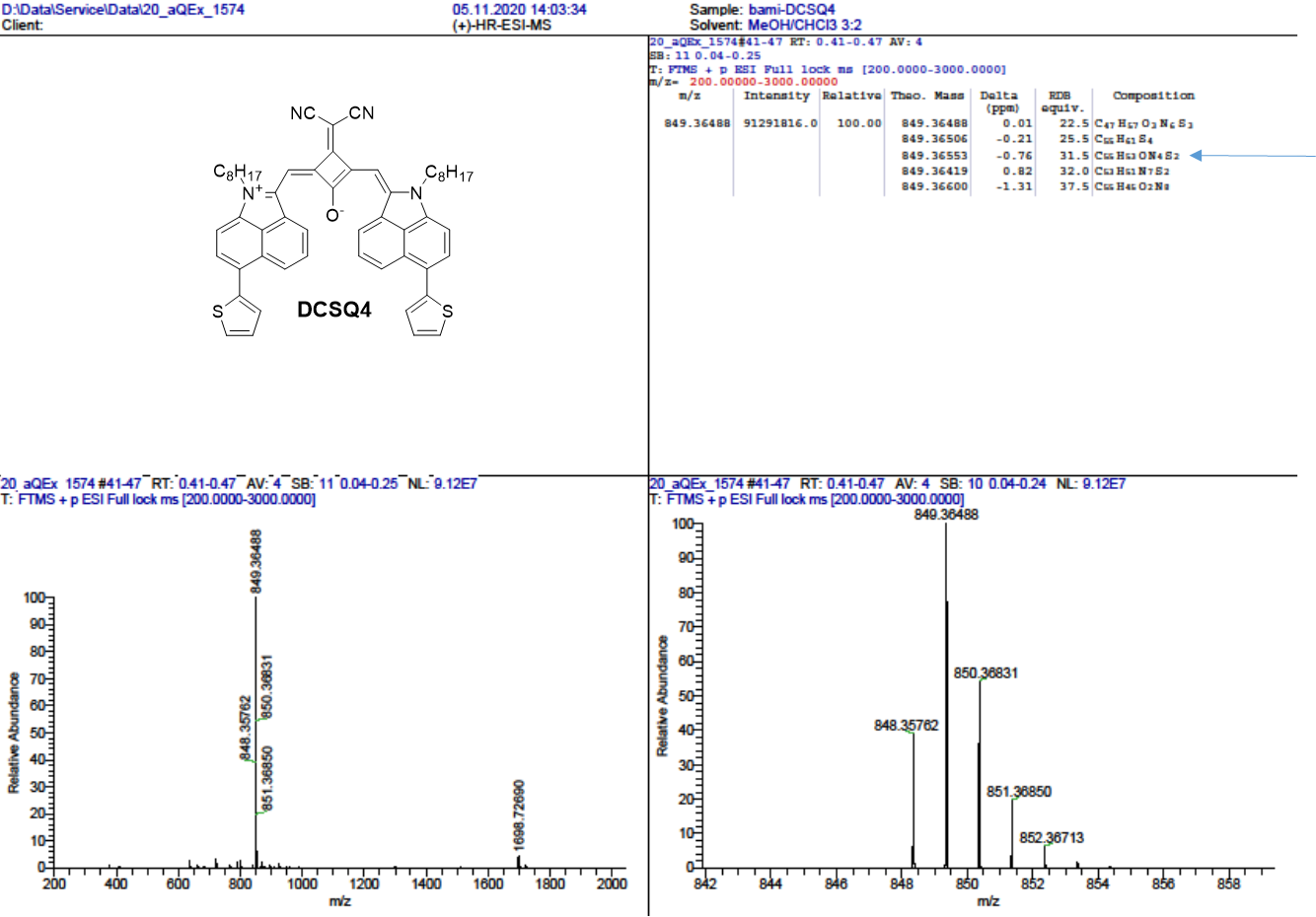


**^1^H and ^13^C NMR data with resonance assignments**


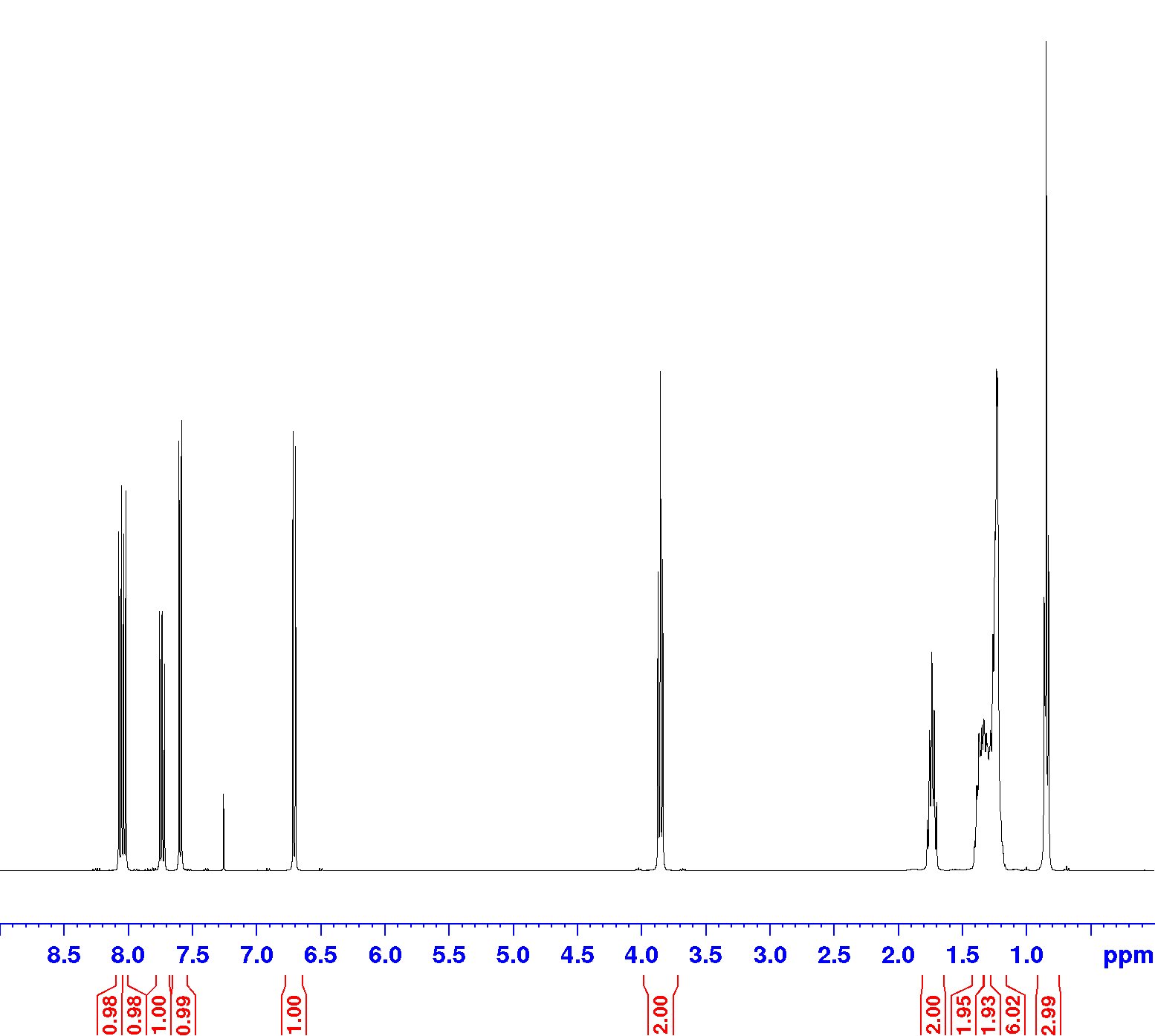


^1^H NMR (CDCl_3_, 400.2 MHz): *δ* 8.07 (d, *J* = 8.3, 1H, H-7); 8.03 (d, *J* = 7.0, 1H, H-9); 7.74 (dd, *J* = 8.3, 7.0, 1H, H-8); 7.60 (d, *J* = 7.4, 1H, H-2); 6.71 (d, *J* = 7.5, 1H, H-1); 3.85 (t, *J* = 7.3, 2H, H-12); 1.74 (quint, *J* = 7.3, 2H, H-13); 1.36 (m, 2H, H-14); 1.30 (m, 2H, H-15); 1.2-1.3 (m, 6H, H-16, 17, 18); 0.84 (t, *J* = 6.8, 3H, H-19).

DQF-COSY correlations: H-1→H-(2); H-2→H-(1); H-7→H-(8); H-8→H-(7, 9); H-9→H-(8); H-12→H-(13); H-13→H-(12, 14); H-14→H-(13, 15); H-15→H-(14); H-18→H-(19); H-19→H-(18).


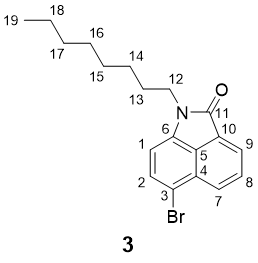

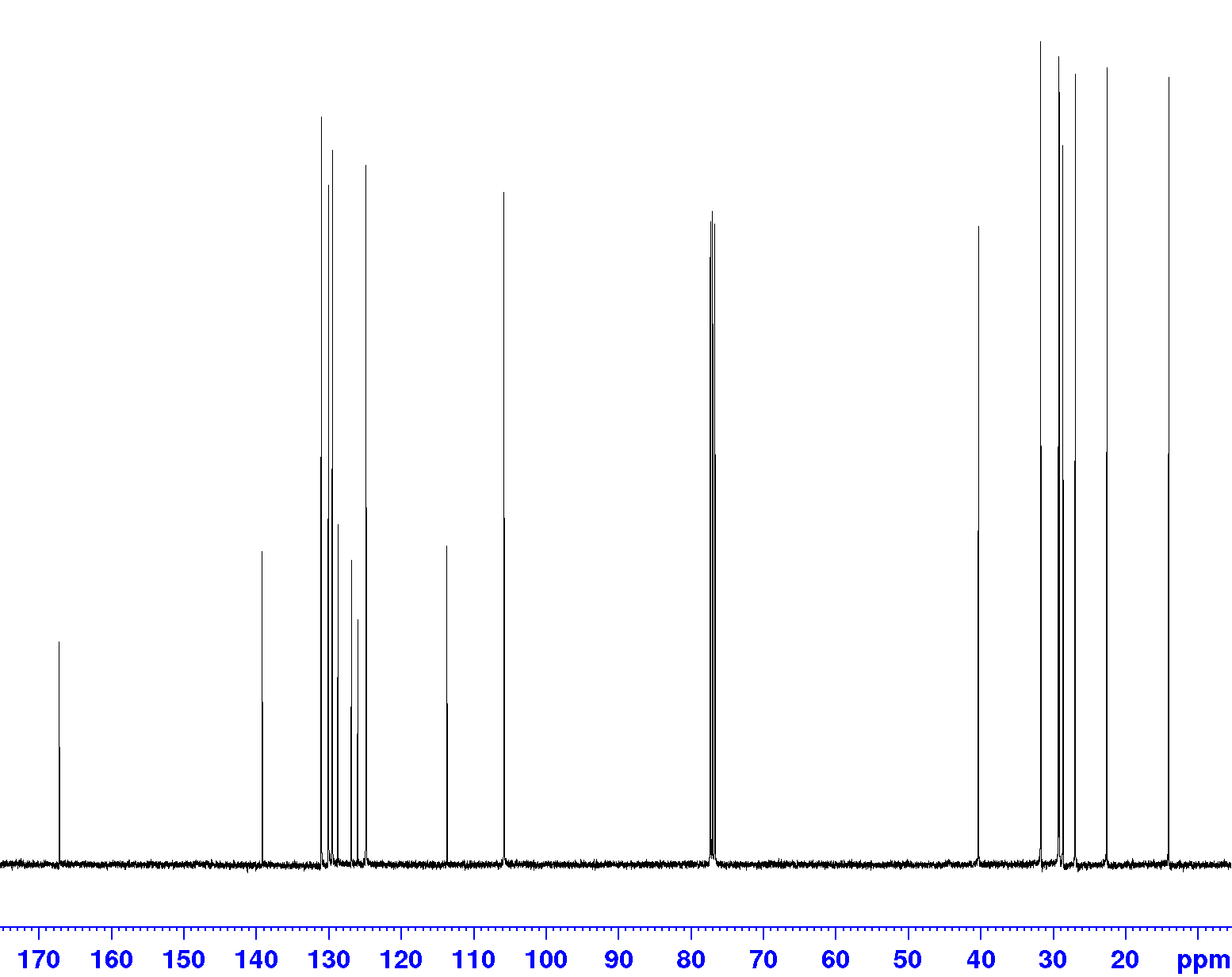


^13^C NMR (CDCl_3_, 100.6 MHz): *δ* 167.2 (s, C-11); 139.2 (s, C-6); 131.1 (d, C-2); 130.0 (d, C-7); 129.5 (d, C-8); 128.8 (s, C-4); 126.9 (s, C-10); 126.0 (s, C-5); 124.9 (d, C-9); 113.7 (s, C-3); 105.8 (d, C-1); 40.3 (t, C-12); 31.7 (t, C-17); 29.2 (t, C-15); 29.1 (t, C-16); 28.6 (t, C-13); 26.9 (t, C-14); 22.5 (t, C-18); 14.0 (q, C-19).

HMBC correlations: H-1→C-(3, 5, 6w); H-2→C-(3, 4, 6); H-7→C-(3, 5, 8w, 9); H-8→C-(4, 7w, 9w, 10, 11w); H-9→C-(5, 7, 11); H-12→C-(6, 11, 13, 14); H-13→C-(12, 14, 15); H-14→C-(12w, 15, 16); H-15→C-(14, 16); H-18→C-(16, 17, 19); H-19→C-(17, 18).

^
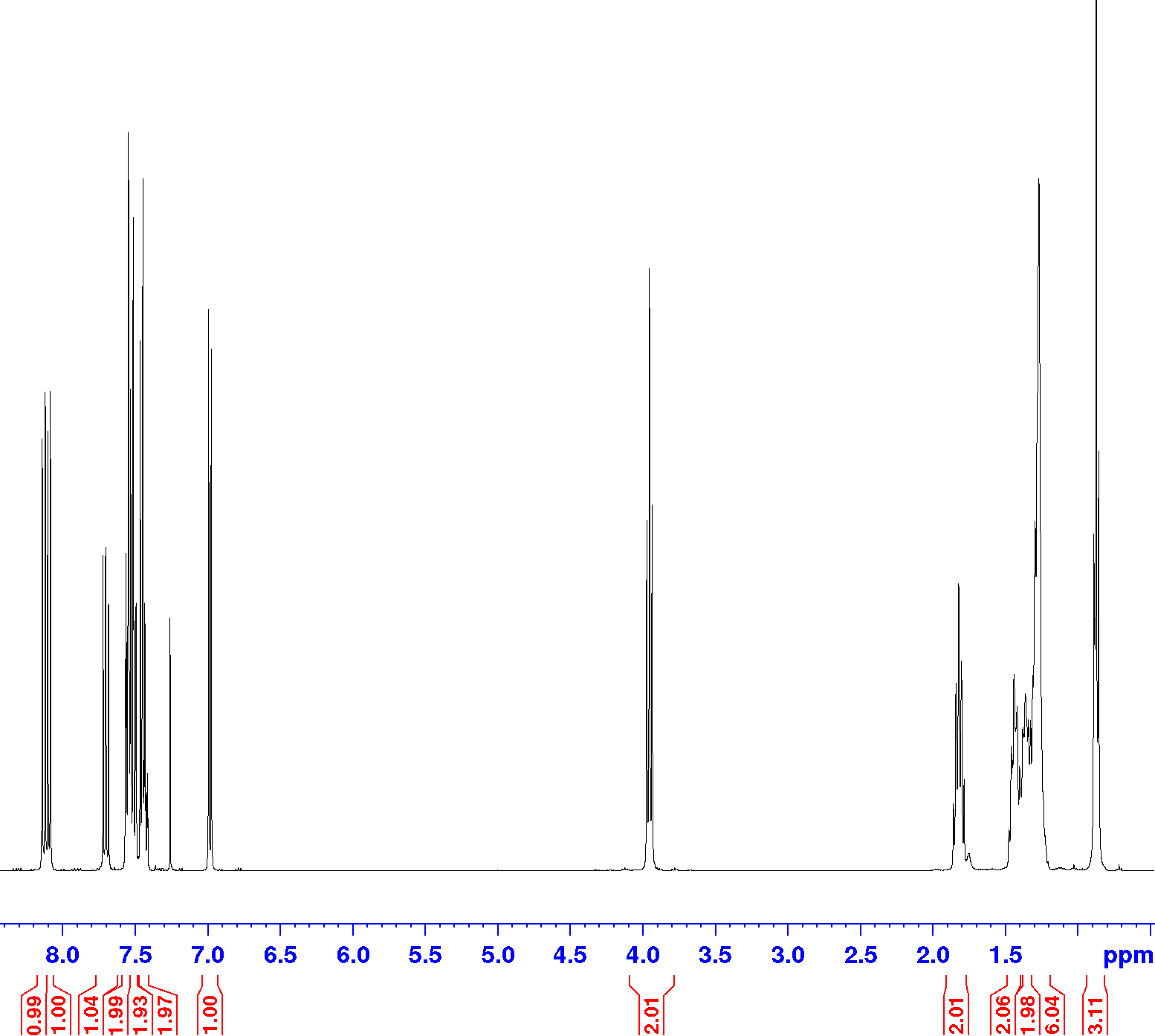
^

^1^H NMR (CDCl_3_, 400.2 MHz): *δ* 8.13 (d, *J* = 8.3, 1H, H-7); 8.09 (d, *J* = 6.9, 1H, H-9); 7.70 (dd, *J* = 8.3, 6.9, 1H, H-8); 7.56 (m, 2H, H-21); 7.51 (m, 2H, H-22); 7.46 (d, *J* = 7.3, 1H, H-2); 7.44 (m, 1H, H-23); 6.98 (d, *J* = 7.2, 1H, H-1); 3.96 (t, *J* = 7.3, 2H, H-12); 1.82 (m, *J* = 6.8, 2H, H-13); 1.44 (m, 2H, H-14); 1.36 (m, 2H, H-15); 1.2-1.3 (m, 6H, H-16, 17, 18); 0.87 (t, *J* = 6.8, 3H, H-19).

DQF-COSY correlations: H-1→H-(2); H-2→H-(1); H-7→H-(8); H-8→H-(7, 9); H-9→H-(8); H-12→H-(13); H-13→H-(12, 14); H-14→H-(13, 15); H-15→H-(14); H-18→H-(19);

H-19→H-(18); H-21→H-(22); H-22→H-(21, 23); H-23→H-(22).


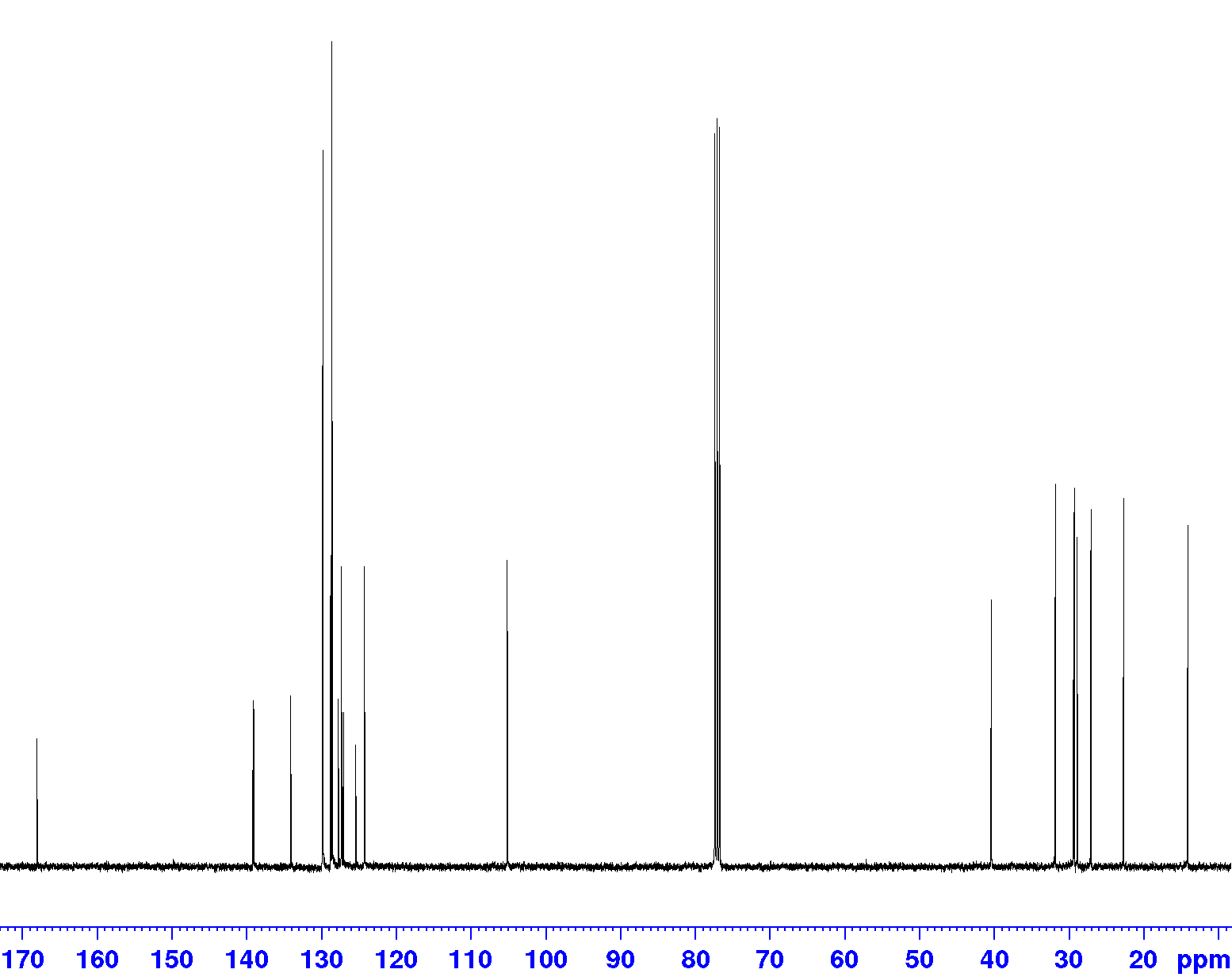


^13^C NMR (CDCl_3_, 100.6 MHz): *δ* 168.0 (s, C-11); 139.1 (s, C-20); 139.0 (s, C-6); 134.1 (s, C-3); 129.9 (d, C-7); 129.8 (d, C-21); 128.7 (d, C-8); 128.6 (d, C-22); 128.6 (d, C-2); 127.7 (s, C-4); 127.3 (d, C-23); 127.0 (s, C-10); 125.4 (s, C-5); 124.2 (d, C-9); 105.1 (d, C-1); 40.3 (t, C-12); 31.8 (t, C-17); 29.3 (t, C-15); 29.2 (t, C-16); 28.8 (t, C-13); 27.0 (t, C-14); 22.6 (t, C-18); 14.1 (q, C-19).

HMBC correlations: H-1→C-(2w, 3, 5, 6); H-2→C-(1, 4, 6, 20); H-7→C-(3, 5, 8w, 9); H-8→C-(4, 7w, 9w, 10); H-9→C-(5, 7, 8w, 11); H-12→C-(6, 11, 13, 14); H-13→C-(12, 14, 15); H-14→C-(12, 13w, 15, 16); H-21→C-(3, 21, 23); H-22→C-(20, 22); H-23→C-(21).


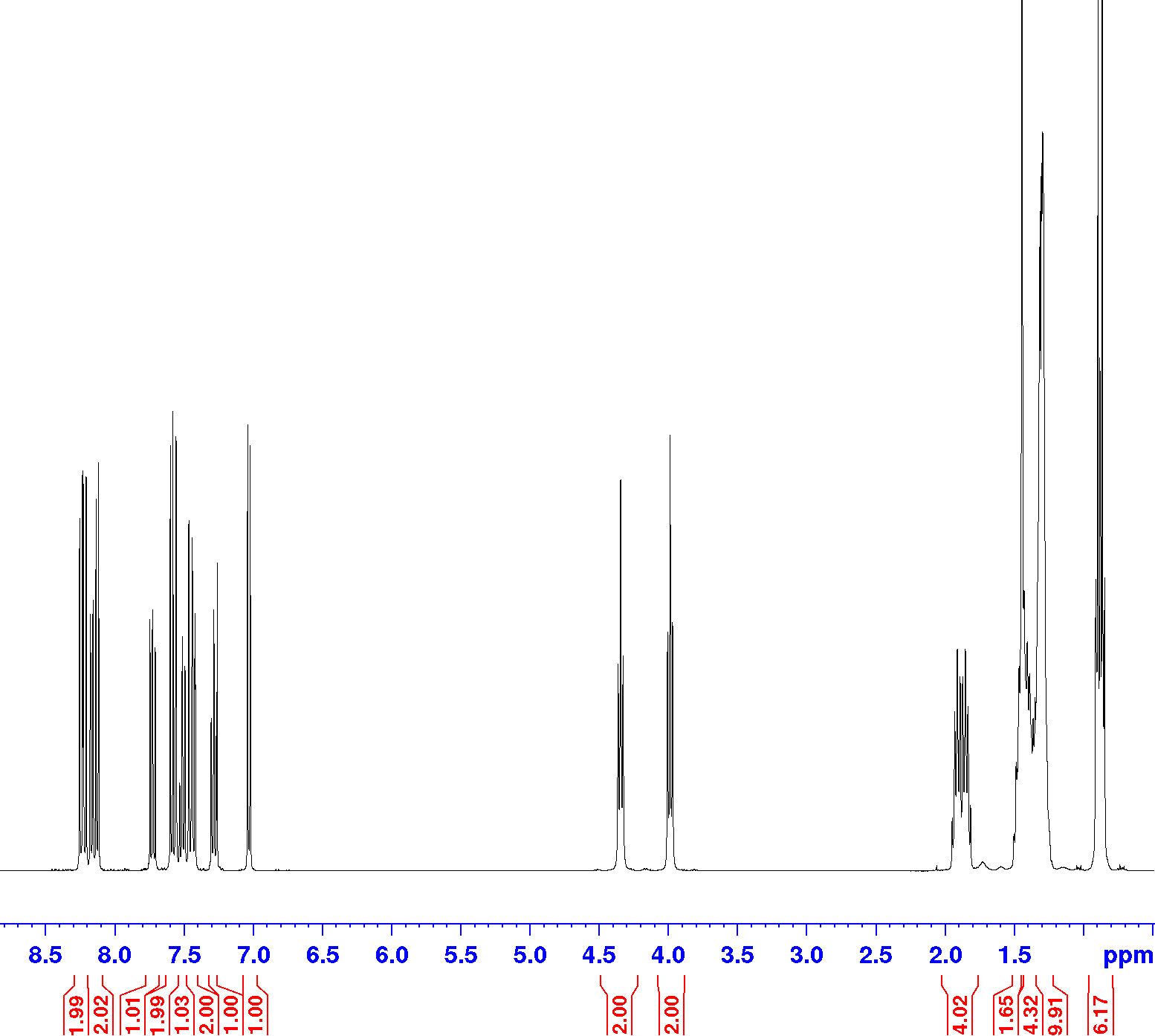


^1^H NMR (CDCl_3_, 400.2 MHz): *δ* 8.24 (d, *J* = 8.3, 1H, H-7); 8.22 (d, *J* = 8.0, 1H, H-23); 8.17 (m, 1H, H-26); 8.13 (d, *J* = 6.9, 1H, H-9); 7.73 (dd, *J* = 8.3/6.9, 1H, H-8); 7.59 (d, *J* = 7.3, 1H, H-2); 7.56 (d, *J* = 1.3, 1H, H-20); 7.51 (m, 1H, H-28); 7.47 (m, 1H, H-29); 7.43 (dd, *J* = 8.0/1.3, 1H, H-22); 7.29 (m, 1H, H-27); 7.03 (d, *J* = 7.3, 1H, H-1); 4.34 (t, *J* = 7.2, 2H, H-32); 3.99 (t, *J* = 7.2, 2H, H-12); 1.91 (m, 2H, H-33); 1.85 (m, 2H, H-13); 1.46 (m, 2H, H-14); 1.42 (m, 2H, H-34); 1.38 (m, 2H, H-15); 1.31 (m, 2H, H-35); 1.2-1.3 (m, 8H, H-16, 17, 18, 36); 0.89 (t, *J* = 7.1, 3H, H-19); 0.86 (t, *J* = 7.1, 3H, H-37).

DQF-COSY correlations: H-1→H-(2); H-2→H-(1); H-7→H-(8); H-8→H-(7, 9); H-9→H-(8); H-12→H-(13); H-13→H-(12, 14); H-14→H-(13, 15); H-15→H-(14, 16); H-16→H-(15); H-18→H-(19); H-19→H-(18); H-22→H-(23); H-23→H-(22); H-26→H-(27); H-27→H-(26, 28); H-28→H-(27, 29); H-29→H-(28); H-32→H-(33); H-33→H-(32, 34); H-34→H-(33, 35); H-35→H-(34); H-36→H-(37); H-37→H-(36).

^
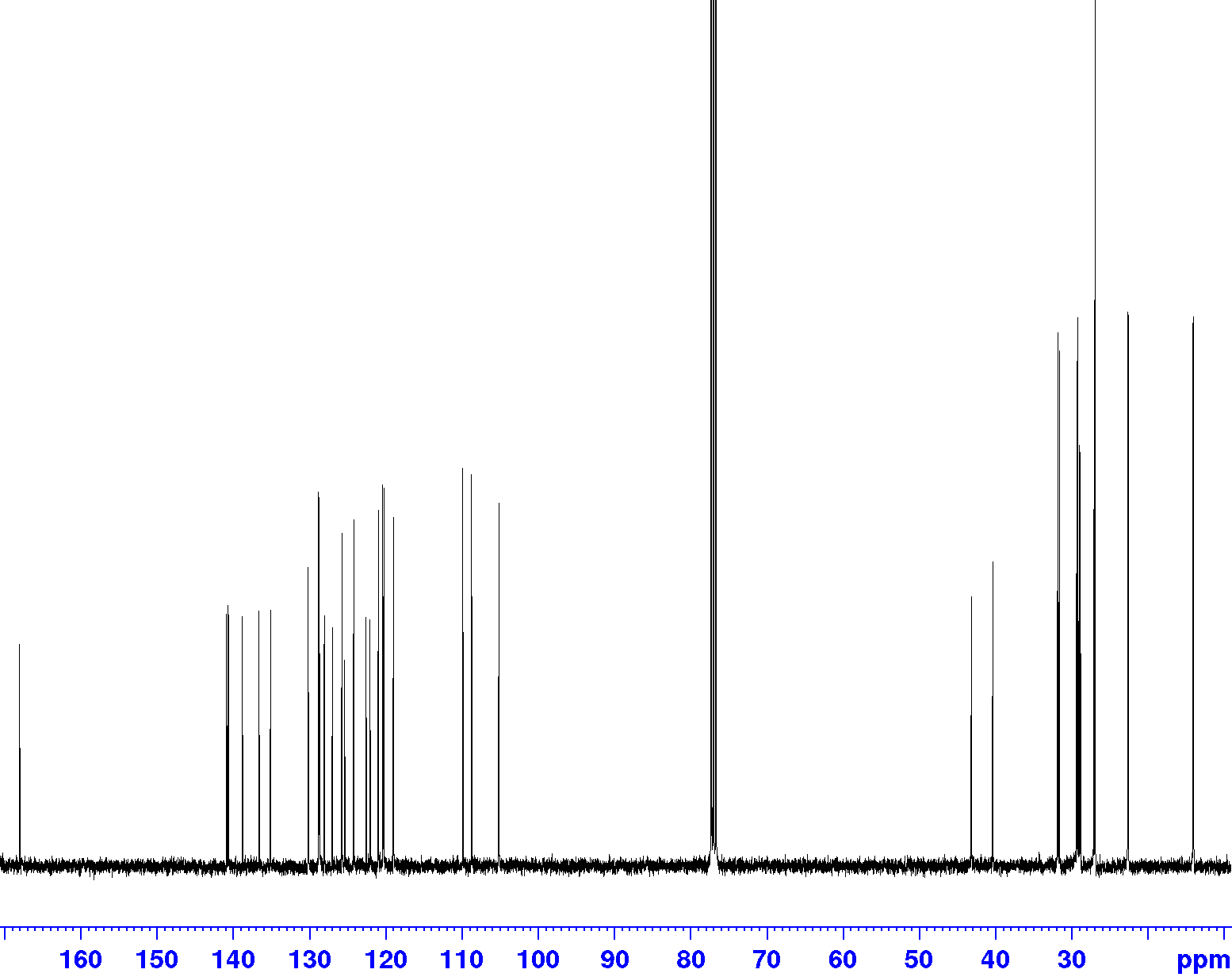
^

^13^C NMR (CDCl_3_, 100.6 MHz): *δ* 168.1 (s, C-11); 140.9 (s, C-30); 140.7 (s, C-31); 138.8 (s, C-6); 136.7 (s, C-21); 135.1 (s, C-3); 130.2 (d, C-7); 128.8 (d, C-2); 128.7 (d, C-8); 128.1 (s, C-4); 127.0 (s, C-10); 125.8 (d, C-28); 125.4 (s, C-5); 124.2 (d, C-9); 122.6 (s, C-25); 122.1 (s, C-24); 121.0 (d, C-22); 120.4 (d, C-26); 120.3 (d, C-23); 119.0 (d, C-27); 109.9 (d, C-20); 108.7 (d, C-29); 105.2 (d, C-1); 43.2 (t, C-32); 40.3 (t, C-12); 31.8 (t, C-17); 31.6 (t, C-35); 29.3 (t, C-15); 29.2 (t, C-16); 29.0 (t, C-33); 28.9 (t, C-13); 27.0 (t, C-14, 34); 22.6 (t, C-18, 36); 14.1 (q, C-19); 14.0 (q, C-37).

HMBC correlations: H-1→C-(3, 5, 6); H-2→C-(1w, 4, 6, 21); H-7→C-(3, 5, 9); H-8→C-(4, 7w, 9w, 10, 11w); H-9→C-(5, 7, 11); H-12→C-(6, 11, 13, 14); H-13→C-(12, 14, 15); H-14→C-(12, 13, 15, 16); H-19→C-(18, 19); H-20→C-(3, 22, 24); H-22→C-(3, 20, 24); H-23→C-(21, 25, 31); H-26→C-(24, 28, 30); H-27→C-(25, 28, 29); H-28→C-(26, 27, 30); H-29→C-(25, 27); H-32→C-(30, 31, 33, 34); H-33→C-(32, 34, 35); H-34→C-(32, 35, 36); H-37→C-(35, 36).


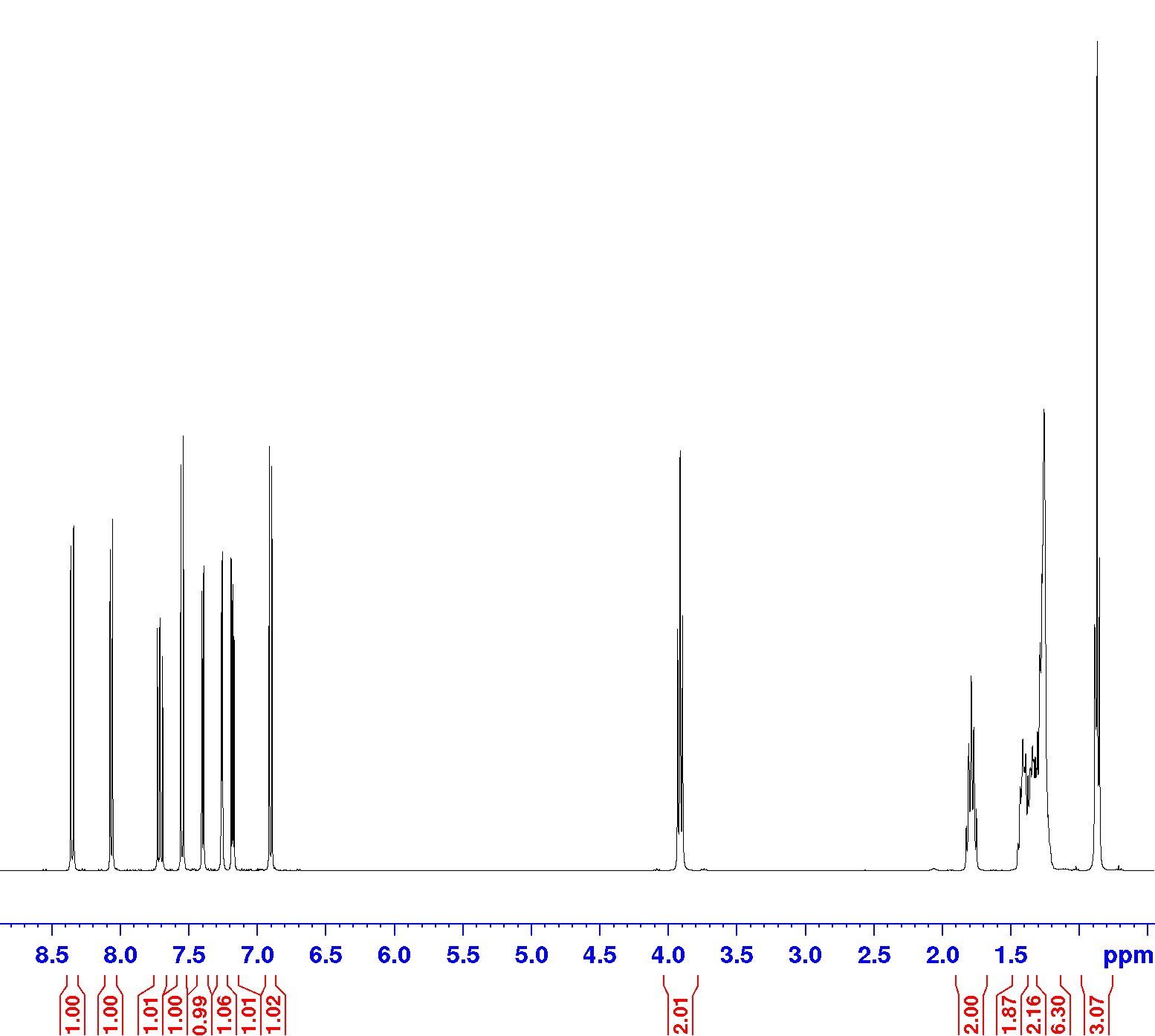


^1^H NMR (CDCl_3_, 400.2 MHz): *δ* 8.35 (d, *J* = 8.3, 1H, H-7); 8.07 (d, *J* = 7, 1H, H-9); 7.71 (dd, *J* = 8.3/7.0, 1H, H-8); 7.55 (d, *J* = 7.4, 1H, H-2); 7.40 (dd, *J* = 5.1, 1.2, 1H, H-23); 7.26 (dd, *J* = 3.5, 1.2, 1H, H-21); 7.18 (dd, *J* = 5.1, 3.5, 1H, H-22); 6.90 (d, *J* = 7.4, 1H, H-1); 3.91 (t, *J* = 7.3, 2H, H-12); 1.79 (quint, *J* = 7.3, 2H, H-13); 1.39 (m, 2H, H-14); 1.33 (m, 2H, H-15); 1.2-1.3 (m, 6H, H-16, 17, 18); 0.87 (t, *J* = 6.9, 3H, H-19).

DQF-COSY correlations: H-1→H-(2); H-2→H-(1); H-7→H-(8); H-8→H-(7, 9); H-9→H-(8); H-12→H-(13); H-13→H-(12, 14); H-14→H-(13, 15); H-15→H-(14); H-18→H-(19); H-19→H-(18); H-21→H-(22, 23w); H-22→H-(21, 23); H-23→H-(21w, 22).


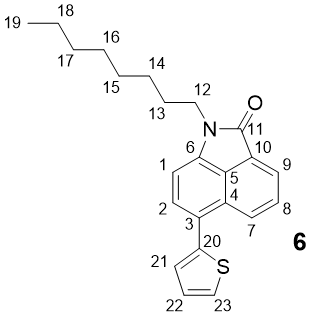

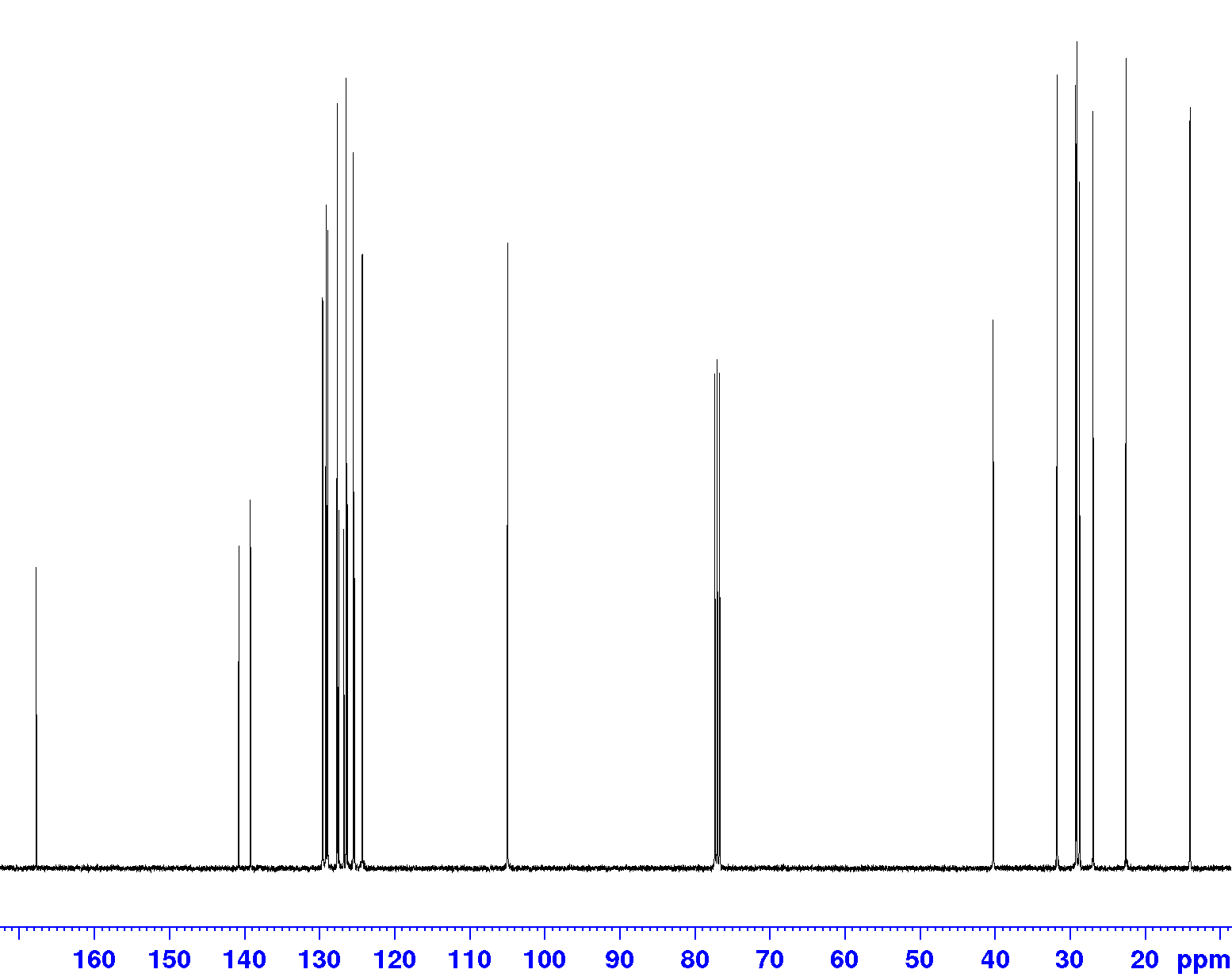


^13^C NMR (CDCl_3_, 100.6 MHz): *δ* 167.8 (s, C-11); 140.8 (s, C-20); 139.2 (s, C-6); 129.6 (d, C-7); 129.2 (d, C-2); 128.9 (d, C-8); 127.6 (d, C-22); 127.4 (s, C-4); 126.8 (s, C-10); 126.5 (d, C-21); 126.3 (s, C-3); 125.5 (d, C-23); 125.3 (s, C-5); 124.3 (d, C-9); 104.9 (d, C-1); 40.2 (t, C-12); 31.7 (t, C-17); 29.2 (t, C-15); 29.1 (t, C-16); 28.7 (t, C-13); 26.9 (t, C-14); 22.5 (t, C-18); 14.0 (q, C-19).

HMBC correlations: H-1→C-(2w, 3, 5, 6); H-2→C-(1, 4, 6, 7w, 20); H-7→C-(3, 5, 6w, 8, 9); H-8→C-(4, 7, 10, 11); H-9→C-(5, 7, 8, 11); H-12→C-(6, 11, 13, 14); H-13→C-(12, 14, 15); H-14→C-(12, 13, 15, 16); H-18→C-(16, 17, 19); H-19→C-(17, 18); H-21→C-(20, 22, 23); H-22→C-(20, 21, 23); H-23→C-(20, 21, 22).


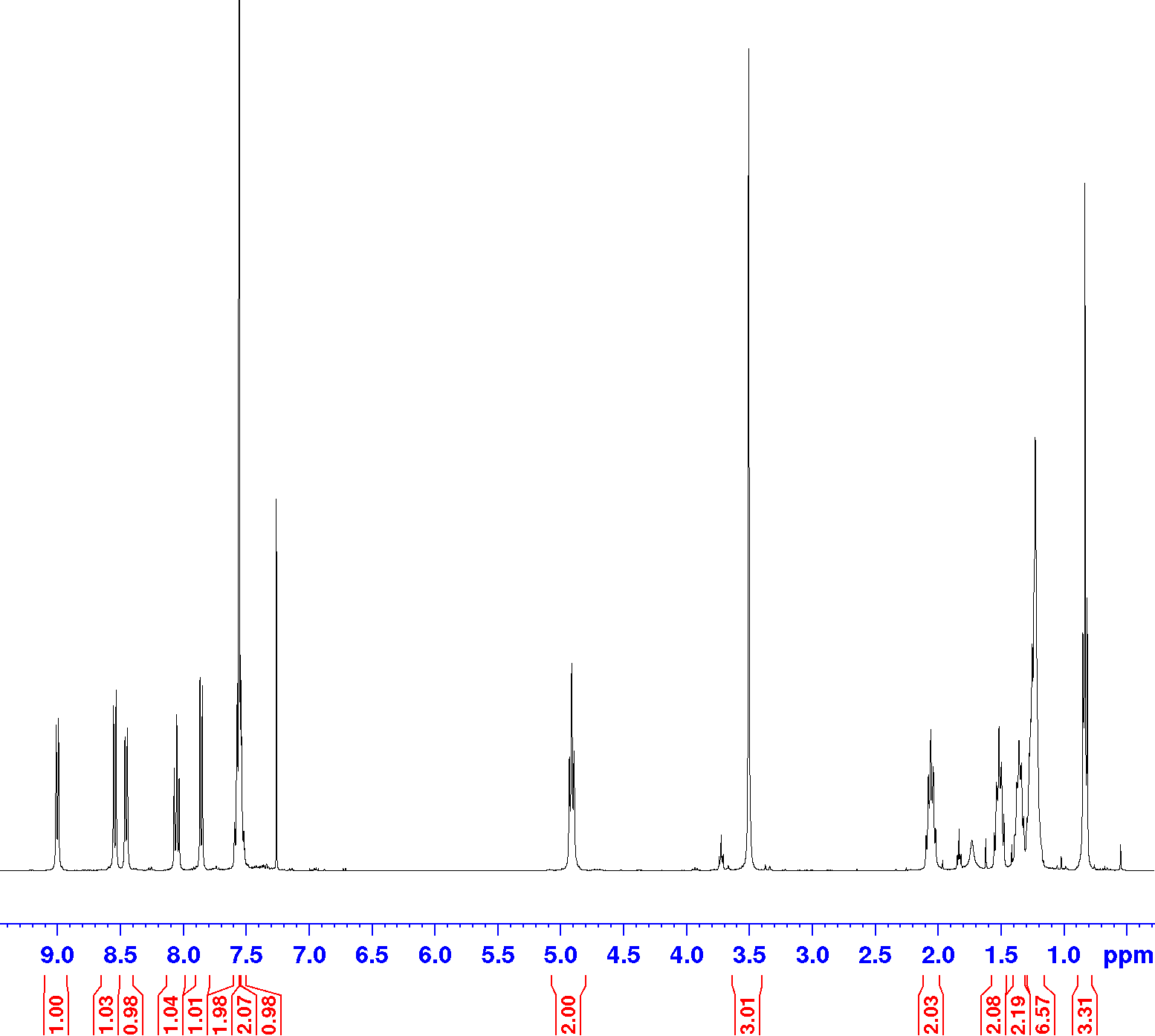


^1^H NMR (CDCl_3_, 400.2 MHz): *δ* 9.00 (d, *J* = 7.3, 1H, H-9); 8.54 (d, *J* = 8.2, 1H, H-7); 8.45 (d, *J* = 7.6, 1H, H-1); 8.05 (dd, *J* = 8.2, 7.3, 1H, H-8); 7.86 (d, *J* = 7.6, 1H, H-2); 7.56 (m, 2H, H-22); 7.55 (m, 2H, H-21); 7.55 (m, 1H, H-23); 4.91 (t, *J* = 7.5, 2H, H-12); 3.50 (s, 3H, H-24); 2.06 (m, *J* = 7.5, 2H, H-13); 1.51 (m, 2H, H-14); 1.35 (m, 2H, H-15); 1.2-1.3 (m, 6H, H-16, 17, 18); 0.83 (t, *J* = 7.0, 3H, H-19).

DQF-COSY correlations: H-1→H-(2); H-2→H-(1); H-7→H-(8); H-8→H-(7, 9); H-9→H-(8); H-12→H-(13); H-13→H-(12, 14); H-14→H-(13, 15); H-15→H-(14, 16); H-16→H-(15); H-18→H-(19); H-19→H-(18).


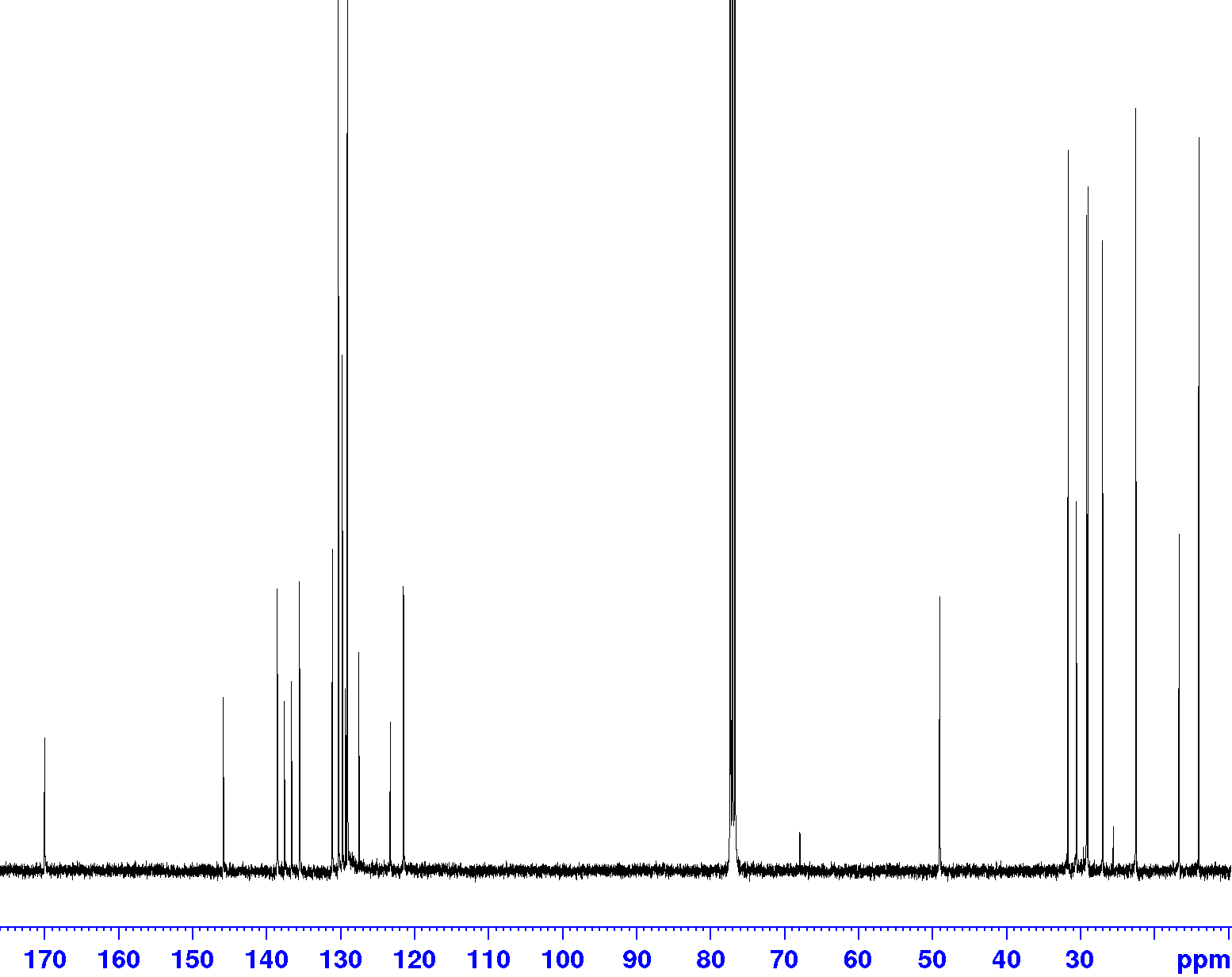


^13^C NMR (CDCl_3_, 100.6 MHz): *δ* 170.0 (s, C-11); 145.9 (s, C-3); 138.6 (d, C-7); 137.6 (s, C-6); 136.6 (s, C-20); 135.6 (d, C-9); 131.1 (d, C-8); 130.3 (d, C-21); 129.8 (d, C-2); 129.3 (s, C-10); 129.1 (d, C-23); 129.0 (d, C-22); 127.6 (s, C-4); 123.3 (s, C-5); 121.5 (d, C-1); 49.0 (t, C-12); 31.6 (t, C-17); 30.5 (t, C-13); 29.1 (t, C-15); 29.0 (t, C-16); 27.0 (t, C-14); 22.5 (t, C-18); 16.6 (q, C-24); 14.0 (q, C-19).

HMBC correlations: H-1→C-(3, 4w, 5, 6); H-2→C-(1, 4, 6, 20); H-7→C-(3, 5, 6, 8w, 9, 10w); H-8→C-(4, 7, 9, 10, 11); H-9→C-(5, 7, 8, 11); H-12→C-(6, 11, 13, 14); H-13→C-(12, 14, 15); H-14→C-(12, 13, 16); H-15→C-(13, 14, 16, 17); H-19→C-(17, 18); H-21→C-(3); H-22→C-(20, 22); H-24→C-(10, 11).


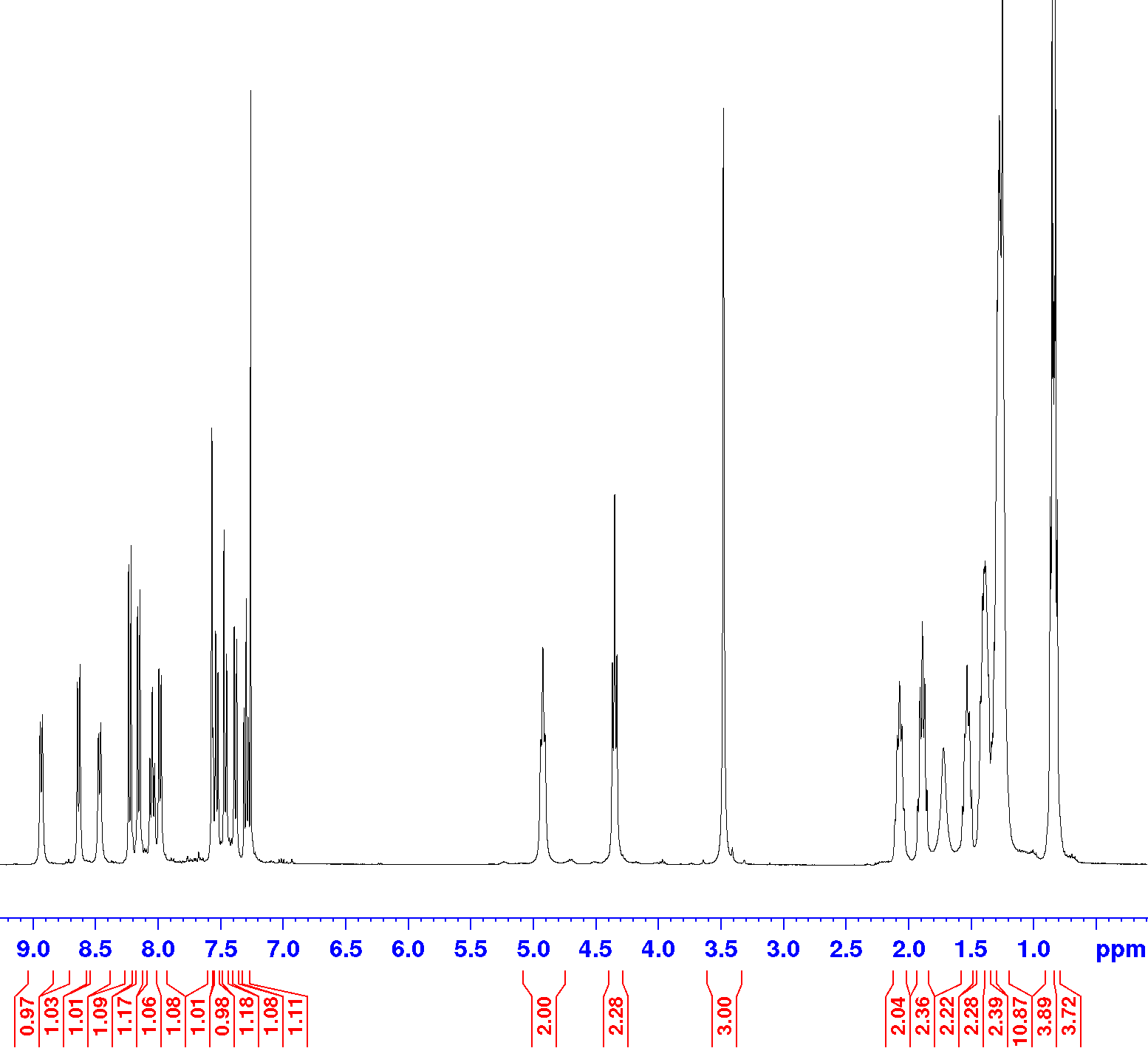


^1^H NMR (CDCl_3_, 400.2 MHz): *δ* 8.93 (d, *J* = 7.1, 1H, H-9); 8.64 (d, *J* = 8.2, 1H, H-7); 8.47 (d, *J* = 7.5, 1H, H-1); 8.23 (d, *J* = 8.0, 1H, H-23); 8.16 (m, 1H, H-26); 8.05 (dd, *J* = 8.2, 7.1, 1H, H-8); 7.99 (d, *J* = 7.5, 1H, H-2); 7.57 (d, *J* = 1.1, 1H, H-20); 7.54 (m, 1H, H-28); 7.46 (m, 1H, H-29); 7.38 (dd, *J* = 8.0/1.1, 1H, H-22); 7.30 (m, 1H, H-27); 4.92 (t (br), *J* = 6.9, 2H, H-12); 4.35 (t, *J* = 7.2, 2H, H-32); 3.49 (s, 3H, H-38); 2.07 (m, 2H, H-13); 1.89 (m, 2H, H-33); 1.53 (m, 2H, H-14); 1.40 (m, 2H, H-34); 1.37 (m, 2H, H-15); 1.2-1.3 (m, 10H, H-16, 17, 18, 35, 36); 0.85 (t, *J* = 7.0, 3H, H-19); 0.83 (t, *J* = 6.9, 3H, H-37).

DQF-COSY correlations: H-1→H-(2); H-2→H-(1); H-7→H-(8); H-8→H-(7, 9); H-9→H-(8); H-12→H-(13); H-13→H-(12, 14); H-14→H-(13, 15); H-15→H-(14, 16); H-16→H-(15); H-18→H-(19); H-19→H-(18); H-20→H-(22); H-22→H-(20, 23); H-23→H-(22); H-26→H-(27); H-27→H-(26, 28); H-28→H-(27, 29); H-29→H-(28); H-32→H-(33); H-33→H-(32, 34); H-34→H-(33, 35); H-35→H-(34); H-36→H-(37); H-37→H-(36).


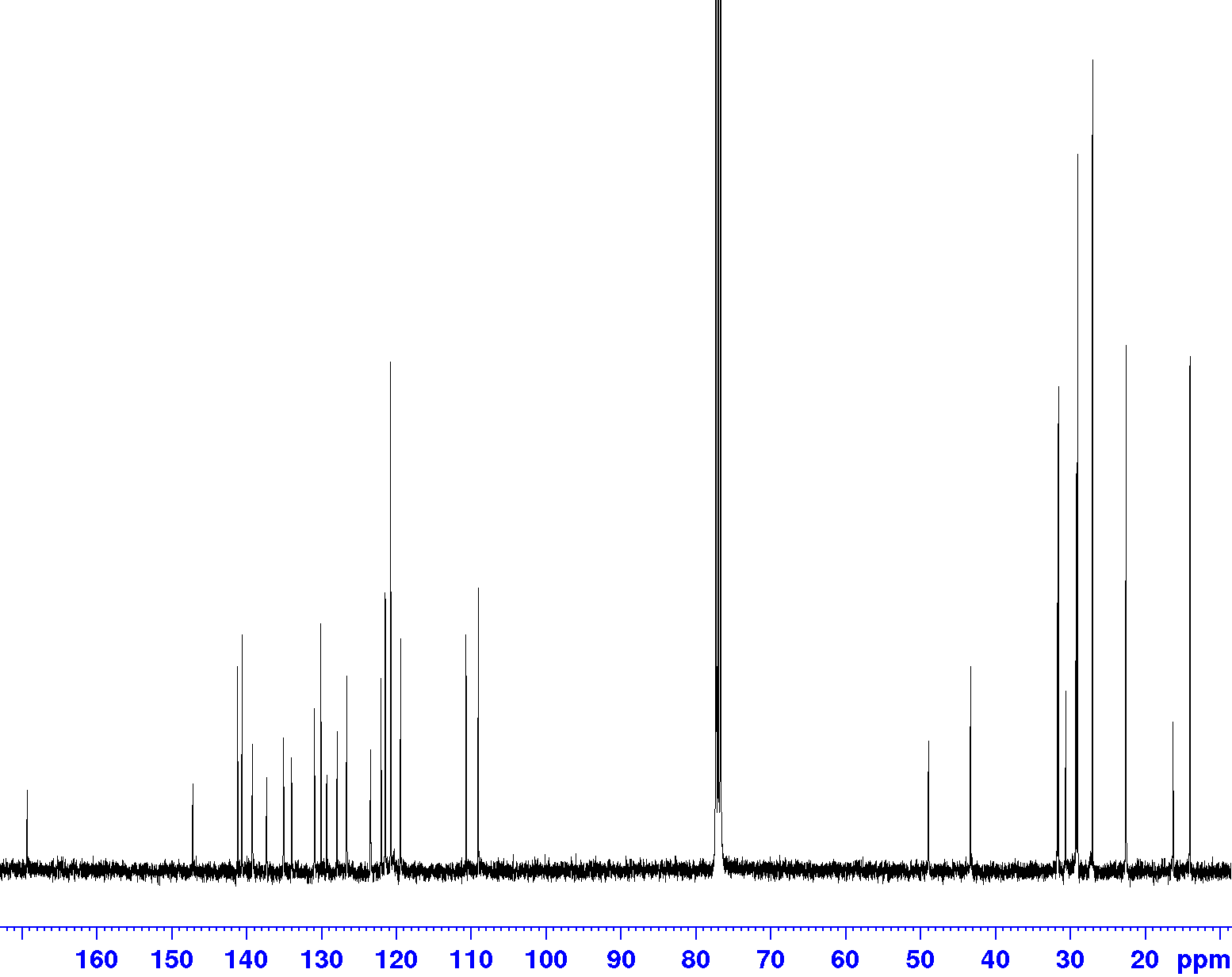


^13^C NMR (CDCl_3_, 100.6 MHz): *δ* 169.3 (s, C-11); 147.2 (s, C-3); 141.2 (s, C-30); 140.6 (s, C-31); 139.2 (d, C-7); 137.3 (s, C-6); 135.1 (d, C-9); 134 (s, C-21); 130.9 (d, C-8); 130.1 (d, C-2); 129.3 (s, C-10); 127.9 (s, C-4); 126.6 (d, C-28); 123.5 (s, C-24); 123.4 (s, C-5); 122.1 (s, C-25); 121.5 (d, C-22); 121.5 (d, C-1); 120.8 (d, C-26); 120.8 (d, C-23); 119.4 (d, C-27); 110.7 (d, C-20); 109.0 (d, C-29); 48.9 (t, C-12); 43.3 (t, C-32); 31.7 (t, C-17); 31.5 (t, C-35); 30.6 (t, C-13); 29.2 (t, C-15); 29.0 (t, C-16, 33); 27.0 (t, C-14, 34); 22.5 (t, C-18, 36); 16.3 (t, C-38); 14.0 (q, C-19, 37).

HMBC correlations: H-1→C-(3, 5, 6); H-2→C-(1, 4, 6, 21); H-7→C-(3, 5, 6w, 8, 9, 10w); H-8→C-(4, 7w, 9w, 10, 11w); H-9→C-(4w, 5, 7, 8w, 11); H-12→C-(6, 11, 13, 14); H-13→C-(12, 14, 15); H-14→C-(12, 13, 16); H-19→C-(17, 18); H-20→C-(3, 22, 24, 31w); H-22→C-(3, 20, 21w, 24); H-23→C-(21, 24w, 25, 31); H-26→C-(24, 25, 28, 30); H-27→C-(25, 28, 29); H-28→C-(26, 30); H-29→C-(25, 27); H-32→C-(30, 31, 33, 34); H-33→C-(32, 34, 35); H-34→C-(32, 33, 35, 36); H-37→C-(35, 36); H-38→C-(10, 11).


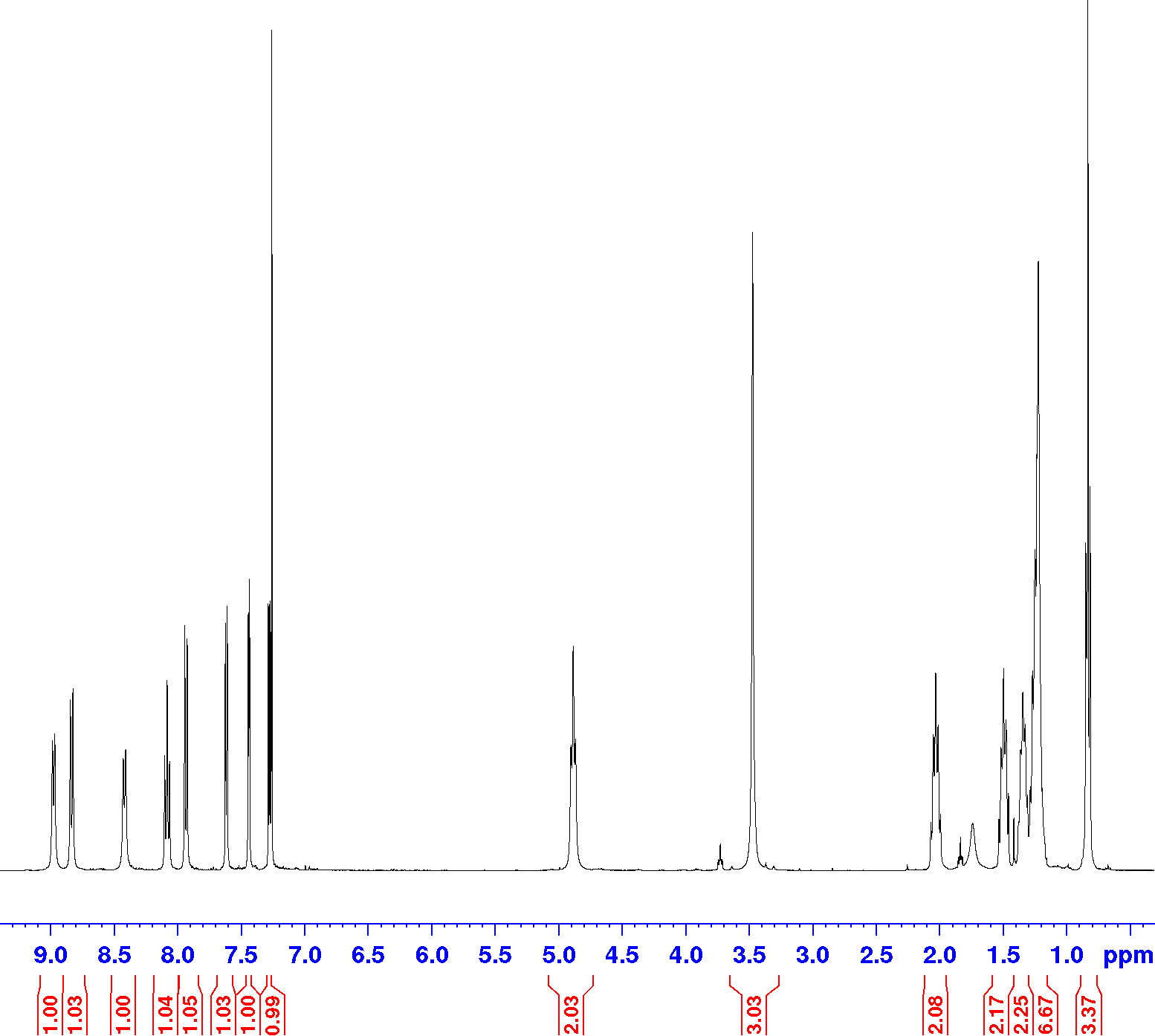


^1^H NMR (CDCl_3_, 400.2 MHz): *δ* 8.98 (d, *J* = 7.1, 1H, H-9); 8.84 (d, *J* = 8.2, 1H, H-7); 8.42 (d, *J* = 7.7, 1H, H-1); 8.09 (dd, *J* = 8.2, 7.1, 1H, H-8); 7.94 (d, *J* = 7.7, 1H, H-2); 7.62 (dd, *J* = 5.1, 0.8, 1H, H-23); 7.44 (dd, *J* = 3.7, 0.8, 1H, H-21); 7.28 (dd, *J* = 5.1, 3.7, 1H, H-22); 4.89 (t, *J* = 7.2, 2H, H-12); 3.47 (s, 3H, H-24); 2.03 (tt, *J* = 7.6, 7.2, 2H, H-13); 1.49 (m, 2H, H-14); 1.34 (m, 2H, H-15); 1.2-1.3 (m, 6H, H-16, 17, 18); 0.83 (t, *J* = 7.0, 3H, H-19).

DQF-COSY correlations: H-1→H-(2); H-2→H-(1); H-7→H-(8); H-8→H-(7, 9); H-9→H-(8); H-12→H-(13); H-13→H-(12, 14); H-14→H-(13, 15); H-15→H-(14, 16); H-16→H-(15); H-18→H-(19); H-19→H-(18); H-21→H-(22, 23w); H-22→H-(21, 23); H-23→H-(21w, 22).


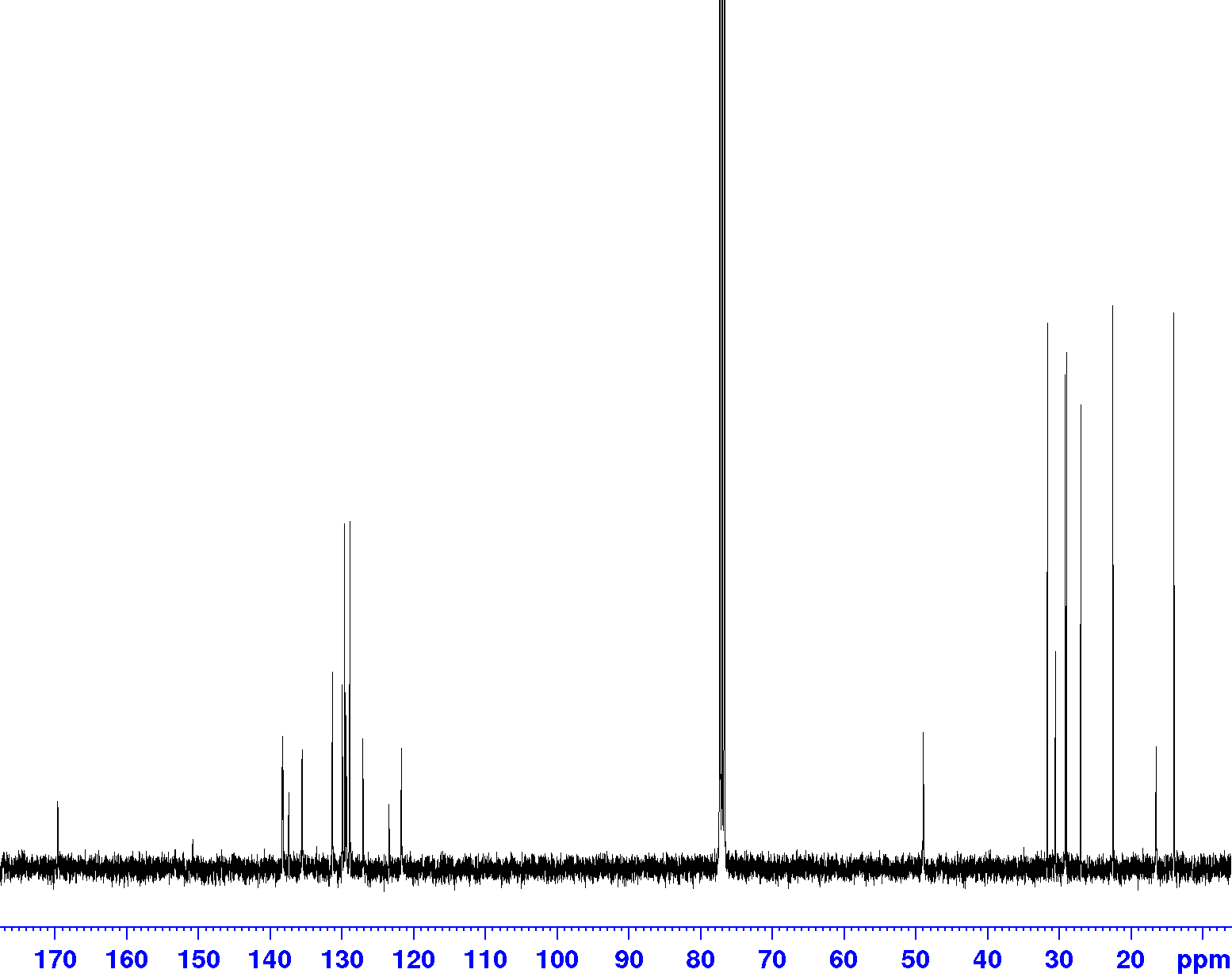


^13^C NMR (CDCl_3_, 100.6 MHz): *δ* 169.6 (s, C-11); 138.4 (s, C-20); 138.3 (d, C-7); 138.2 (s, C-3); 137.4 (s, C-6); 135.6 (d, C-9); 131.3 (d, C-8); 130.0 (d, C-21); 129.7 (d, C-2); 129.5 (d, C-23); 129.4 (s, C-10); 128.9 (d, C-22); 127.1 (s, C-4); 123.5 (s, C-5); 121.7 (d, C-1); 48.9 (t, C-12); 31.6 (t, C-17); 30.5 (t, C-13); 29.2 (t, C-15); 29.0 (t, C-16); 27.0 (t, C-14); 22.5 (t, C-18); 16.5 (q, C-24); 14.0 (q, C-19).

HMBC correlations: H-1→C-(3, 5, 6); H-2→C-(1w, 4, 6, 7w, 20); H-7→C-(3, 5, 6w, 9); H-8→C-(4, 7w, 9, 10, 11); H-9→C-(5, 7, 8w, 11); H-12→C-(6, 1, 13, 14); H-13→C-(12, 14, 15); H-14→C-(12, 13, 15, 16); H-15→C-(13, 14, 16); H-18→C-(17, 19); H-19→C-(17, 18); H-21→C-(20, 22, 23); H-22→C-(20, 21w, 23); H-23→C-(20, 21, 22); H-24→C-(10, 11).


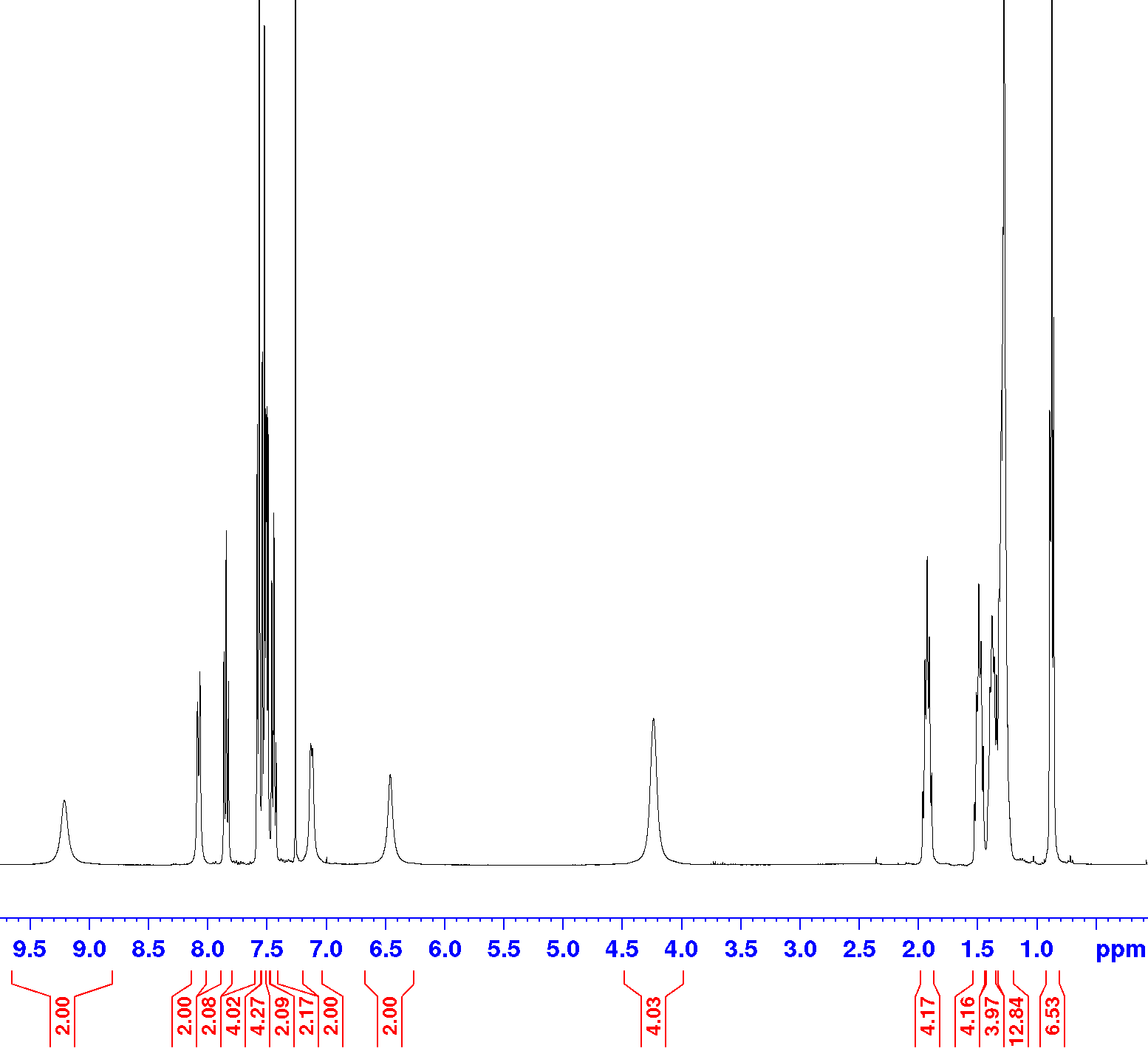


^1^H NMR (CDCl_3_, 400.2 MHz): *δ* 9.21 (s (br), 2H, H-9); 8.08 (d, *J* = 8.1, 2H, H-7); 7.84 (dd, *J* = 8.1/7.5, 2H, H-8); 7.57 (m, 4H, H-21); 7.52 (m, 4H, H-22); 7.50 (d, *J* = 7.4, 2H, H-2); 7.44 (m, 2H, H-23); 7.12 (d (br), 2H, H-1); 6.46 (s (br), 2H, H-24); 4.24 (m (br), 4H, H-12); 1.92 (m, 4H, H-13); 1.49 (m, 4H, H-14); 1.38 (m, 4H, H-15); 1.2-1.3 (m, 12H, H-16, 17, 18); 0.87 (t, *J* = 7.1, 6H, H-19).

DQF-COSY correlations: H-1→H-(2); H-2→H-(1); H-7→H-(8); H-8→H-(7, 9); H-9→H-(8); H-12→H-(13); H-13→H-(12, 14); H-14→H-(13, 15); H-15→H-(14); H-22→H-(21, 23); H-23→H-(22).

^
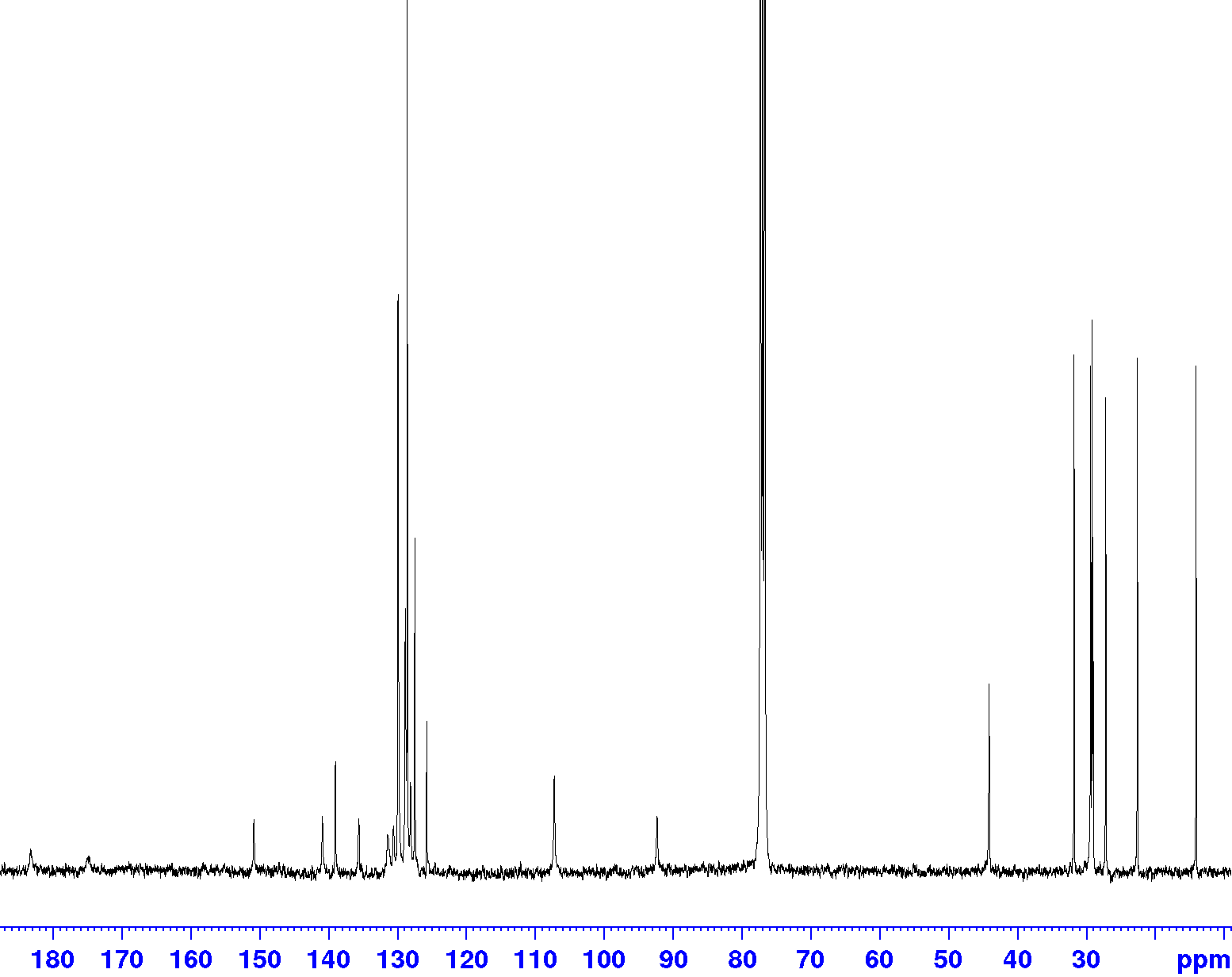
^

^13^C NMR (CDCl_3_, 100.6 MHz): *δ* 183.3 (s (br), C-26); 174.8 (s (br), C-25); 150.9 (s, C-11); 140.9 (s, C-6); 139.0 (s, C-20); 135.6 (s, C-3); 131.5 (d, C-9); 130.6 (s, C-10); 129.9 (s, C-21); 129.9 (d, C-8); 128.9 (d (br), C-7); 128.9 (d (br), C-2); 128.6 (d, C-22); 128.1 (s, C-4); 127.5 (d, C-23); 125.8 (s, C-5); 107.2 (d, C-1); 92.3 (d (br), C-24); 44.1 (t, C-12); 31.8 (t, C-17); 29.4 (t, C-15); 29.2 (t, C-16); 29.0 (t, C-13); 27.2 (t, C-14); 22.6 (t, C-18); 14.1 (q, C-19).

HMBC correlations: H-1→C-(3, 5); H-2→C-(1, 4, 6, 20); H-7→C-(3, 5, 8, 9); H-8→C-(4, 7,10, 11w); H-13→C-(12, 14, 15); H-14→C-(12, 13, 15, 16); H-15→C-(13, 14, 16); H-19→C-(17, 18); H-22→C-(20, 22); H-23→C-(21); no HMBC correlations observed for H-9, 12, 24.


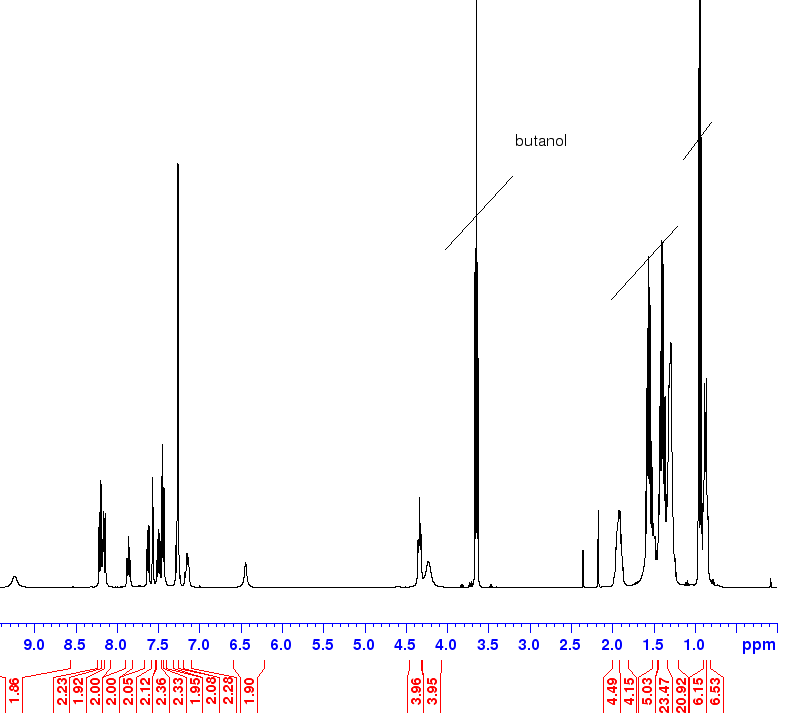


^1^H NMR (CDCl_3_, 400.2 MHz): *δ* 9.26 (s (br), 2H, H-9); 8.21 (d, *J* = 8.1, 2H, H-23); 8.19 (d, *J* = 7.8, 2H, H-7); 8.16 (m, 2H, H-26); 7.87 (dd, *J* = 7.8, 7.8, 2H, H-8); 7.63 (d, *J* = 7.5, 2H, H-2); 7.57 (s, 2H, H-20); 7.51 (m, 2H, H-28); 7.45 (m, 2H, H-29); 7.45 (d, *J* = 8.1, 2H, H-22); 7.29 (m, 2H, H-27); 7.15 (d (br), 2H, H-1); 6.44 (s (br), 2H, H-38); 4.34 (s (br), 4H, H-32); 4.24 (s (br), 4H, H-12); 1.94 (m, 4H, H-13); 1.91 (m, 4H, H-33); 1.51 (m, 4H, H-14); 1.42 (m, 4H, H-34); 1.39 (m, 4H, H-15); 1.25-1.35 (m, 20H, H-16, 17, 18, 36, 36); 0.89 (t, *J* = 7.0, 6H, H-19); 0.86 (t, *J* = 6.9, 6H, H-37).

DQF-COSY correlations: H-1→H-(2); H-2→H-(1); H-7→H-(8); H-8→H-(7, 9); H-9→H-(8); H-12→H-(13); H-13→H-(12, 14); H-14→H-(13, 15); H-15→H-(14); H-18→H-(19); H-19→H-(18); H-20→H-(22w); H-22→H-(20w, 23); H-23→H-(22); H-26→H-(27); H-27→H-(26, 28); H-28→H-(27, 29); H-29→H-(28); H-32→H-(33); H-33→H-(32, 34); H-34→H-(33, 35); H-35→H-(34); H-36→H-(37); H-37→H-(36).

^
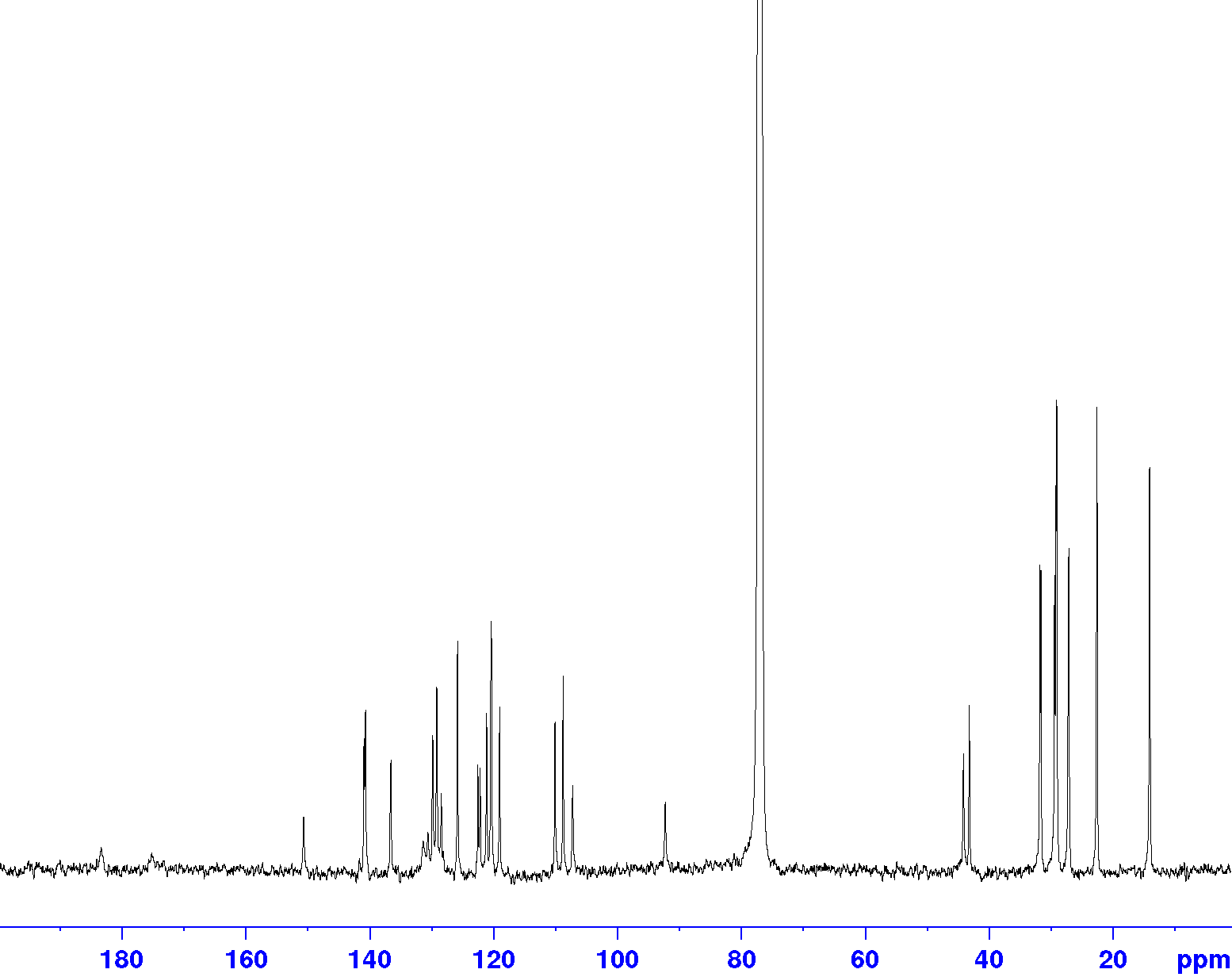
^

^13^C NMR (CDCl_3_, 100.6 MHz): *δ* 183.4 (s (br), C-40); 175.2 (s (br), C-39); 150.7 (s, C-11); 141.0 (s, C-30); 140.8 (s, C-6); 140.7 (s, C-31); 136.7 (s, C-3); 136.6 (s, C-21); 131.4 (d, C-9); 130.7 (s, C-10); 129.9 (d, C-8); 129.2 (d, C-7); 129.2 (d, C-2); 128.5 (s, C-4); 125.8 (s, C-5); 125.8 (d, C-28); 122.6 (s, C-25); 122.2 (s, C-24); 121.2 (d, C-22); 120.5 (d, C-26); 120.4 (d, C-23); 119.0 (d, C-27); 110.1 (d, C-20); 108.8 (d, C-29); 107.2 (d, C-1); 92.3 (d, C-38); 44.1 (t, C-12); 43.2 (t, C-32); 31.8 (t, C-17); 31.6 (t, C-35); 29.4 (t, C-15); 29.2 (t, C-16); 29.1 (t, C-13, 33); 27.2 (t, C-14); 27.1 (t, C-34); 22.6 (t, C-18, 36); 14.0 (q, 19, C-37).

HMBC correlations: H-1→C-(3, 5, 6w); H-2→C-(1w, 4, 6, 21); H-7→C-(3, 5, 9); H-8→C-(4, 9, 10, 11w); H-13→C-(12, 14, 15); H-14→C-(12, 15, 16); H-19→C-(17, 18); H-20→C-(3, 22, 24); H-22→C-(3, 20, 24); H-23→C-(20w, 21, 24, 25, 31); H-26→C-(24, 25w, 28, 30); H-27→C-(25, 28, 29); H-28→C-(26, 30); H-29→C-(25, 27); H-32→C-(30, 31, 33, 34); H-33→C-(32, 34, 35); H-34→C-(32, 33, 35, 36); H-37→C-(35, 36); no HMBC correlations observed for H-9, 12, 38.

^
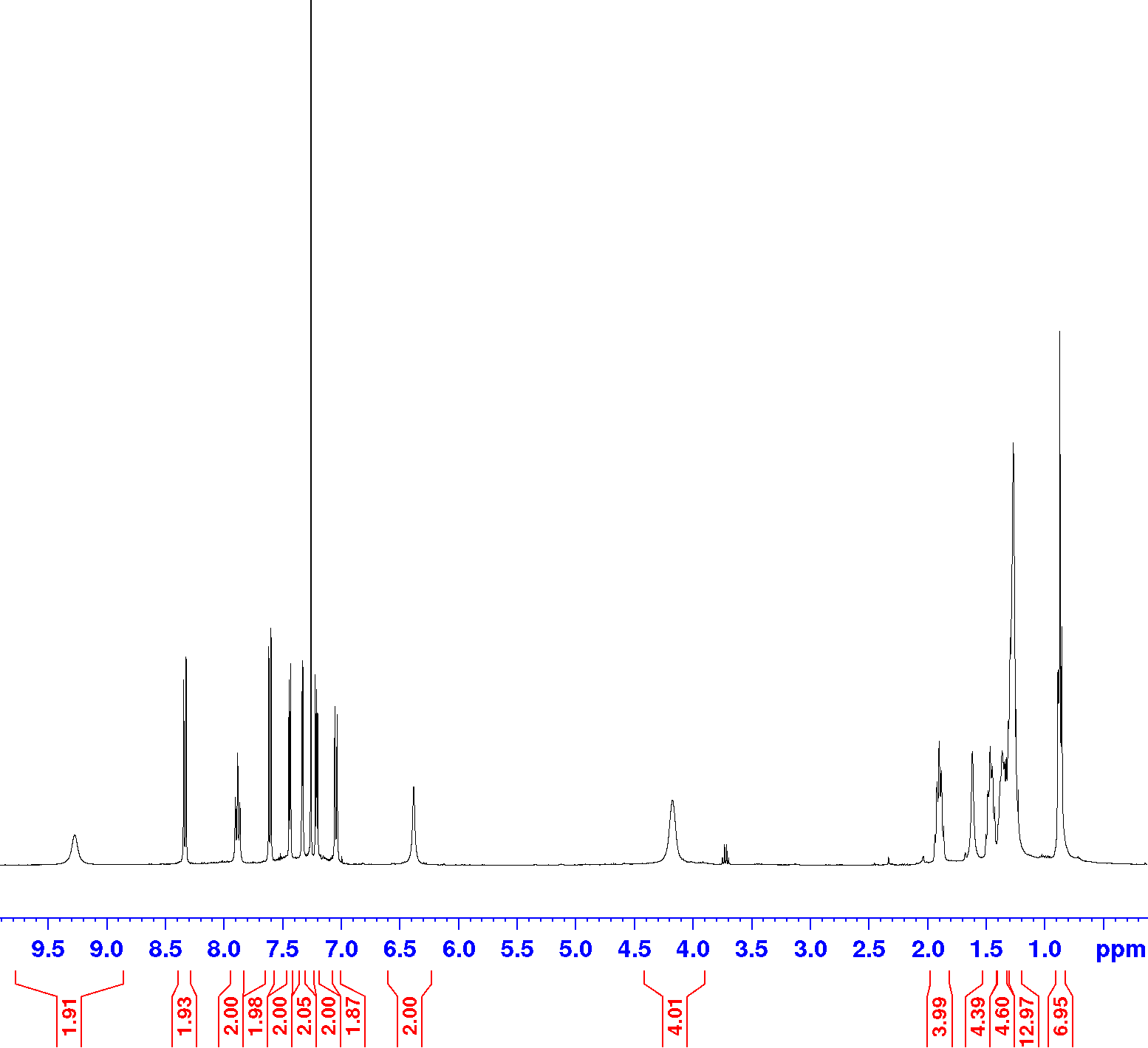
^

^1^H NMR (CDCl_3_, 400.2 MHz): *δ* 9.24 (s (br), 2H, H-9); 8.33 (d, *J* = 8, 2H, H-7); 7.88 (dd, *J* = 8.0, 8.0, 2H, H-8); 7.61 (d, *J* = 7.6, 2H, H-2); 7.44 (d, *J* = 5, 2H, H-23); 7.33 (d, *J* = 3.7, 2H, H-21); 7.21 (dd, *J* = 5.0, 3.7, 2H, H-22); 7.04 (d, *J* = 7.6, 2H, H-1); 6.40 (s (br), 2H, H-24); 4.18 (m (br), 4H, H-12); 1.89 (m, 4H, H-13); 1.46 (m, 4H, H-14); 1.35 (m, 4H, H-15); 1.2-1.3 (m, 12H, H-16, 17, 18); 0.87 (t, *J* = 6.9, 6H, H-19).

DQF-COSY correlations: H-1→H-(2); H-2→H-(1); H-7→H-(8); H-8→H-(7, 9); H-9→H-(8); H-12→H-(13); H-13→H-(12, 14); H-14→H-(13, 15); H-15→H-(14); H-18→H-(19); H-19→H-(18); H-21→H-(22, 23w); H-22→H-(21, 23); H-23→H-(21w, 22).


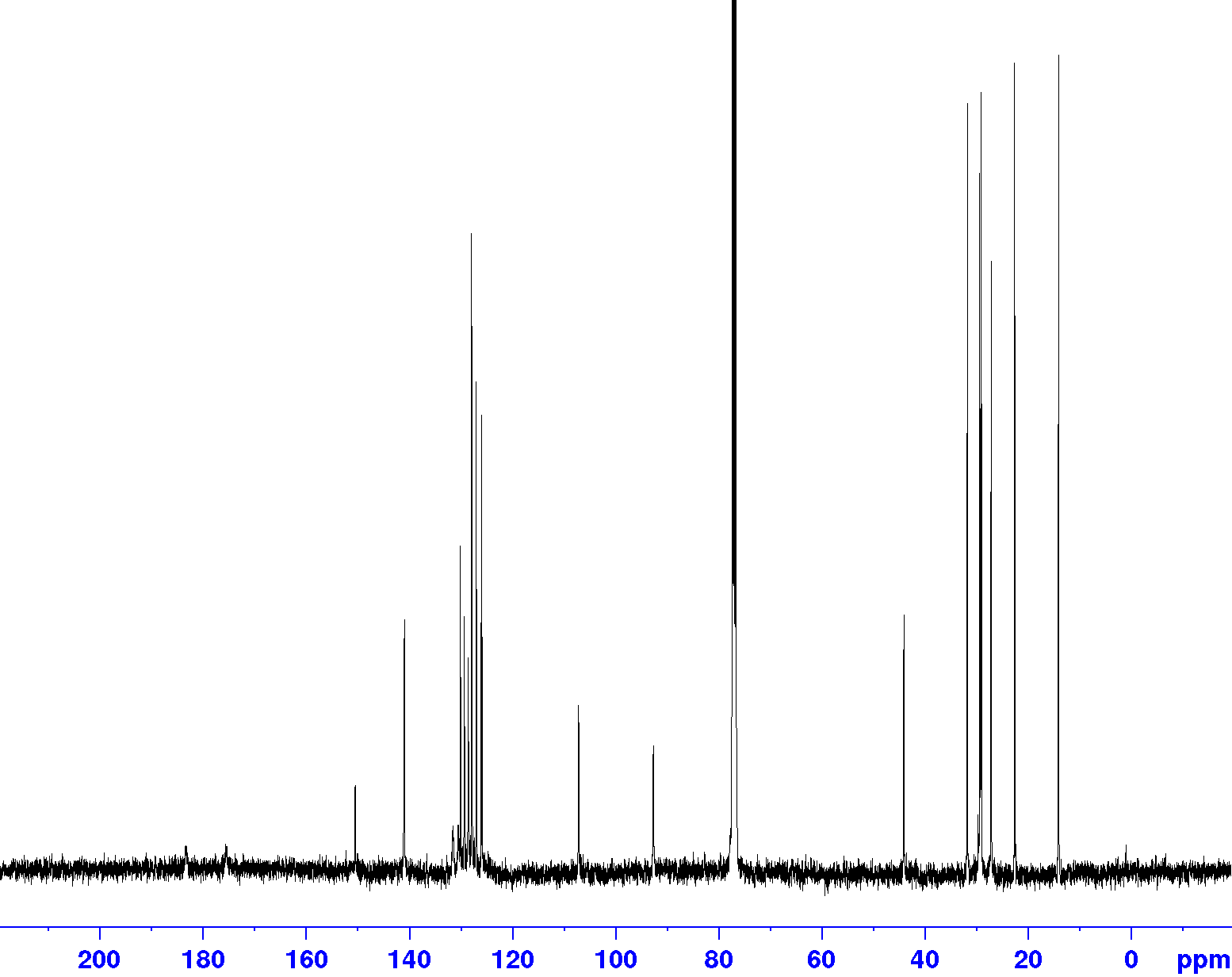


^13^C NMR (CDCl_3_, 100.6 MHz): *δ* 183.3 (s (br), C-26); 175.6 (s (br), C-25); 150.5 (s, C-11); 141.1 (s, C-6); 141.0 (s, C-20); 131.6 (d, C-9); 130.5 (s (br), C-10); 130.1 (d, C-8); 129.4 (d, C-2); 128.6 (d, C-7); 127.9 (s, C-3); 127.9 (d, C-22); 127.8 (s (br), C-4); 127.0 (d, C-21); 126.0 (d, C-23); 125.8 (s, C-5); 107.2 (d, C-1); 92.7 (d, C-24); 44.1 (t, C-12); 31.8 (t, C-17); 29.4 (t, C-15); 29.2 (t, C-16); 29.0 (t, C-13); 27.2 (t, C-14); 22.6 (t, C-18); 14.1 (q, C-19).

HMBC correlations: H-1→C-(3, 5, 6); H-2→C-(1w, 4, 6, 20); H-7→C-(3, 5, 6w, 9); H-8→C-(4, 7w, 9, 10, 11); H-9→C-(5, 7, 8w, 11); H-13→C-(12, 14, 15); H-14→C-(12, 13, 15, 16); H-15→C-(13, 14, 16, 17w); H-19→C-(17, 18); H-21→C-(20, 22, 23); H-22→C-(20, 21w); H-23→C-(20, 21, 22); H-24→C-(10, 11, 26).

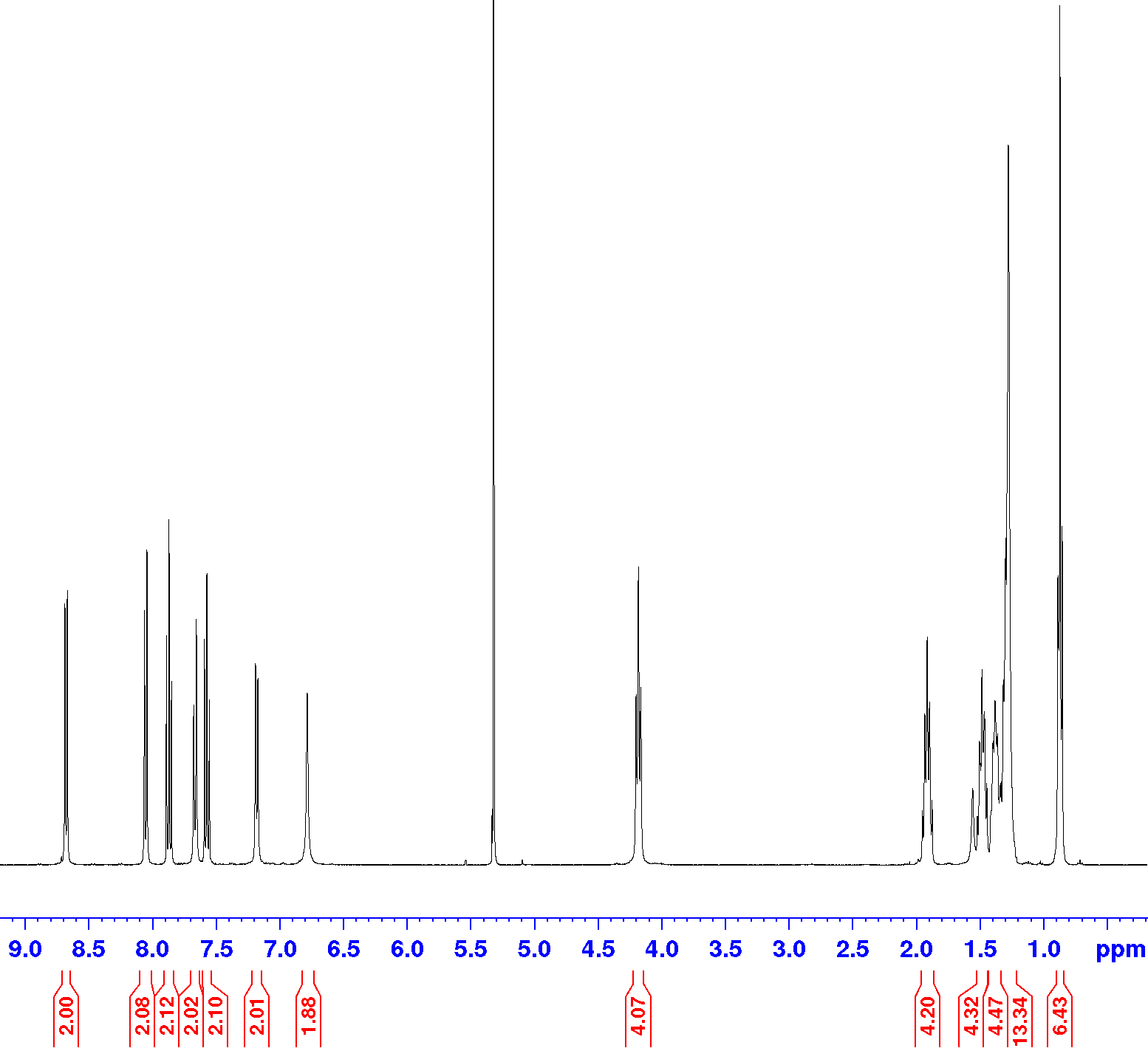
^1^H

^1^NMR (CD_2_Cl_2_, 400.2 MHz): *δ* 8.68 (d, *J* = 7.4, 2H, H-9); 8.05 (d, *J* = 8.1, 2H, H-7); 7.87 (dd, *J* = 8.1, 7.4, 2H, H-8); 7.66 (d, *J* = 8.3, 2H, H-3); 7.57 (dd, *J* = 8.3, 7.3, 2H, H-2); 7.18 (d, *J* = 7.3, 2H, H-1); 6.78 (s, 2H, H-20); 4.18 (t, *J* = 7.4, 4H, H-12); 1.91 (m, 4H, H-13); 1.48 (m, 4H, H-14); 1.38 (m, 4H, H-15); 1.3-1.2 (m, 12H, H-16, 17, 18); 0.87 (t, *J* = 6.8, 6H, H-19).

DQF-COSY correlations: H-1→H-(2); H-2→H-(1, 3); H-3→H-(2); H-7→H-(8); H-8→H-(7, 9); H-9→H-(8); H-12→H-(13); H-13→H-(12, 14); H-14→H-(13, 15); H-15→H-(14, 16); H-16→H-(15); H-18→H-(19); H-19→H-(18).


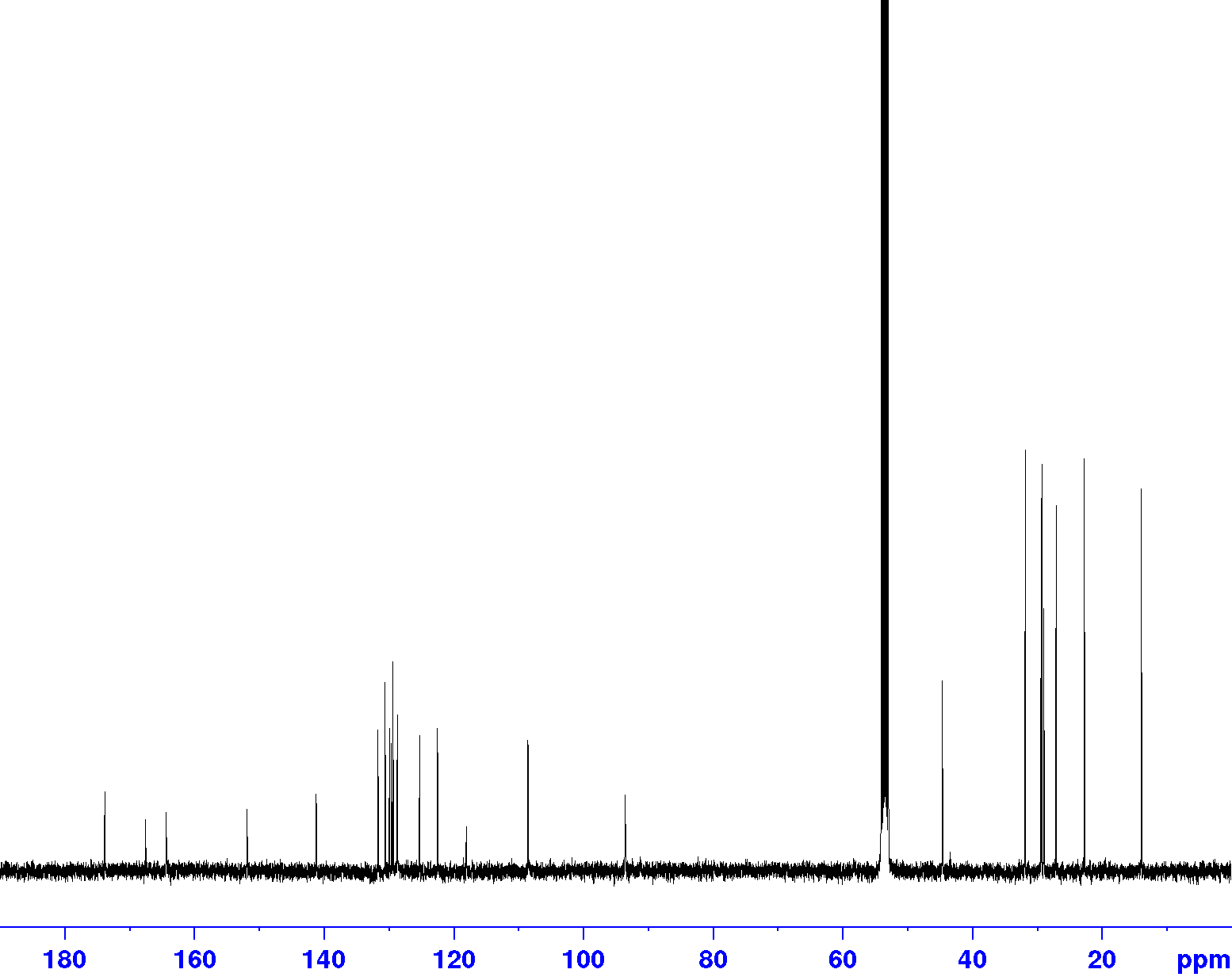


^13^C NMR (CD_2_Cl_2_, 100.6 MHz): *δ* 174.2, 167.9 (s, C-22, 23); 164.7 (s, C-21); 152.3 (s, C-11); 141.6 (s, C-6); 132.0 (d, C-9); 130.9 (d, C-7); 130.3 (s, C-10); 130.0 (s, C-4); 129.8 (d, C-8); 129.1 (d, C-2); 125.6 (s, C-5); 122.8 (d, C-3); 118.4 (s, C-25); 108.9 (d, C-1); 93.9 (d, C-20); 45.0 (t, C-12); 43.7 (s, C-24); 32.2 (t, C-17); 29.7 (t, C-15); 29.6 (t, C-16); 29.3 (t, C-13); 27.4 (t, C-14); 23.0 (t, C-18); 14.3 (q, C-19).

HMBC correlations: H-1→C-(3, 4w, 5, 6); H-2→C-(1, 4, 6); H-3→C-(1, 4w, 5, 7); H-7→C-(3, 4w, 5, 9); H-8→C-(4, 9w, 10, 11w); H-9→C-(5, 7, 8, 11); H-12→C-(6, 11, 13, 14); H-13→C-(12, 14, 15); H-14→C-(12, 13, 15, 16); H-15→C-(14, 16); H-19→C-(17, 18); H-20→C-(10, 11, 21, 22, 23).

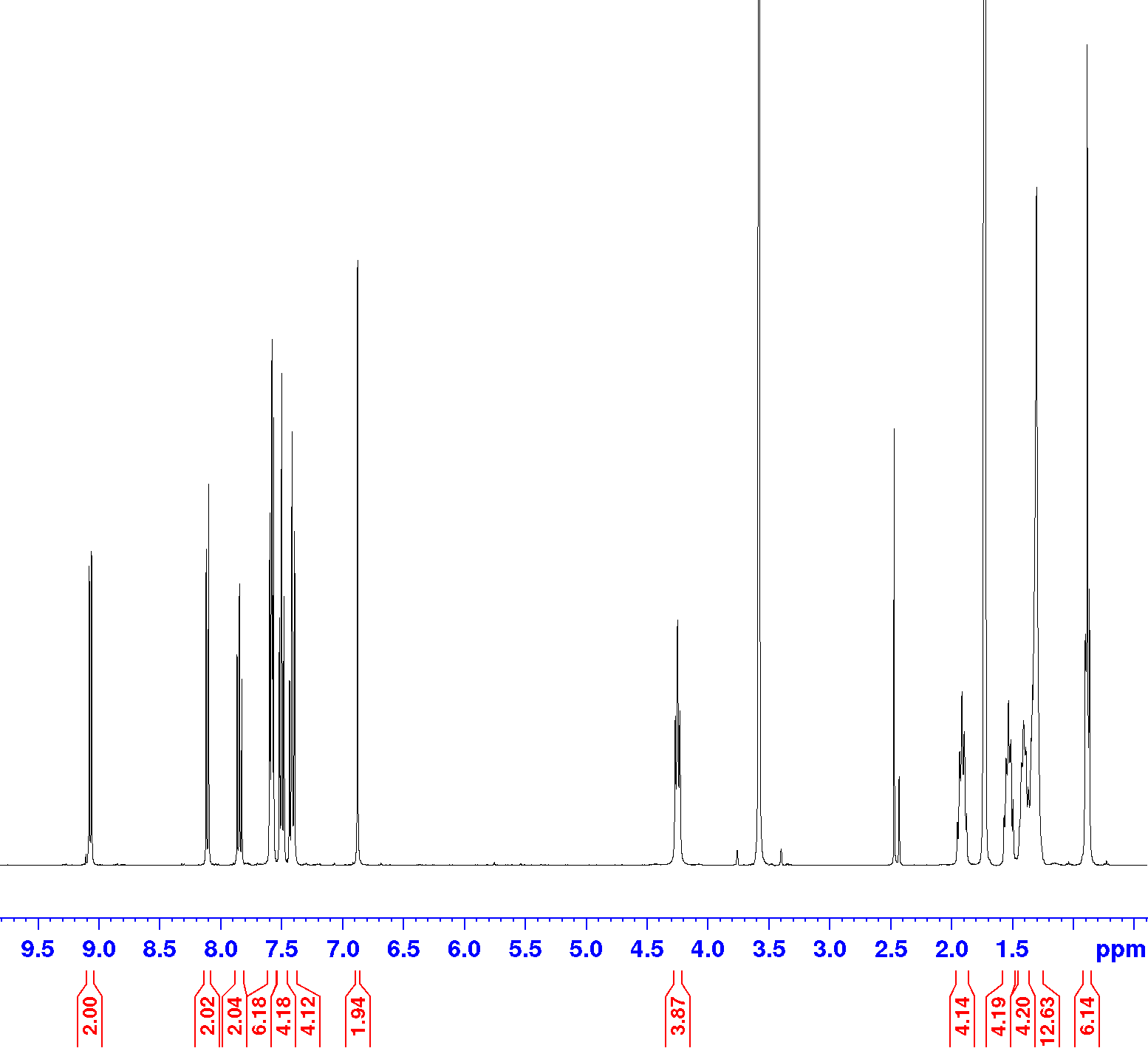


^1^H NMR (THF-d_8_, 400.2 MHz): *δ* 9.07 (d, *J* = 7.4, 2H, H-9); 8.11 (d, *J* = 8.2, 2H, H-7); 7.84 (dd, *J* = 8.2, 7.4, 2H, H-8); 7.59 (m, 4H, H-21); 7.58 (d, *J* = 7.6, 2H, H-2); 7.50 (m, 4H, H-22); 7.42 (m, 2H, H-23); 7.42 (d, *J* = 7.6, 2H, H-1); 6.88 (s, 2H, H-24); 4.25 (t, *J* = 7.5, 4H, H-12); 1.91 (m, 4H, H-13); 1.53 (m, 4H, H-14); 1.40 (m, 4H, H-15); 1.3-1.2 (m, 12H, H-16, 17, 18); 0.88 (t, *J* = 6.8, 6H, H-19).

DQF-COSY correlations: H-1→H-(2); H-2→H-(1); H-7→H-(8); H-8→H-(7, 9); H-9→H-(8); H-12→H-(13); H-13→H-(12, 14); H-14→H-(13, 15); H-15→H-(14, 16); H-16→H-(15); H-18→H-(19); H-19→H-(18); H-21→H-(22); H-22→H-(21, 23); H-23→H-(22).

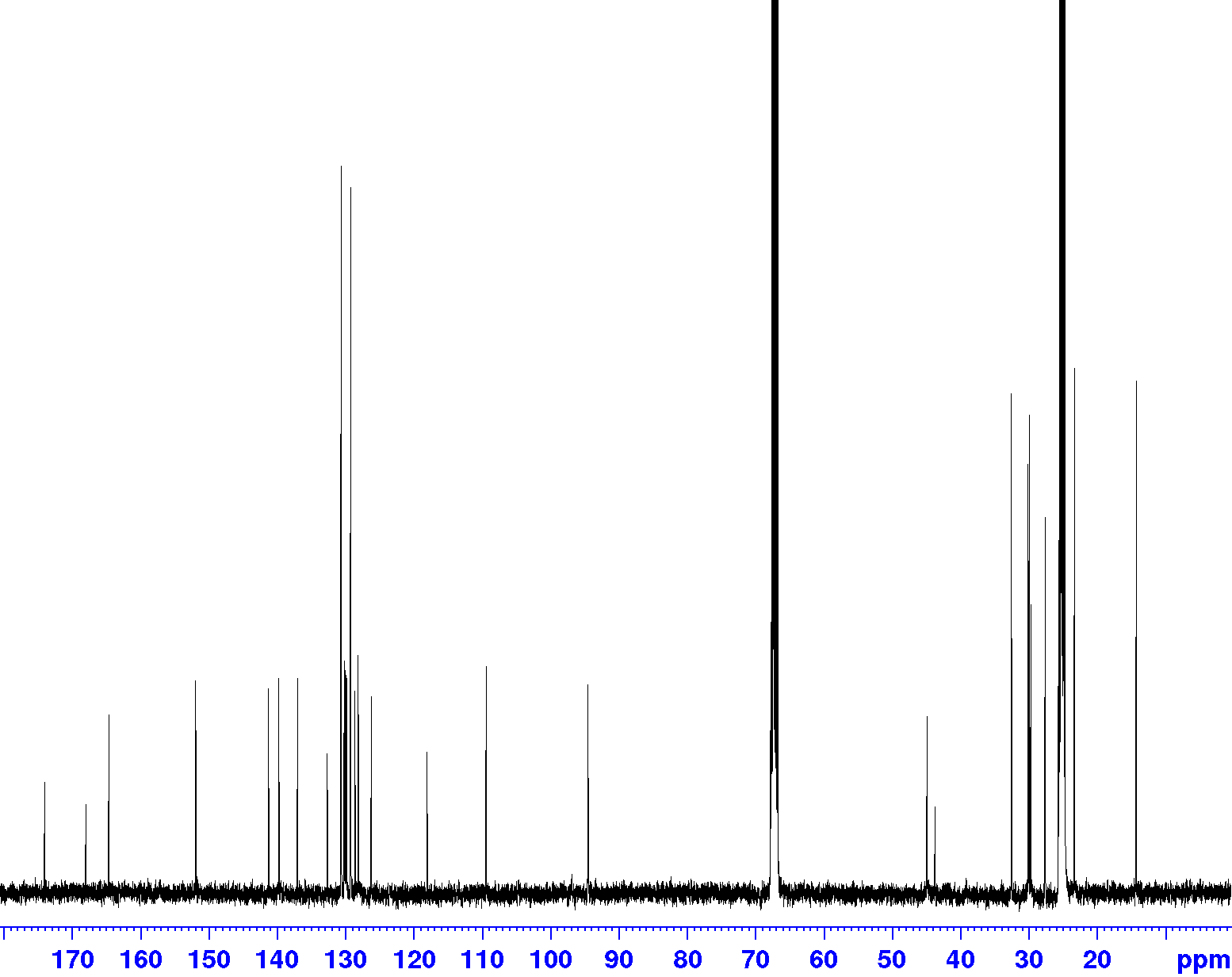


^13^C NMR (THF-d_8_, 100.6 MHz): *δ* 174.1, 168.1 (s, C-26, 27); 164.7 (s, C-25); 152.0 (s, C-11); 141.3 (s, C-6); 139.8 (s, C-20); 137.1 (s, C-3); 132.7 (d, C-9); 130.7 (s, C-10); 130.7 (d, C-21); 130.2 (d, C-8); 130.0 (d, C-7); 129.9 (d, C-2); 129.3 (d, C-22); 128.7 (s, C-4); 128.2 (d, C-23); 126.3 (s, C-5); 118.1 (s, C-29); 109.5 (d, C-1); 94.6 (d, C-24); 44.9 (t, C-12); 43.7 (s, C-28); 32.6 (t, C-17); 30.1 (t, C-15); 30.0 (t, C-16); 29.7 (t, C-13); 27.6 (t, C-14); 23.4 (t, C-18); 14.3 (q, C-19).

HMBC correlations: H-1→C-(3, 5, 6w); H-2→C-(4, 6, 20); H-7→C-(3, 5, 9); H-8→C-(4, 9w, 10, 11w); H-9→C-(5, 7, 11); H-12→C-(6, 11, 13, 14); H-13→C-(12, 14, 15); H-14→C-(13, 15, 16); H-15→C-(16); H-19→C-(17, 18); H-21→C-(3, 21, 23); H-22→C-(20, 22); H-23→C-(21); H-24→C-(10, 11, 25, 26, 27).

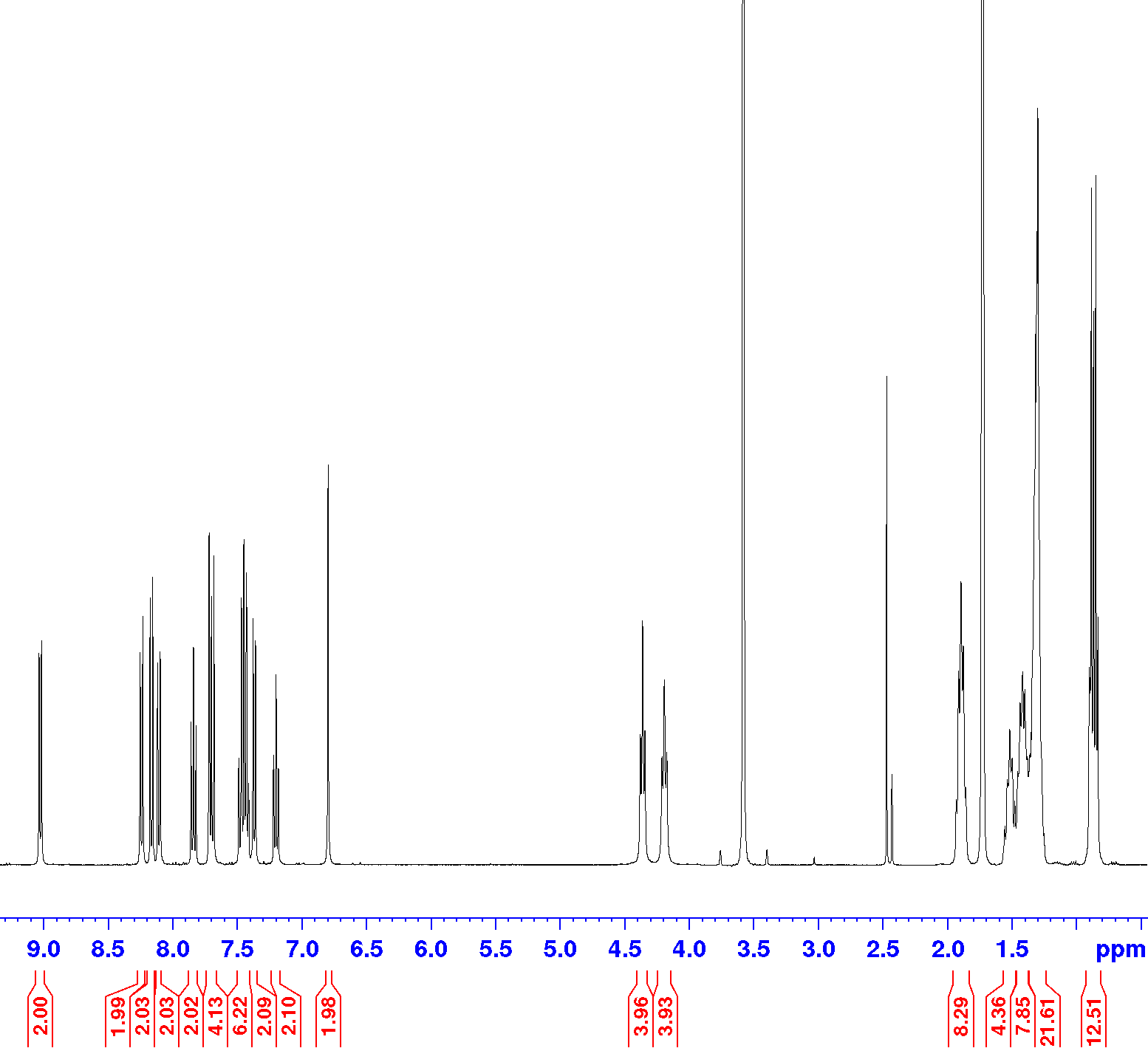


^1^H NMR (THF-d_8_, 400.2 MHz): *δ* 9.03 (d, *J* = 7.3, 2H, H-9); 8.24 (d, *J* = 8.3, 2H, H-7); 8.17 (d, *J* = 8, 2H, H-23); 8.11 (d, *J* = 7.7, 2H, H-26); 7.84 (dd, *J* = 8.3, 7.3, 2H, H-8); 7.72 (s(br), 2H, H-20); 7.69 (d, *J* = 7.6, 2H, H-2); 7.48 (m, 2H, H-29); 7.44 (d, *J* = 8, 2H, H-22); 7.43 (m, 2H, H-28); 7.37 (d, *J* = 7.6, 2H, H-1); 7.20 (m, 2H, H-27); 6.80 (s, 2H, H-38); 4.36 (t, *J* = 7.1, 4H, H-32); 4.19 (t, *J* = 7.4, 4H, H-12); 1.89 (m, 8H, H-13, 33); 1.53 (m, 8H, H-14, 34); 1.42 (m, 4H, H-15); 1.3-1.2 (m, 20H, H-16, 17, 18, 35, 36); 0.88 (t, *J* = 6.8, 6H, H-19); 0.84 (t, *J* = 6.8, 6H, H-37).

DQF-COSY correlations: H-1→H-(2); H-2→H-(1); H-7→H-(8); H-8→H-(7, 9); H-9→H-(8); H-12→H-(13); H-13→H-(12, 14); H-14→H-(13); H-18→H-(19); H-19→H-(18); H-20→H-(22); H-22→H-(20, 23); H-23→H-(22); H-26→H-(27); H-27→H-(26, 28); H-28→H-(27, 29); H-29→H-(28); H-32→H-(33); H-33→H-(32, 34); H-34→H-(33); H-36→H-(37); H-37→H-(36).

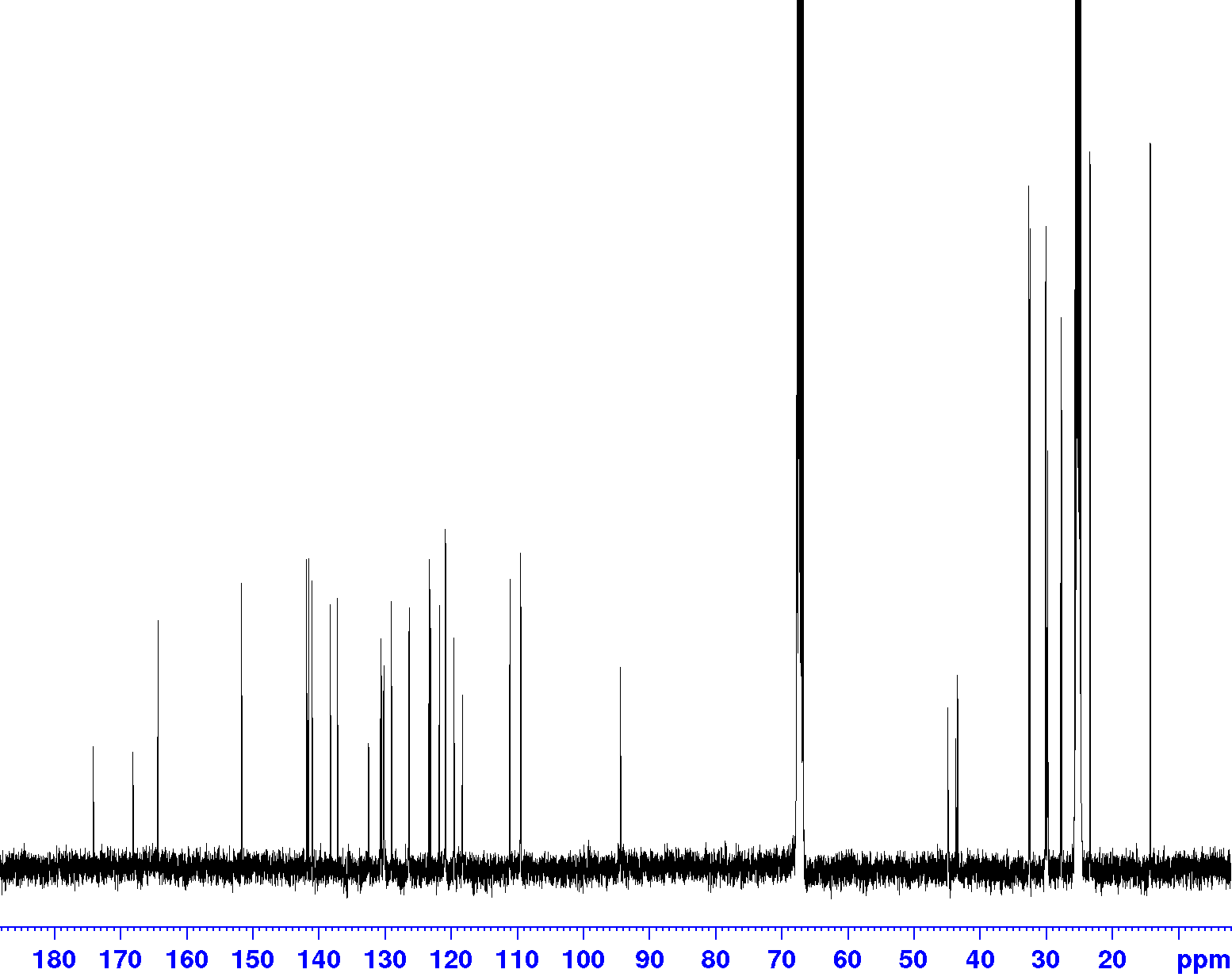


^13^C NMR (THF-d_8_, 100.6 MHz): *δ* 174.2, 168.1 (s, C-40, 41); 164.4 (s, C-39); 151.7 (s, C-11); 141.9 (s, C-30); 141.5 (s, C-31); 141.0 (s, C-6); 138.3 (s, C-3); 137.2 (s, C-21); 132.5 (d, C-9); 130.7 (s, C-10); 130.6 (d, C-7); 130.2 (d, C-2); 130.1 (d, C-8); 129.0 (s, C-4); 126.4 (d, C-28); 126.3 (s, C-5); 123.3 (s, C-25); 123.1 (s, C-24); 121.8 (d, C-22); 120.9 (d, C-26); 120.8 (d, C-23); 119.6 (d, C-27); 118.3 (s, C-43); 111.1 (d, C-20); 109.53 (d, C-29); 109.46 (d, C-1); 94.4 (d, C-38); 44.9 (t, C-12); 43.6 (s, C-42); 43.4 (t, C-32); 32.6 (t, C-17); 32.5 (t, C-35); 30.1 (t, C-15); 30.0 (t, C-16); 29.8 (t, C-33); 29.7 (t, C-13); 27.7 (t, C-34); 27.6 (t, C-14); 23.4 (t, C-18); 23.3 (t, C-36); 14.3 (q, C-19); 14.2 (q, C-37).

HMBC correlations: H-1→C-(3, 4w, 5, 6w); H-2→C-(1w, 4, 6, 21); H-7→C-(3, 5, 6w, 9); H-8→C-(4, 9w, 10); H-9→C-(5, 7, 11); H-12→C-(6, 11, 13, 14); H-13→C-(12, 14, 15); H-19→C-(17, 18); H-20→C-(3, 22, 24); H-22→C-(3, 20, 24); H-23→C-(21, 25, 31); H-26→C-(24, 28, 30); H-27→C-(25, 28w, 29); H-28→C-(26, 30); H-29→C-(25, 27); H-32→C-(30, 31, 33, 34); H-33→C-(32, 34, 35); H-37→C-(35, 36); H-38→C-(10, 11, 39, 40, 41).


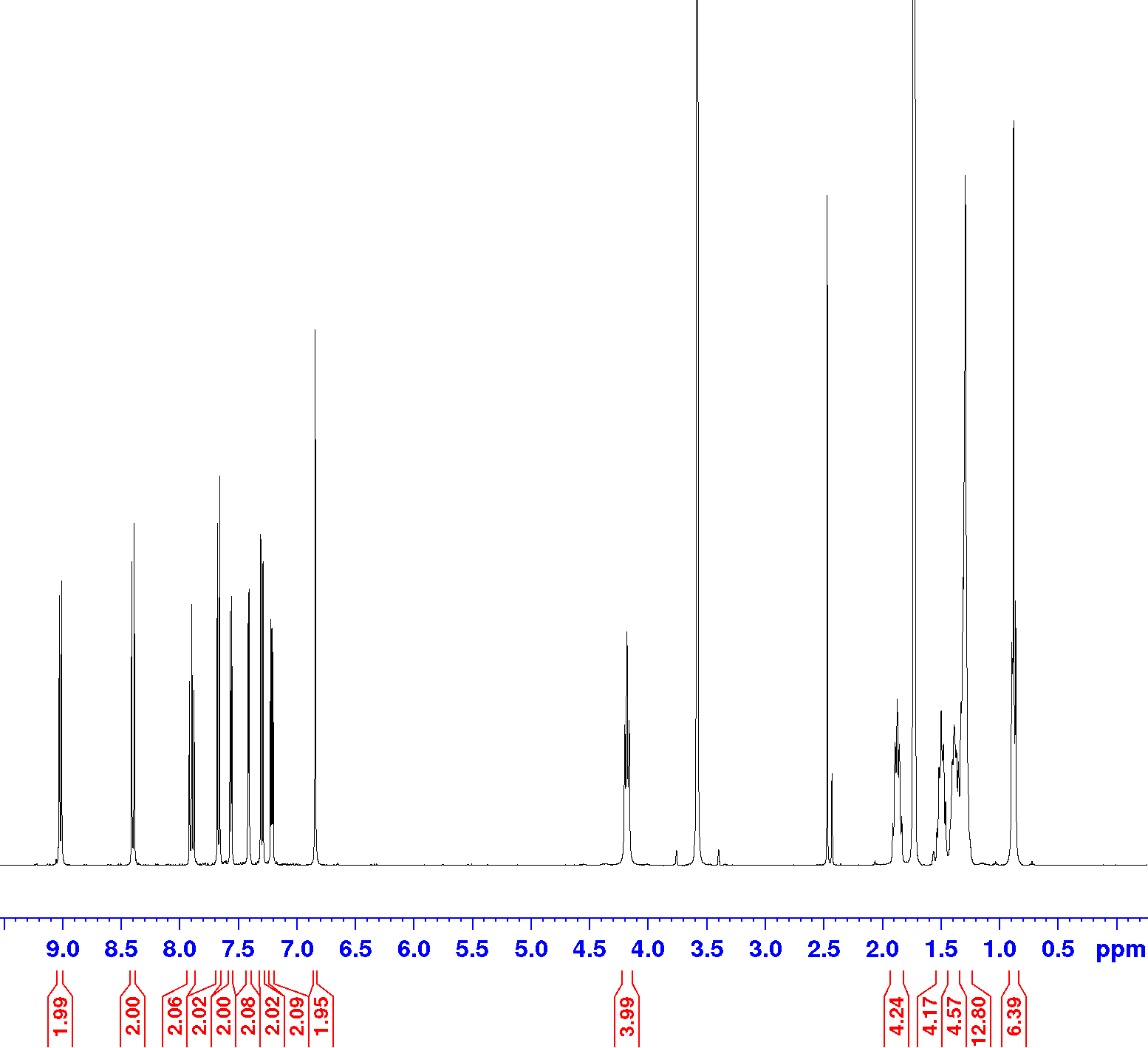


^1^H NMR (THF-d_8_, 400.2 MHz): *δ* 9.02 (d, *J* = 7.4, 2H, H-9); 8.40 (d, *J* = 8.3, 2H, H-7); 7.90 (dd, *J* = 8.3, 7.4, 2H, H-8); 7.67 (d, *J* = 7.7, 2H, H-2); 7.56 (dd, *J* = 5.1, 1.0, 2H, H-23); 7.41 (dd, *J* = 3.5, 1.0, 2H, H-21); 7.30 (d, *J* = 7.7, 2H, H-1); 7.21 (dd, *J* = 5.1, 3.5, 2H, H-22); 6.84 (s, 2H, H-24); 4.18 (t, *J* = 7.5, 4H, H-12); 1.87 (m, 4H, H-13); 1.50 (m, 4H, H-14); 1.39 (m, 4H, H-15); 1.3-1.2 (m, 12H, H-16, 17, 18); 0.88 (t, *J* = 6.8, 6H, H-19).

DQF-COSY correlations: H-1→H-(2); H-2→H-(1); H-7→H-(8); H-8→H-(7, 9); H-9→H-(8); H-12→H-(13); H-13→H-(12, 14); H-14→H-(13, 15); H-15→H-(14, 16); H-16→H-(15); H-18→H-(19); H-19→H-(18); H-21→H-(22); H-22→H-(21, 23); H-23→H-(22).


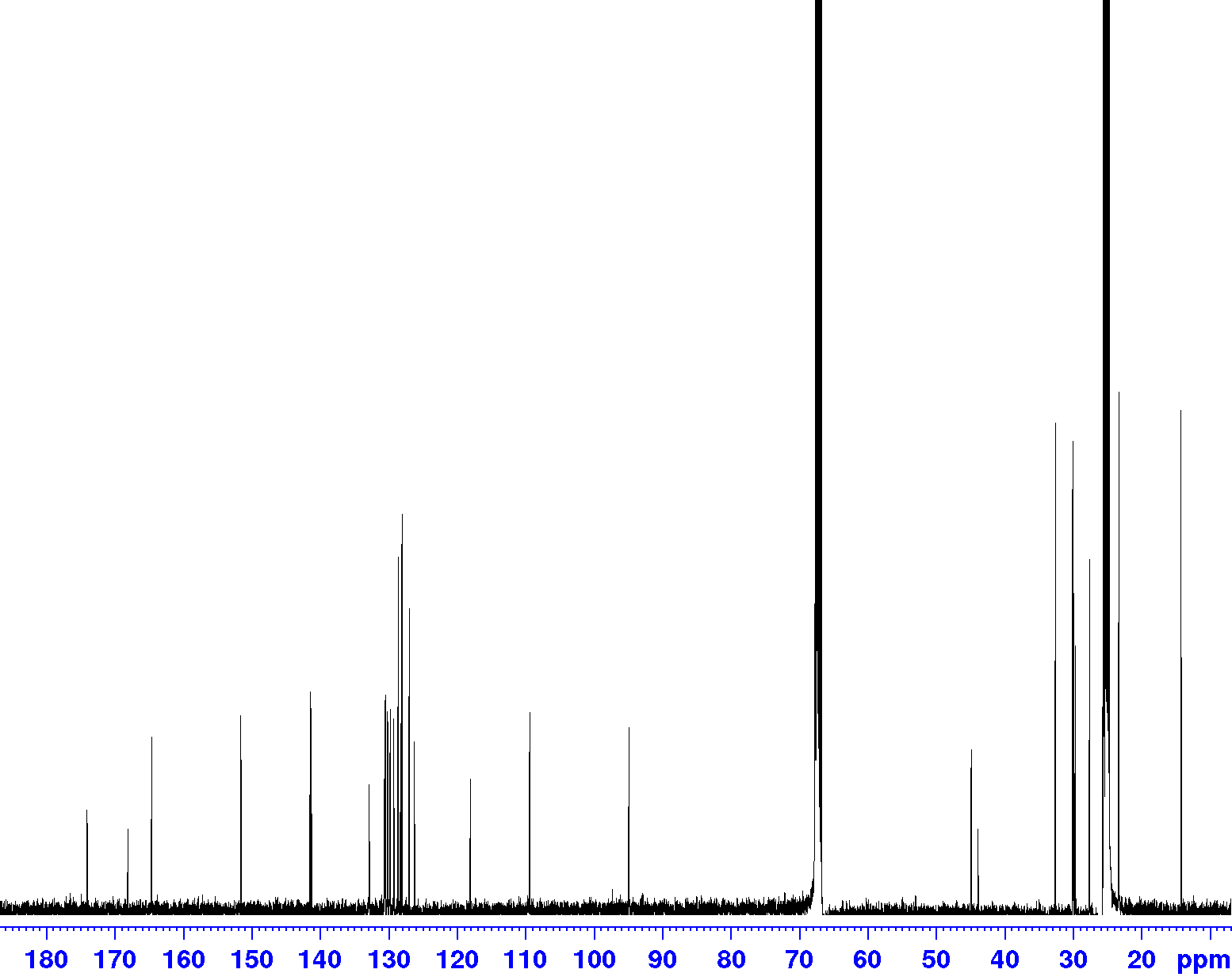


^13^C NMR (THF-d_8_, 100.6 MHz): *δ* 174.1, 168.1 (s, C-26, 27); 164.6 (s, C-25); 151.6 (s, C-11); 141.5 (s, C-20); 141.3 (s, C-6); 132.9 (d, C-9); 130.6 (s, C-10); 130.5 (d, C-8); 130.2 (d, C-2); 129.8 (d, C-7); 129.3 (s, C-3); 128.6 (d, C-22); 128.3 (s, C-4); 128.1 (d, C-21); 127.0 (d, C-23); 126.3 (s, C-5); 118.0 (s, C-29); 109.4 (d, C-1); 94.9 (d, C-24); 44.9 (t, C-12); 43.9 (s, C-28); 32.6 (t, C-17); 30.1 (t, C-15); 30.0 (t, C-16); 29.7 (t, C-13); 27.6 (t, C-14); 23.3 (t, C-18); 14.2 (q, C-19).

HMBC correlations: H-1→C-(3, 5, 6); H-2→C-(4, 6, 20); H-7→C-(3, 5, 9); H-8→C-(4, 9w, 10, 11w); H-9→C-(5, 7, 11); H-12→C-(6, 11, 13, 14); H-13→C-(12, 14, 15); H-14→C-(13, 15, 16); H-15→C-(14, 16); H-19→C-(17, 18); H-21→C-(20, 22, 23); H-22→C-(21); H-23→C-(20, 21, 22); H-24→C-(10, 11, 25, 26, 27).
